# Singlet Fission in Carbene‐Derived Diradicaloids

**DOI:** 10.1002/anie.202001286

**Published:** 2020-03-26

**Authors:** Tobias Ullrich, Piermaria Pinter, Julian Messelberger, Philipp Haines, Ramandeep Kaur, Max M. Hansmann, Dominik Munz, Dirk M. Guldi

**Affiliations:** ^1^ Department of Chemistry and Pharmacy Interdisciplinary Center for Molecular Materials (ICMM) Friedrich-Alexander-Universität Erlangen-Nürnberg Egerlandstraße 3 91058 Erlangen Germany; ^2^ Department of Chemistry and Pharmacy Interdisciplinary Center for Molecular Materials (ICMM), Allgemeine und Anorganische Chemie Friedrich-Alexander-Universität Erlangen-Nürnberg Egerlandstraße 1 91058 Erlangen Germany; ^3^ Fakultät für Chemie und Chemische Biologie Technische Universität Dortmund Otto-Hahn Straße 6 44227 Dortmund Germany

**Keywords:** carbenes, diradicaloids, energy conversion, self-assembly, singlet fission

## Abstract

Herein, we present a new class of singlet fission (SF) materials based on diradicaloids of carbene scaffolds, namely cyclic (alkyl)(amino)carbenes (CAACs). Our modular approach allows the tuning of two key SF criteria: the steric factor and the diradical character. In turn, we modified the energy landscapes of excited states in a systematic manner to accommodate the needs for SF. We report the first example of intermolecular SF in solution by dimer self‐assembly at cryogenic temperatures.

## Graphical abstract

**Diradicaloids** derived from cyclic carbenes are singlet‐fission molecules with exceptionally large diradicaloid character, which self‐assemble at cryogenic temperatures in solution.

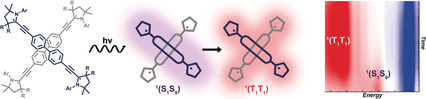

## Introduction

Singlet fission (SF) is a photophysical deactivation process, in which a singlet excited state transforms into two separated triplet exited states, involving a correlated triplet pair state ^1^(T_1_T_1_) with singlet multiplicity.^1^, ^2^ Overall, this process, which is based on the conservation of spin angular momentum, takes place on the picosecond or even sub‐picosecond timescales. Per se, it contrasts the more common triplet population driven by a spin‐forbidden intersystem crossing.^3^, ^4^, ^5^, ^6^ In a subsequent step, the spin‐entangled ^1^(T_1_T_1_) undergoes a loss of its spin coherence to yield two independently acting triplet excited states (T_1_ + T_1_).^7^, ^8^ Incentives to investigate this intriguing photophysical reaction are based on the application of SF‐materials in high‐performing photovoltaics. SF, as an exciton multiplication process, enables the maximum thermodynamic efficiency to be raised well beyond the Shockley–Queisser limit.^9^, ^10^, ^11^

Unfortunately, the number of molecular materials that undergo SF is limited.^12^, ^13^, ^14^ Hence, the lack of SF materials beyond conventional polyacenes constitutes a major bottleneck for the much‐needed progress in the field.^15^, ^16^, ^17^ In the quest for new SF materials, Michl et al. defined several fundamental thermodynamic requirements.^18^ Two molecular families are promising: Polyacenes and diradicaloids. Until today, most of the SF materials are, however, built around the acene platform, that is, ubiquitous tetracene and pentacene as well as their derivatives.^19^, ^20^, ^21^, ^22^, ^23^, ^24^ In stark contrast, investigations on diradicaloids remain hitherto largely restrained to computational studies.^25^, ^26^, ^27^ Notable exceptions are 1,3‐diphenylisobenzofurans and zethrenes, which both were also spectroscopically characterized.^28^, ^29^

More recently, Nakano and co‐workers hypothesized that a diradical character is essential for rendering materials SF operative and correlated their guidelines with the multiple diradical characters *y*_0_ and *y*_1_.^30^, ^31^, ^32^ The multiple diradical characters are quantum‐mechanically defined values relating to the occupation number of the first two unoccupied natural orbitals (NOs).^33^ They range from 0 to 1 and refer to the diradical and tetraradical characters, respectively. For example, a diradical index of *y*_0_=0 corresponds to a closed‐shell system, while *y*_0_=1 describes a “perfect” diradical system. 1,3‐diphenylisobenzofuran gives rise to a rather small diradical index of *y*_0_=0.21, while *y*_0_ is 0.43 for octazethrene.^28^, ^29^ Accordingly, Nakano et al. concluded that the thermodynamic requirements for SF are fulfilled neither in closed‐shell (*y*_0_=0) nor in tetraradical cases (*y*_0_≈*y*_1_).

Increasing the diradical character assists in stabilizing the triplet excited state, on one hand, and in increasing the SF exothermicity, on the other hand. To our knowledge, the most exothermic SF materials known to date are hexacene and a hexacene derivative with excess energies of 0.59 and 0.50 eV, respectively.^34^, ^35^, ^36^ By virtue of the scarce studies on molecular systems featuring high diradical characters, the area of highly exothermic SF remains largely unexplored.

Considering that SF leads to the formation of two electronic triplet states, it is often found in thin films, aggregates, or covalently linked dimers/oligomers. SF studies in the solid state and aggregates are typically intricate and systematic structural investigations are inherently challenging. Therefore, linking two separate SF chromophores, such as, for example, tetracenes and pentacenes, by molecular bridges has transformed into a viable strategy to study the nature of ^1^(T_1_T_1_) and (T_1_ + T_1_) even in solution.^37^, ^38^, ^39^, ^40^ A number of drawbacks go, however, hand‐in‐hand with the covalent linkages. For example, substantial alterations of the electronic couplings or energetics are likely scenarios, which, one way or the other, impact the SF dynamics. The presence of molecular bridges allows also for through‐bond couplings, which are absent in molecular crystals, and, which may be significantly stronger than through‐space coupling between the two monomers in a SF dimer. An additional concern is that triplet dissociation might be shut down due to the absence of entropic contributions and due to the lack of diffusion. Accordingly, molecular dimers, which lack through‐bond interactions, and, which allow tuning the inter‐chromophoric couplings, emerge as the ultimate choice for modeling SF.

In contrast to the aforementioned, it has been suggested that a triplet pair state may also be existent in monomers. Implicit is an internal conversion from a singly excited, bright state into a doubly excited, dark state of A_g_ symmetry and/or lower energy en‐route towards a correlated ^1^(T_1_T_1_) with strong inter‐triplet binding energies.^41^, ^42^, ^43^, ^44^ SF materials that feature doubly excited, singlet states, namely class III chromophores, are rare; most prominent examples are oligoenes and polyenes, in general, and carotenoids, in particular.^14^, ^45^, ^46^ This area of SF remains essentially unexplored and previous studies were dedicated to probing films, aggregates, and monomers rather than dimers. Wang and Tauber observed intermolecular SF in zeaxanthin aggregates without the involvement of the lowest doubly excited, singlet state, which is in line with the subsequent findings by Musser et al. with astaxanthin and semiconducting poly(thienylenevinylene).^45^, ^47^, ^48^ In contrast, Trinh et al. concluded that intermolecular SF in diphenyl‐dicyano‐oligoenes is triggered by the initial intramolecular formation of the correlated triplet pair state.^49^

Recently, we demonstrated the isolation of cyclic (alkyl)(amino)carbene (CAAC) stabilized diradicaloids with a carbene‐bridge‐carbene type molecular structure.^50^ Of great relevance is the fact that CAACs are known to stabilize radicals well.^51^ Importantly, a modular synthetic approach was chosen to allow for tuning the electronic properties by means of varying the carbene and the spacer. In a subsequent study, we used quantum‐chemical calculations to predict that CAACs are viable candidates as new SF materials.^52^ Herein, we report that CAAC diradicaloids, indeed, deactivate upon photoexcitation by SF. Unprecedented in the literature, this SF pathway is realized through non‐covalent π‐π‐interactions as a means to self‐assemble two separate SF chromophores to yield a dimer. Accordingly, we establish SF for materials with exceptionally large diradical character.^36^, ^53^

## Results and Discussion

**Figure 1:**
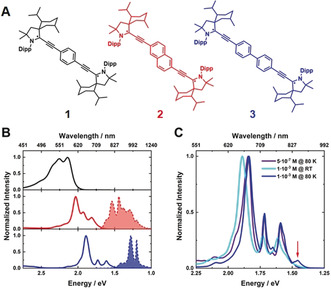
Molecular structures and steady‐state spectra. A) Structures of the phenyl‐bridged (**1**, black), naphthalene‐bridged (**2**, red), and biphenyl‐bridged (**3**, blue) bis‐CAAC diradicaloids. B) Normalized steady‐state absorption spectra at room temperature (solid line) and fluorescence spectra (dotted line) of **1** (black), **2** (red), and **3** (blue) measured at low concentrations (5×10^−7^ m) and 80 K upon excitation at 670 nm. C) Temperature‐ and concentration‐dependent normalized steady‐state absorption spectra of **3**. Steady‐state absorption at high concentrations (1×10^−3^ m) at room temperature (cyan) and 80 K (blue) as well as with low concentrations at 80 K (purple).

As presented in Figure 1 A, we used three different spacers, that is, phenylene (**1**), naphthalene (**2**), and biphenylene (**3**), as molecular bridges in the bis‐CAAC diradicaloids. Neither of these structural functionalities has been linked to SF so far. At room temperature, under argon atmosphere, NMR and UV/Vis spectroscopy showed no signs of decomposition even after three months in solution. Importantly, they stand out due to their photostability. Figure 1 B displays the steady‐state absorption and photoluminescence spectra at low concentrations. The absorption maxima of the three diradicaloids redshift significantly with increasing length of the linker. We attribute this to the decreasing energy difference between the highest occupied natural orbital (HONO) and lowest unoccupied natural orbital (LUNO), leading to a sizeable diradical character.

For **2** and **3**, we found broad near infrared (NIR) fluorescence patterns with markedly large Stokes shift, while **1** deactivates exclusively by a non‐radiative pathway (Figure 1 B). The fluorescence for **2** is at room temperature undefined, broad, and weak (Supporting Information, Figure S2). A distinct vibrational progression was resolved after cooling to cryogenic temperatures. This is in stark contrast to the findings made for **3**, which already exhibits at room temperature vibrationally resolved, fine‐structured fluorescence with maxima at 970 and 1035 nm. Notable is the asymmetric shape of the fluorescence, which is rationalized by the coexistence of *E*‐ and *Z*‐isomers.^50^ Upon cooling to 80 K, the relative ratio of the absorption bands changes in favor of the absorption bands at 715 and 770 nm with respect to the band at 675 nm and the maxima shift to higher wavelengths (Figure 1 C). At room temperature, changes in the concentration had no appreciable impact on the absorption features. But, at cryogenic temperatures, a new absorption feature evolved at 850 nm together with a shoulder at 790 nm.

Albeit weak, an additional redshift of the absorption maxima was observed. The aforementioned spectroscopic observations are consistent with intermolecular interactions in the ground state and, hence, indicate the formation of dimers/oligomers. Similar effects were seen for **2**, where a distinctive shoulder appeared at 730 nm (Supporting Information, Figure S1).^54^

**Figure 2:**
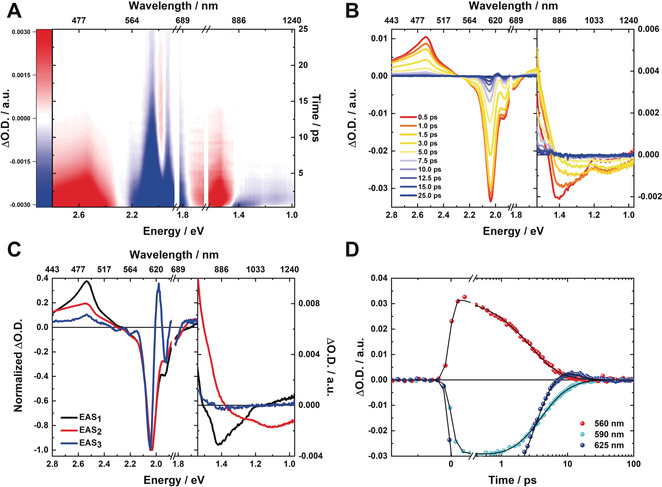
Photophysical deactivation of **2** at room temperature. A) Zero‐point‐ and chirp‐corrected fsTA heat map in the visible and near‐infrared parts of the optical spectrum obtained for photoexcitation at 670 nm and concentration of 5×10^−6^ m. B) Corresponding spectral slices illustrating the excited‐state dynamics. C) Evolution‐associated spectra obtained by global analysis. D) Single‐wavelength kinetics at 560 (red), 590 (cyan), and 625 nm (blue) as well as global fit to the data (black line).

To observe the deactivation dynamics, femtosecond transient absorption (fsTA) spectroscopy was performed; first, at room temperature and at low concentrations. fsTA results are exemplified for **2** in Figure 2. Upon photoexcitation at 670 nm, the singly excited, optical bright S_2_ state is formed with a characteristic maximum at 490 nm and characteristic minima at 610, 640, 970, and 1110 nm. Notably, the minima at 970 and 1110 nm correspond to stimulated emission. At the same time, a weak positive TA signal superimposes the fluorescence in the region of 0.95 and 1.20 eV. Within the first picoseconds, both of these stimulated fluorescence minima shift to longer wavelengths. Implicit is a relaxation of the hot S_2_ state. Next, the relaxed S_2_ state decays with a lifetime of 2.3 ps. Hand‐in‐hand with the latter decay is the evolution of a sharp and weak positive transient with a maximum around 625 nm, that is, in the region of the ground‐state bleaching. The latter fingerprint is a diagnostic marker for the population of the doubly excited, dark S_1_ state.^47^ This is in line with our computational calculations (see below), which predict the interconversion between the initially populated S_2_ state and the doubly excited state S_1_, and, which, in turn, confirms the assignment of class III SF chromophores. Interesting is the fact that the negative TA in the NIR is absent during these times. We conclude that S_1_ deactivates non‐radiatively by internal conversion (namely, absence of Herzberg–Teller emission), whereas the observed fluorescence originates exclusively from the one‐photon‐allowed S_2_. The corresponding S_1_ lifetime of 5.7 ps, during which the singlet ground state is reinstated, is relatively long in comparison to the family of polyenes with dynamics in the sub‐ps time domain.^55^ It is very likely that the rigid molecular structure and the reduced degree of vibrational freedom are responsible for the rather long lifetime. **1**, **2**, and **3** share the same deactivation behavior (Supporting Information, Figures S3 and S4), which involves a low‐lying doubly excited state due to the high tetraradical character. Interestingly, the spectral fingerprint corresponding to the doubly excited S_1_ state is always found within the range of the ground‐state bleaching. Interesting is the fact that **3** features an additional broadband transient in the NIR including a 1110 nm maximum, to which the stimulated emission at 970 and 1035 nm is superimposed; these are consistent with the fluorescence maxima in the steady‐state fluorescence spectrum. In the case of **1**, the NIR is silent, that is, neither positive nor negative TA signals were observed.

**Figure 3:**
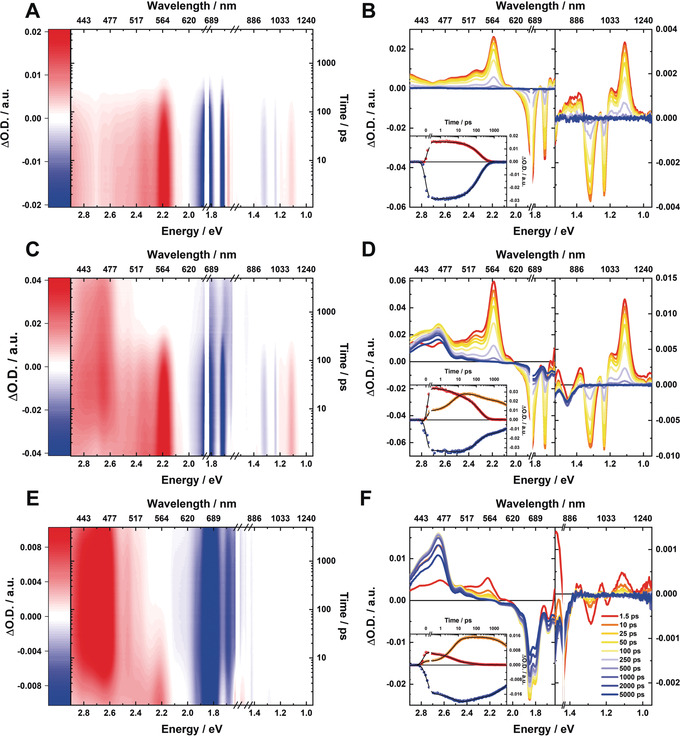
Concentration and excitation dependency of **3** at cryogenic temperatures (80 K) A) Zero‐point‐ and chirp‐corrected fsTA heat map in the visible and near‐infrared part of the optical spectrum at low concentrations (5×10^−6^ m), obtained upon photoexcitation at 670 nm. B) Corresponding absorption–time profiles illustrating the excited‐state dynamics. The inset shows single‐wavelength kinetics at 575 (red) and 655 nm (blue) as well as global fit to data (black line). C) fsTA heat map established for increased concentrations (1×10^−3^ m) D) Corresponding absorption–time profiles illustrating the excited‐state dynamics. The inset shows single wavelength kinetics at 470 (orange), 560 (red), and 690 nm (blue) as well as global fit to data (black line). E) fsTA heat map obtained by change of the pump wavelength to 850 nm, corresponding to the characteristic absorption band maximum formed at high concentrations, that is, 1×10^−3^ m. F) Corresponding absorption–time profiles illustrating the excited‐state dynamics. The inset shows single‐wavelength kinetics at 470 (orange), 560 (red), and 690 nm (blue) as well as global fit to data (black line).

Subtle changes evolved, however, upon cooling to cryogenic temperatures (for example, **3** in Figure 3 A,B). Most notable, the TA maximum, which corresponds to S_2_, is more pronounced and redshifted by 10 nm in reference to the room‐temperature measurements. In line with the steady‐state fluorescence experiments, the NIR TA features become visible in the form of a negative transient. Overall, the singlet excited state decays monoexponentially with a lifetime of 160 ps. Considering the absence of the doubly excited S_1_ state, which was seen in the room‐temperature experiments, we conclude that only the bright S_2_ is populated at low temperatures.

Substantial changes developed for concentrated solutions (Figure 3 C,D). Now, the long‐lived 470 nm transient is prominently discernable in the TA and rises with a markable rate of 5.3 ps. We assign this spectral feature to the correlated triplet pair state: ^1^(T_1_T_1_). We conclude that the triplet population occurs by SF, because the observed ultrafast formation proceeds in the absence of any heavy atoms. Notably, the SF rates for **2** and **3** are one order of magnitude slower compared to what has been reported for acene crystals,^56^ but are still within the range commonly found for SF materials.^13^, ^57^

We hypothesize that in **3** the strong SF exothermicity decelerates the overall SF. Notably, the 850 nm absorption characteristics, which are discernable in the steady‐state absorption spectra at high concentrations (Figure 1 C), correlate with the minimum seen in the ground‐state bleaching. Throughout the overall deactivation, the ground‐state absorption pattern is subjected to a shifting to longer wavelengths and a broadening; the end point of these changes is the complete singlet excited manifold decay.

A change of the excitation wavelength to 850 nm led, at low temperature and high concentrations, to remarkable changes in the TA. Now, the fsTA plot is dominated by the triplet absorption spectrum, while S_2_ is drastically quenched. It decays with a lifetime of 5.3 ps, which relates to the SF rate. Hand‐in‐hand with this goes a quenching of the fluorescence in the NIR region. Of great relevance is the splitting of the ground‐state‐bleaching minimum at 675 nm into two maxima, namely at 670 and 685 nm. Here, the overlap of wave functions stemming from interacting monomers leads to a split into two states, commonly referred to as Davydov splitting.^58^ Davydov splitting has been observed for several SF materials, such as acene single crystals, diketopyrrolopyrroles, and pentacene dimers.^59^, ^60^, ^61^ In stark contrast to the 670 nm excitation, the ground‐state bleaching lacks any appreciable shifts as a function of time. The modus operandi is based on the selective excitation of dimers, which absorb at 850 nm, and, which coexist with the isolated monomers. Accordingly, the TA spectra in Figure 3 C,D obtained upon excitation at 670 nm, represent a superposition of TA of both monomers and dimers.^62^ Considering that the deactivation of the dimers proceeds without the involvement of the doubly excited S_1_ state, SF originates form the bright singly excited S_2_ state. The detailed inspection of the kinetics in the range of ground‐state bleaching reveals the ground‐state recovery within 5.3 ps (Figure 3 F), which occurs in parallel to the formation of ^1^(T_1_T_1_) and decay of S_2_. Our observation prompts to the generation of more than a single triplet excited state, that is, ^1^(T_1_T_1_) as such a rapid ground‐state recovery is only rationalized by multiple excitation generation. In turn, it clearly supports SF rather than conventional intersystem crossing. Together with the absence of S_1_, an intramolecular mechanism is excluded.

Essentially, the same results were gathered for **2** (Supporting Information, Figure S7). Again, the TA results are in sound agreement with intermolecular SF (see above). They are a superposition of monomeric and dimeric diradicaloid TA signals in the low temperature and high concentration regime. For **2**, SF is slightly faster with a corresponding lifetime of 4.5 ps. A faster SF rate agrees with the fact that the ^1^(T_1_T_1_) formation is considerably less exothermic compared to **3** due to the lower diradicaloid character. No unambiguous evidence was found in support for a Davydov splitting; instead only a significant broadening evolved.

In stark contrast, **1** lacks, at any given time delay, the presence of any long‐lived species in the TA spectra, which might correspond to ^1^(T_1_T_1_). Even cooling to 80 K and/or increasing the concentrations to 1×10^−3^ m, led to the same deactivation patterns that were found at low concentrations (Supporting Information, Figures S5 and S6). The only notable difference is a biphasic decay for the singlet excited manifold with lifetimes of 340 and 1200 ps. Nevertheless, the evolution‐associated spectra (EAS) look almost identical, which, in turn, leads to the conclusion that the doubly excited S_1_ state is not involved in the deactivation process. A plausible explanation is that different isomers lead to the observed biphasic decay.

In turn, we complemented our experimental results with density functional theory (DFT) calculations to investigate the structure of the dimers (Supporting Information, Figure S15). The optimized geometric parameters for dimers of **2** and **3** with various multiplicities, that is, singlet, triplet, and quintet, were very similar. No sizeable minimum structures were, however, found for dimers of **1**. The bulky carbene groups allow for π‐π‐interactions only when large conjugated bridges like in, for example, **2** and **3**, are present. A perpendicular arrangement with a distance of 3.40 Å between the centroids of the naphthalene linker in **2**, which is on the same order of magnitude as found for pentacene (3.5 Å) and TIPS‐pentacene (3.3 Å),^56^ dominates the dimeric structure. Notably, the steric demand of the CAACs efficiently prevents aggregation of more than two monomers of **2** and **3**. Accordingly, the underlying redshift in the absorption spectra relates to the delocalization of electron density over both interacting SF molecules. This is perfectly in line with the steady‐state absorption as well as fsTA experiments in terms of temperature and concentration dependences. Overall, these trends demonstrated the absence of ground‐state complex formation even at high concentrations. This, in turn, explains also the absence of SF in **1**, although it is feasible to occur on thermodynamic grounds.

**Figure 4:**
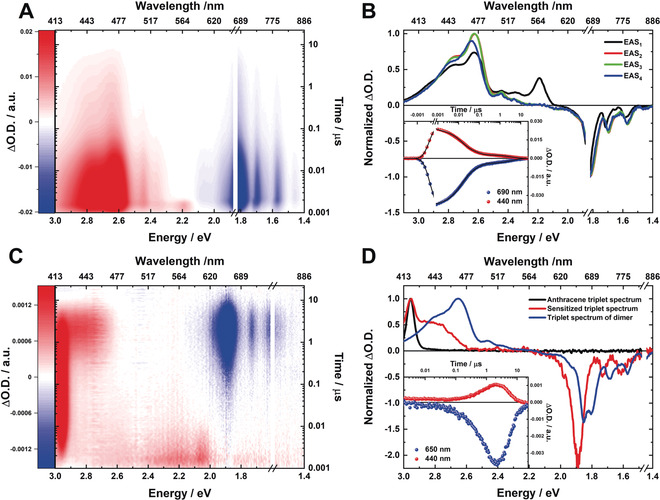
^1^(T_1_T_1_) deactivation and triplet‐triplet sensitization of **3**. A) nsTA heat map in the visible part of the optical spectrum at 80 K and high concentration (1×10^−3^ m), obtained upon photoexcitation at 670 nm. B) Corresponding normalized EAS together with representative kinetics (inset) at 690 (blue) and 440 nm (red) as well as global fit to the data (black line). C) nsTA heat map of triplet‐triplet sensitization experiment (3×10^−5^ m) with anthracene (8×10^−5^ m) at roomtemperature and excitation at 387 nm. D) Normalized TA spectra of anthracene (black), sensitized triplet (red) and ^1^(T_1_T_1_) of the **3** dimer (blue) obtained at a time delay of 2.2 μs. The inset shows corresponding kinetics of the triplet‐triplet sensitization experiments at 690 (blue) and 440 nm (red).

To further probe the deactivation of ^1^(T_1_T_1_), we employed nanosecond transient absorption (nsTA) spectroscopy: Notably, ^1^(T_1_T_1_) of **3** decays triphasic with lifetimes of 13, 64, and 4000 ns with almost identical TA spectra (Figure 4 A,B). Interestingly, the third EAS of **3** is slightly blueshifted by around 5 nm relative to EAS_2_ and EAS_3_. Such multiphasic decays are commonly associated with SF materials. Implicit are different intermediates and, potentially, the triplet decorrelation into two independent T_1_. One of the triplet pair intermediates, in addition to ^1^(T_1_T_1_) with its overall singlet multiplicity, is the recently observed triplet pair quintet state ^5^(T_1_T_1_).^63^, ^64^, ^65^ Unfortunately, neither fs‐ nor nsTA spectroscopies assist in supporting our conclusions about the spin multiplicity of the triplet pair state and triplet pair intermediates.^66^ Still, the fact that we note a triexponential decay is another strong indication for the occurrence of SF, since regular triplet excited states deactivate usually in a monoexponential fashion. **2** gave rise to the same triphasic decay, albeit with substantially longer lifetimes of 31 ns, 430 ns, and 12.9 μs. Hand‐in‐hand with the longer lifetimes is the absence of the blueshift of the triplet maximum at 470 nm in the third and final EAS of **2** (Supporting Information, Figure S11).

To validate the triplet nature of the long‐lived transient, we performed triplet‐triplet sensitization experiments. We selected anthracene, which is a well‐known and inert triplet sensitizer. As shown in Figure 4 C,D for **3**, the depletion of the anthracene triplet excited state with its maximum at 420 nm occurs simultaneously with the growth of a new positive TA feature between 400 and 480 nm as well as minima at 660, 715, and 780 nm. All of the minima correspond well with the absorption maxima of the monomer at room temperature. Compared to the triplet spectrum of the dimer **3**, substantial differences are discernable. First, the ground‐state bleaching of the dimer shows significant changes compared to the triplet spectrum of a non‐interacting monomer. Second, the triplet maximum of dimer **3** is clearly shifted to longer wavelengths and exhibits shoulders at 450, 510, and 530 nm. As a rationale, we consider changes in the electronic properties due to intermolecular interactions. In most cases, the TA spectra of ^1^(T_1_T_1_) and ^3^(T_1_S_0_) are quite similar,^64^ with only subtle shifts in the triplet TA maximum. Importantly, the triplet excited state decay of 3.6 μs upon photosensitization is in good agreement with the long‐lived component of the triexponential ^1^(T_1_T_1_) decay. This points, on one hand, to the decorrelation of ^1^(T_1_T_1_) into two independently acting triplet states, namely (T_1_ + T_1_). On the other hand, it excludes the possibility that the presence of different dimer aggregates (namely, *E*/*E*, *E*/*Z*, *Z*/*Z*, amongst others) is responsible for the overall deactivation. In this particular case, the corresponding lifetimes would be expected to be in the same range. Overall, it is impossible to dissect the spectral shape of the triplet pair state spectrum and to directly relate it to that of an isolated, fully independent triplet state.^67^, ^68^ Still, the similarities between sensitized and observed TA spectra serve as strong evidence for the presence of a triplet excited state (For **2**, see Figure S8 of the Supporting Informatino). More importantly, it suggests that the two triplets coupled within the doubly excited S_1_ state are strongly correlated, since the underlying singlet state reveals no particular triplet characteristics at all.

**Figure 5:**
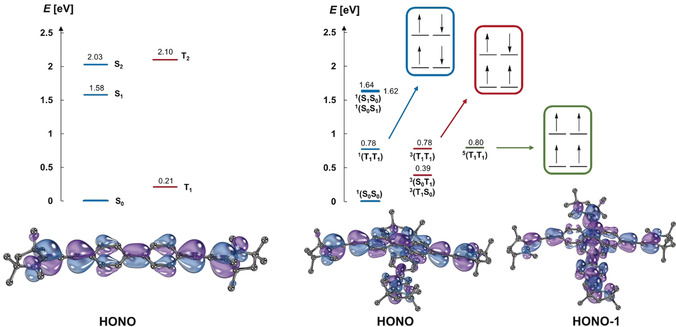
Electronic structure of **3** as a monomer (left) and as dimers (right) as predicted by CASSCF/NEVPT2 calculations.

To better understand the photochemistry of both monomers and dimers, we performed further quantum‐chemical calculations (CASSCF/NEVPT2) to characterize the key electronic states in both their adiabatic and diabatic representations. For the monomer of **3**, we reported a cumulenic ground state S_0_ with a large diradical character (*y*_0_=0.78).^52^ Furthermore (Figure 5, left), the excitation to the strongly multiconfigurational S_1_, which is sometimes referred to as the “doubly excited state”, is predicted to be forbidden (*E=*1.58 eV, *λ* ^calc^=784 nm, *f*_osc_=0.0), whereas the vertical transition to S_2_ is allowed (*E*=2.03 eV; *λ* ^calc^=610 nm, *f*_osc_=1.0). T_1_ is remarkably low in energy (*E*=0.21 eV) as is also in line with the very high diradical index. Overall, this electronic structure resembles that of a class III chromophore. An equivalent picture was gathered for **2** in its monomeric form (*y*_0_=0.37). In this case, our calculations confirm (*λ* ^exp^=609 nm; see Figure S18 in the Supporting Information) that the excitation into S_2_ is allowed (*E*=2.17 eV; *λ* ^calc^=572 nm, *f*_osc_=1.0), whereas the vertical transition to the dark S_1_ is forbidden (*E*=2.10 eV; *λ* ^calc^=590 nm, *f*_osc_=0.0).

Upon dimerization of **3**, we find that the electronic structure of the ground state differs only slightly from that of the two individual monomers. The HONO‐1 and HONO, which are located on either of the two dimers, are degenerate with a slight increase of the diradical character associated with each monomer (*y*_0_=0.80 and 0.90 vs. *y*_0_=0.78 in the monomer).

Strikingly, the electronic structure of the excited states changes vastly: The first excited singlet state of the dimer is strongly stabilized relative to that of the monomer with *E*=0.78 eV (*λ* ^calc^=1599 nm) and is essentially degenerate with *E*=0.78 eV for the triplet state and *E*=0.80 eV for the quintet state. These three states share strong tetraradical character according to calculations using either an active space of 12 electrons populating 12 orbitals (12e/12o; weight of the tetraradical configuration state function (CSFs): singlet, 0.35; triplet, 0.52; quintet, 0.87) and as well in a minimal active space of 4e/4o (CSFs: singlet, 0.96; triplet, 0.97; quintet, 1.0). Within the simplified diabatic picture (Supporting Information, Table S8), these states are best described as correlated triplet pairs, namely ^1^(T_1_T_1_), ^3^(T_1_T_1_), and ^5^(T_1_T_1_), as proposed by Kollmar and, thus, complementing our experimental observation of a ^5^(T_1_T_1_) state for covalently linked bispentacenes.^62^, ^64^, ^65^, ^69^ Furthermore, the ^1^(S_0_S_1_) and ^1^(S_1_S_0_) states, which relate to the experimentally observed Davydov splitting in concentrated solutions (see above, *λ* ^exp^=670 and 685 nm), are located at 1.62 eV (764 nm) and 1.64 eV (755 nm), respectively. The diabatic representation reveals insignificant couplings between ^1^(S_0_S_0_) and ^1^(T_1_T_1_) as well as weak direct couplings between ^1^(S_0_S_1_) and ^1^(T_1_T_1_). Furthermore, both ^1^(S_0_S_1_) as well as ^1^(T_1_T_1_) couple with two charge‐transfer states, that is, ^1^(CA) and ^1^(AC). Overall, the computational results indicate a mediated SF mechanism involving higher lying states. Eventually, computational analyses suggest the favorable dissociation into two independent T_1_, since the binding energy *E*_b_, which is approximated as the energy difference between ^1^(T_1_T_1_) and ^5^(T_1_T_1_), is very small (*E*_b_≈0.80 eV−0.78 eV≈0.02 eV).^70^ We conclude that the ^5^(T_1_T_1_) formation proceeds on timescales that are compatible with SF. For dimers of **2**, we find the equivalent diabatic and adiabatic pictures with tetraradicaloid and degenerate ^1^(T_1_T_1_) [1.19 eV], ^3^(T_1_T_1_) [1.19 eV], and ^5^(T_1_T_1_) [1.19 eV] (Supporting Information, Figure S18 and Table S7). It is also interesting to note the agreement between a considerably faster decay for **3** with 4000 ns in comparison to **2** with 12.9 μs and a weaker bonding between the two monomers of **3** (*E*_b_=−0.02 eV) relative to those of **2** (*E*_b_≈0.00 eV). Finally, the calculation of the spin‐orbit coupling matrix elements (less than 0.5 cm^−1^) by quasi‐degenerate perturbation theory (QDPT) confirms that intersystem crossing to the triplet multiplicity is unlikely for both the monomers as well as the dimers (Supporting Information, Table S9).

## Conclusion

We used fs‐ and nsTA spectroscopy complemented by computational calculations to study modular synthesizable carbene‐derived open‐shell systems. We observed intermolecular SF in dimers, which were formed in solutions at lower temperatures and, which are chemically stable under irradiation. Dimerization allowed the formation of a correlated triplet state ^1^(T_1_T_1_) with singlet multiplicity, which is degenerate with the related ^3^(T_1_T_1_) and ^5^(T_1_T_1_).

## Conflict of interest

The authors declare no conflict of interest.

## Supporting information

As a service to our authors and readers, this journal provides supporting information supplied by the authors. Such materials are peer reviewed and may be re‐organized for online delivery, but are not copy‐edited or typeset. Technical support issues arising from supporting information (other than missing files) should be addressed to the authors.

Supplementary
