# Supplementary material for: Singlet Fission in Carbene‐Derived Diradicaloids
Source: Angew Chem Int Ed Engl. 2020 Mar 26;59(20):7906–14. doi: 10.1002/anie.202001286 (PMC7317569; doi:10.1002/anie.202001286)
Supplement: Supplementary file 1 — Supplementary [file ANIE-59-7906-s001.pdf]

Angewandte Chemie
Eine Zeitschrift der Gesellschaft Deutscher Chemiker

Supporting Information

Singlet Fission in Carbene-Derived Diradicaloids

Tobias Ullrich, Piermaria Pinter, Julian Messelberger, Philipp Haines, Ramandeep Kaur, Max M. Hansmann, Dominik Munz,* and Dirk M. Guldi**

anie_202001286_sm_miscellaneous_information.pdf

Table of Contents

**Section S1. Spectroscopical Methods – General**	**S3**
**Section S 2. Steady State Spectroscopy**	**S4**
**Section S 3. Femtosecond Transient Absorption Spectroscopy**	**S6**
**Section S 4. Nanosecond Transient Absorption Spectroscopy**	**S14**
**Section S 5. Computational Details – General**	**S18**
**Section S 6. Configuration State Functions (CSFs)**	**S21**
**Section S 7. Diabatic Electronic States**	**S46**
**Section S 8. XYZ Coordinates**	**S54**
**Section S 9. Supplementary References**	**S81**

Section S1. Spectroscopical Methods – General

All compound were prepared as reported previously.[1] For the photophysical studies, samples were prepared in a glove box under inert atmosphere (argon- or dinitrogen) and placed in subsequently sealed cuvettes with different path lengths (depending on the chosen concentration). For low temperature measurements, cuvettes were placed in a liquid dinitrogen cryostat (Oxford Instruments: Optistat DN). 3-Methylpentane was used as solvent, since it forms an optical transparent glass at cryogenic temperatures. It was dried, degassed and purified by distillation over potassium under inert atmosphere. All investigated compounds did not show any signs of decomposition in solution over the course of more than a month.

Steady state absorption spectra were recorded by using a Varian Cary 5000 spectrometer. Steady state fluorescence spectra in the NIR were measured with a Horiba FluoroMax3. The excitation wavelength was set to 670 nm.

All fs- and nsTA spectra were carried out with an amplified Ti:Sapphire CPA 2110 fs laser system (Clark MXR:output 775 nm, repetition rate 1 kHz, 150 fs pulse duration) using a customized TA pump/probe detection system (Ultrafast systems: Helios, Eos). The excitation pulses at 550, 670 and 850 nm were generated *via* non-collinear parametric amplification (NOPA) and energy per pulse was reduced to 100 and 200 nJ, respectively.

Triplet-triplet sensitization experiments were performed with anthracene, purchased from a common supplier and further purified by sublimation. As excitation wavelength the second harmonic of the fundamental laser wavelength was used and reduced to an energy per pulse of 400 and 600 nJ, respectively.

All fs- and nsTA data were fitted *via* global analysis, performed with the open-source software package GloTarAn. Therefore, a sequential kinetic model was applied to fit the data. The dispersion of the instrument response function was modeled and taken into account.

Section S2. Steady State Spectroscopy

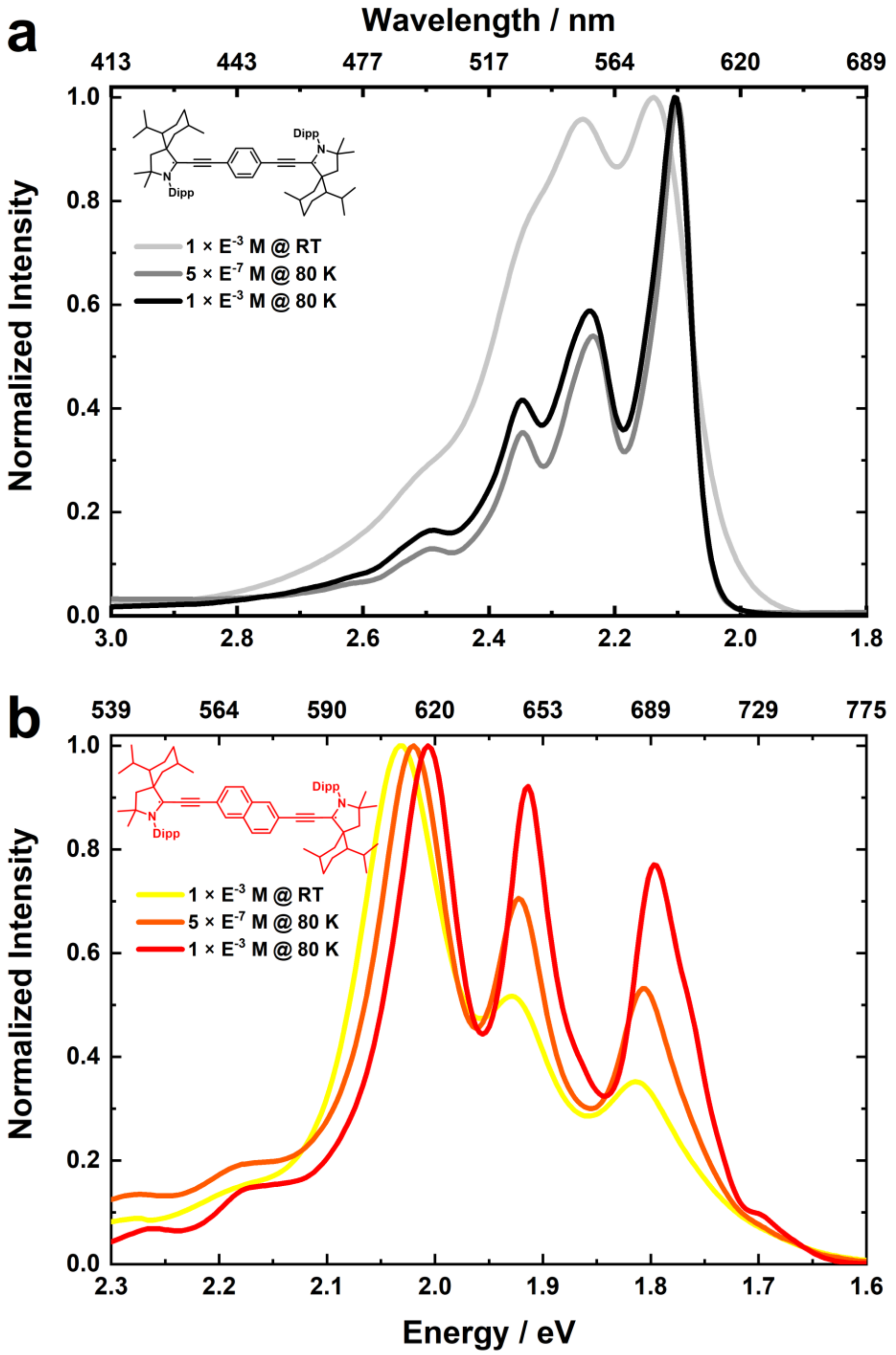

Figure S1: **a**, Normalized steady-state absorption spectra of **1** at room temperature and high concentration (1×10^{-3} M, white) as well as 80 K measured at low concentration (5×10^{-7} M, grey) and high concentration (1×10^{-3} M, black). **b**, Normalized steady-state absorption spectra of **2** at room temperature and high concentration (1×10^{-3} M, yellow) as well as 80 K measured at low concentration (5×10^{-7} M, orange) and high concentration (1×10^{-3} M, red).

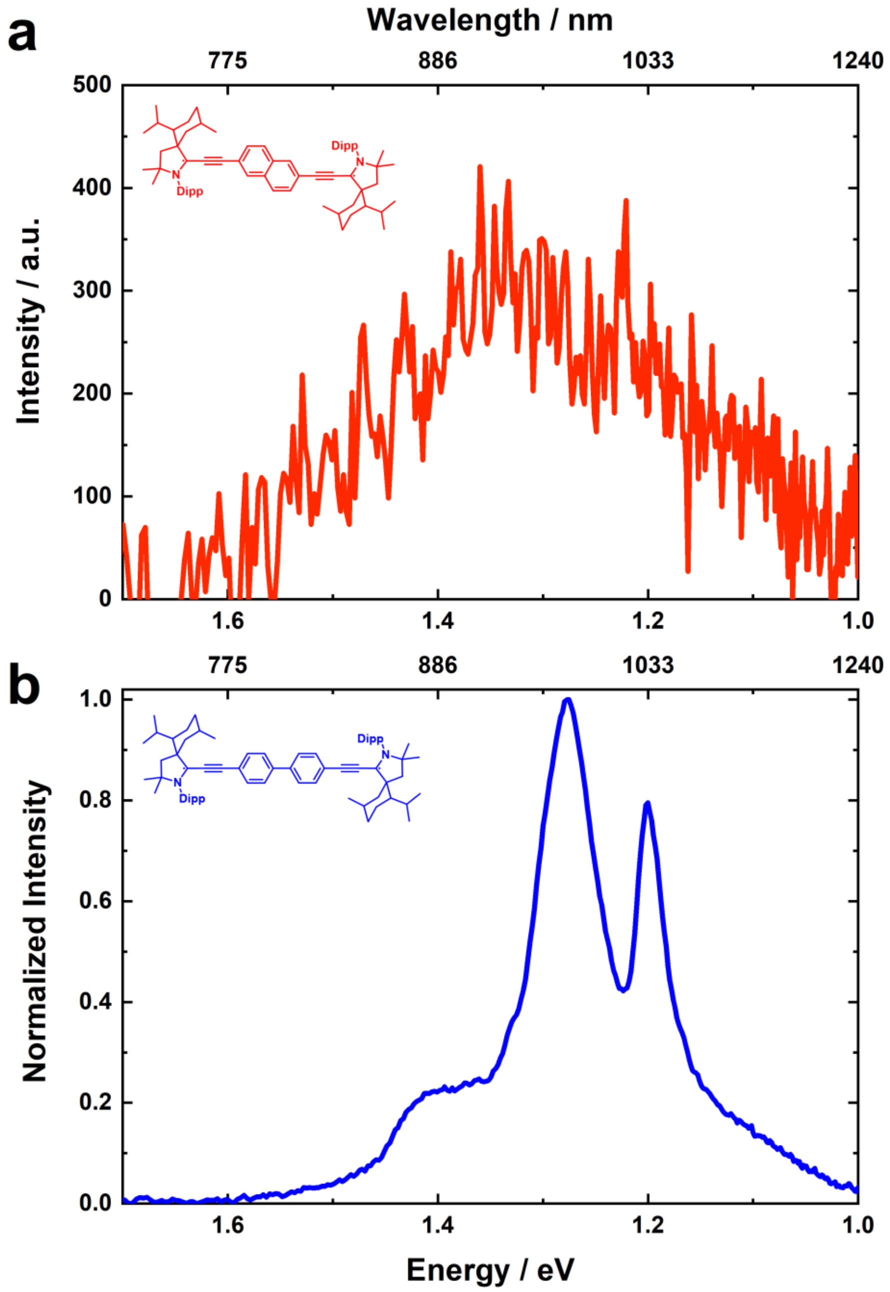

Figure S2: **a,** Steady-state fluorescence spectra of **2** measured at room temperature and concentration of 5×10^{-7} M upon excitation at 670 nm. **b**, Normalized steady-state fluorescence spectra of **3** measured at room temperature and and concentration of 5×10^{-7} M upon photoexcitation at 670 nm.

Section S3. Femtosecond Transient Absorption Spectroscopy

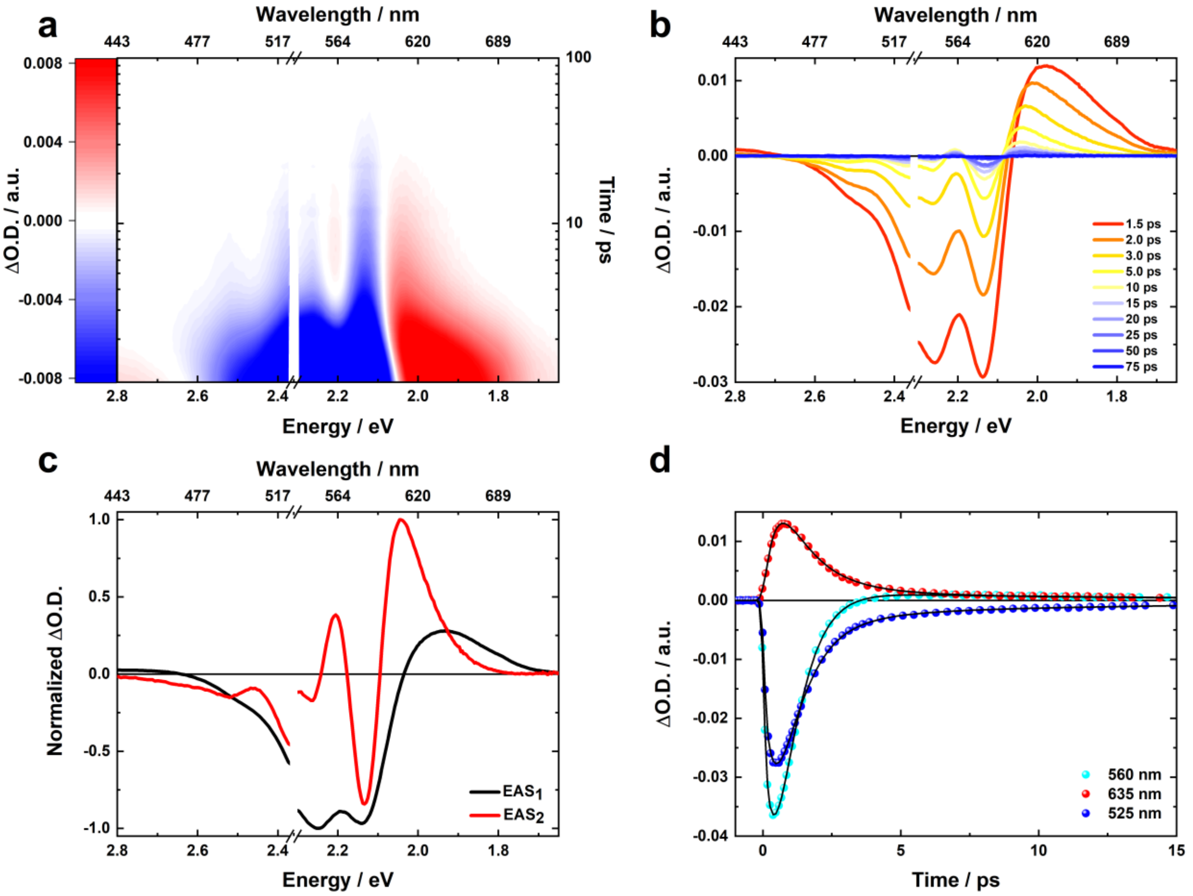

Figure S3: **a**, Zero point and chirp corrected fsTA heat map in the visible part of the optical spectrum of **1** obtained at room temperature upon photoexcitation at 530 nm and concentration of 1×10^{-3} M. **b**, Corresponding spectral slices illustrating the excited state dynamics. **c**, Evolution associated spectra obtained by global analysis. **d**, Single wavelength kinetics at 525 (cyan), 560 (blue) and 635 nm (red) as well as fit to the data (black line).

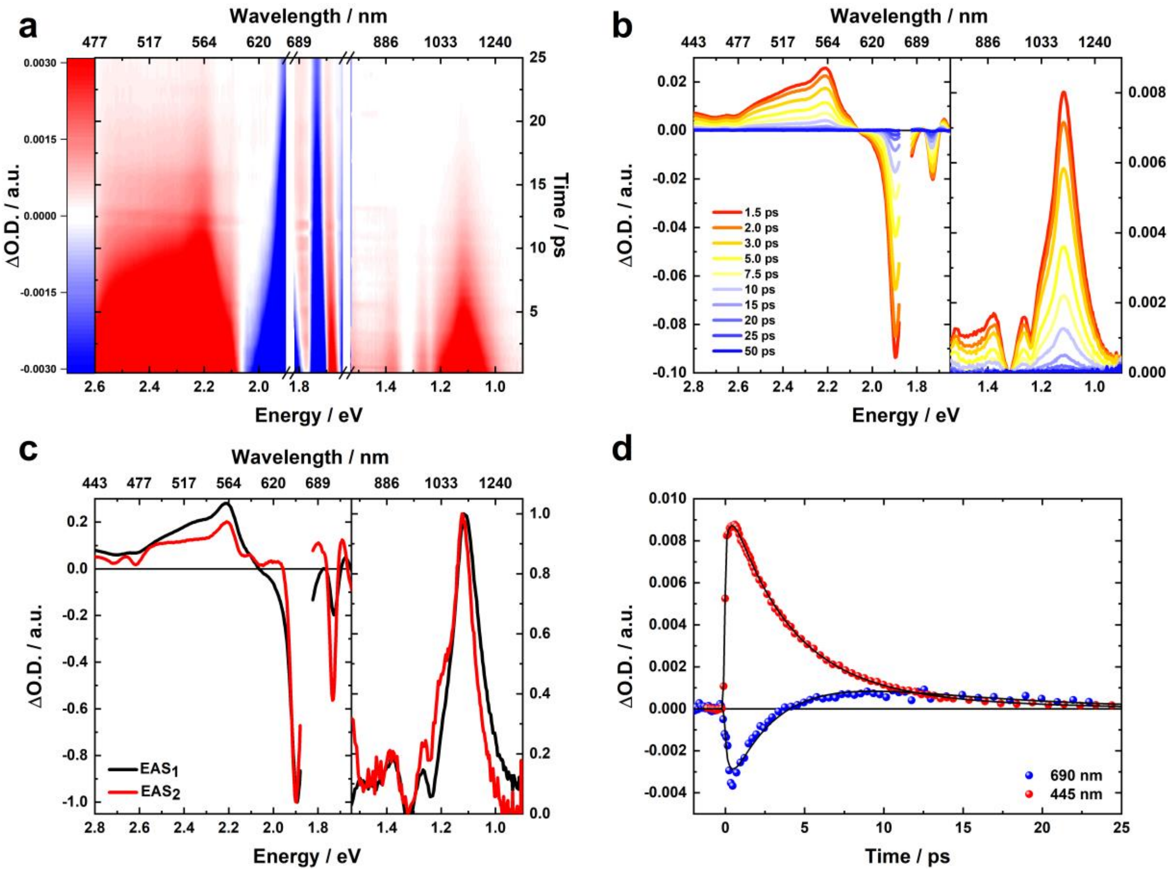

Figure S4: **a,** Zero point and chirp corrected fsTA heat map in the visible and near-infrared part of the optical spectrum of **3** obtained at room temperature upon photoexcitation at 670 nm and concentration of $1{\times}10^{-3}$ M. **b**, Corresponding spectral slices illustrating the excited state dynamics. **c**, Evolution associated spectra obtained by global analysis. **d**, Single wavelength kinetics at 445 (red) and 690 nm (blue) as well as fit to the data (black line).

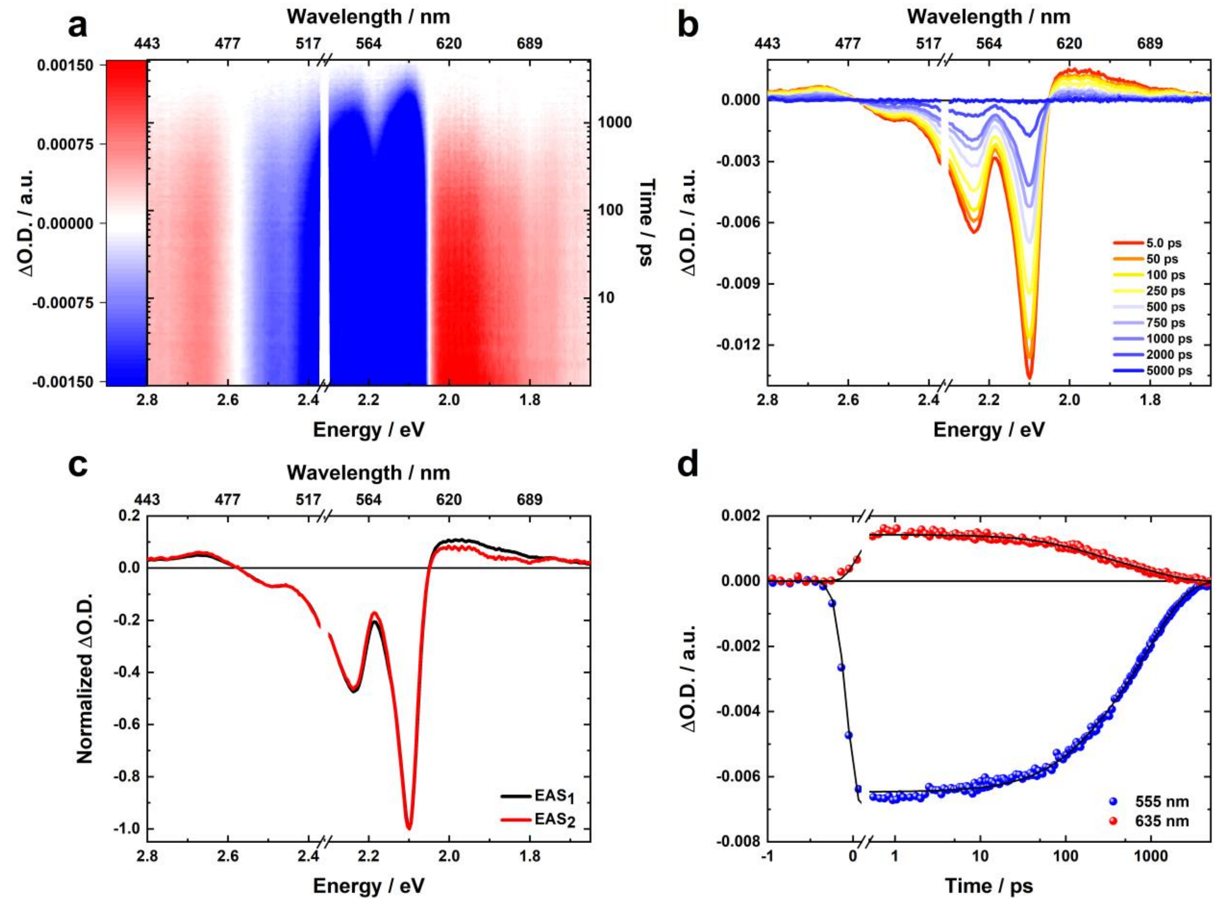

Figure S5: **a**, Zero point and chirp corrected fsTA heat map in the visible part of the optical spectrum of **1** obtained at 80 K upon photoexcitation at 670 nm and concentration of 5×10^{-6} M. **b**, Corresponding spectral slices illustrating the excited state dynamics. **c**, Evolution associated spectra obtained by global analysis. **d**, Single wavelength kinetics at 555 (blue) and 635 nm (red) as well as fit to the data (black line).

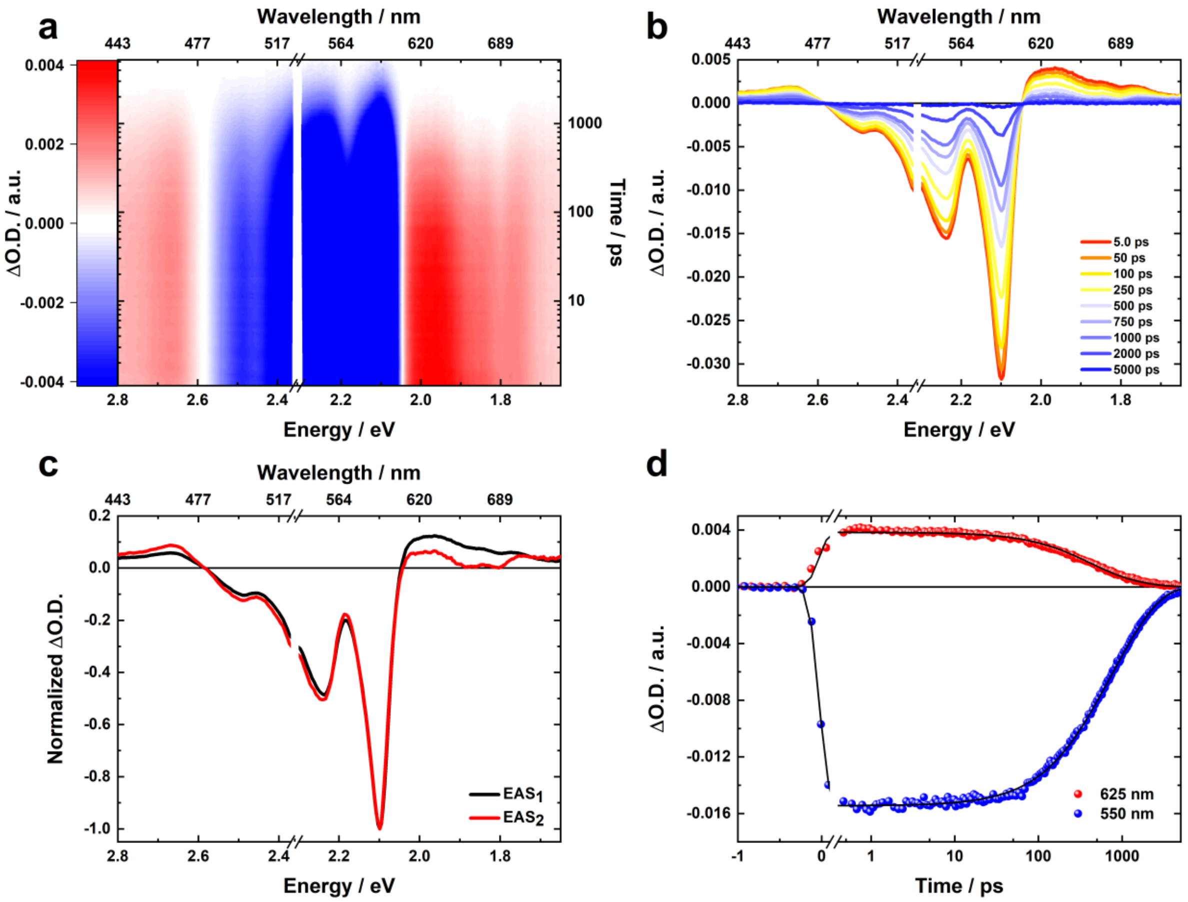

Figure S6: **a**, Zero point and chirp corrected fsTA heat map in the visible part of the optical spectrum of **1** obtained at 80 K upon photoexcitation at 670 nm and concentration of 1×10^{-3} M. **b**, Corresponding spectral slices illustrating the excited state dynamics. **c**, Evolution associated spectra obtained by global analysis. **d**, Single wavelength kinetics at 550 (blue) and 625 nm (red) as well as fit to the data (black line).

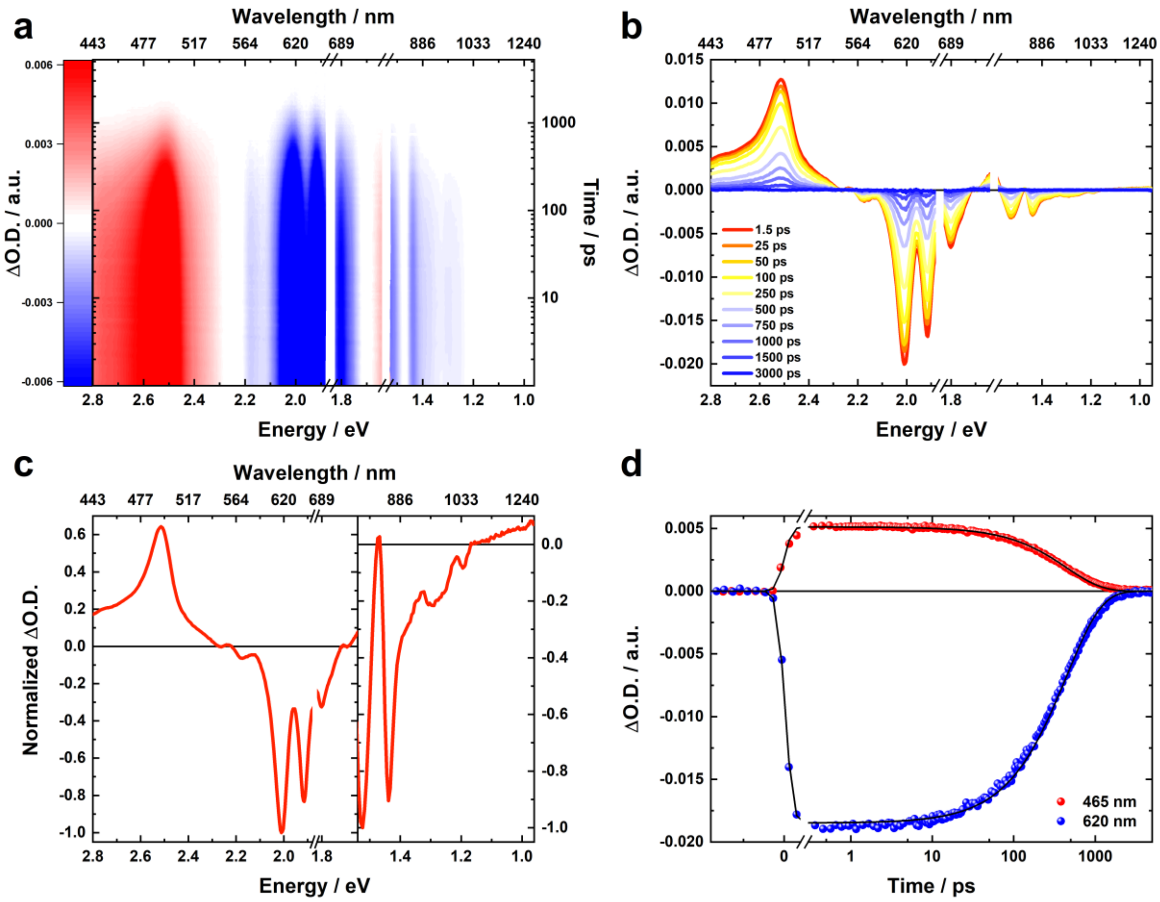

Figure S7: **a**, Zero point and chirp corrected fsTA heat map in the visible and near-infrared part of the optical spectrum of **2** obtained at 80 K upon photoexcitation at 670 nm and concentration of 5×10^{-6} M. **b**, Corresponding spectral slices illustrating the excited state dynamics. **c**, Evolution associated spectrum obtained by global analysis. **d**, Single wavelength kinetics at 465 (red) and 620 nm (blue) as well as fit to the data (black line).

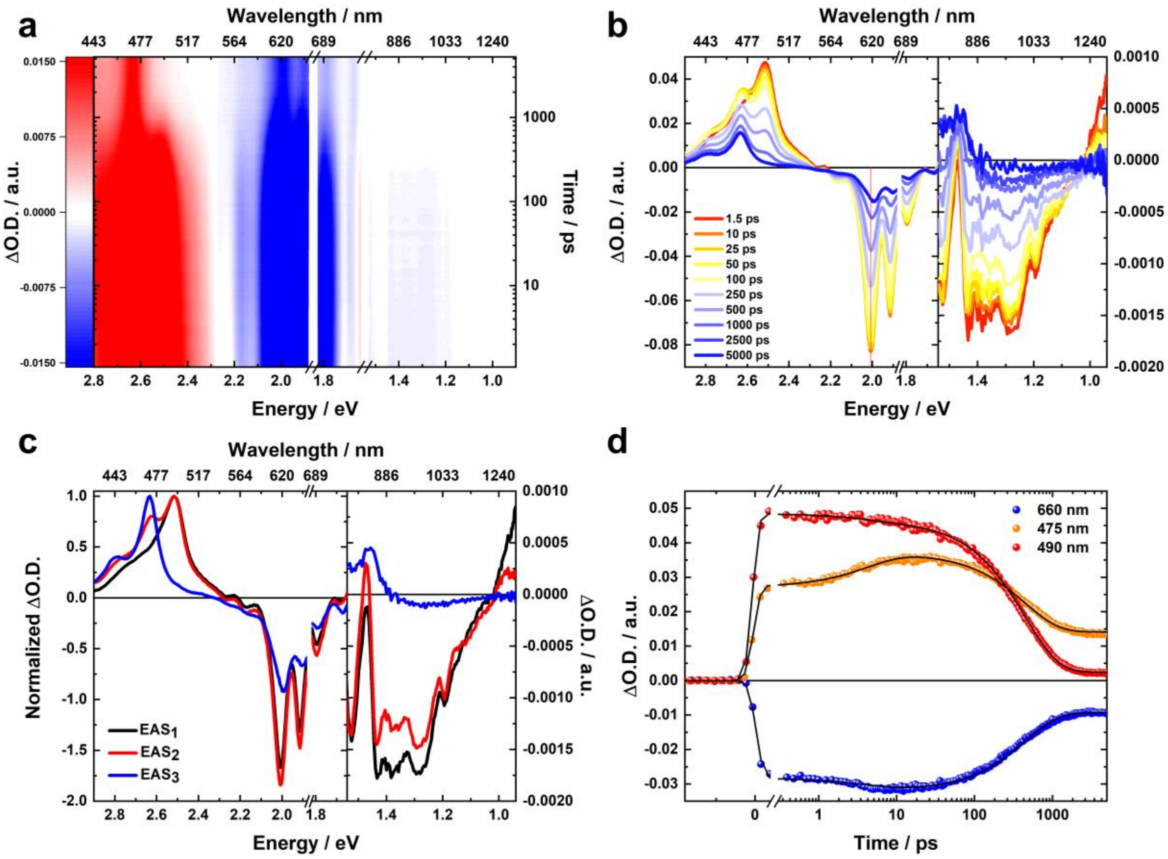

Figure S8: **a**, Zero point and chirp corrected fsTA heat map in the visible and near-infrared part of the optical spectrum of **2** obtained at 80 K upon photoexcitation at 670 nm and concentration of 1×10^{-3} M. **b**, Corresponding spectral slices illustrating the excited state dynamics. **c**, Evolution associated spectrum obtained by global analysis. **d**, Single wavelength kinetics at 475 (orange), 490 (red) and 660 nm (blue) as well as fit to the data (black line).

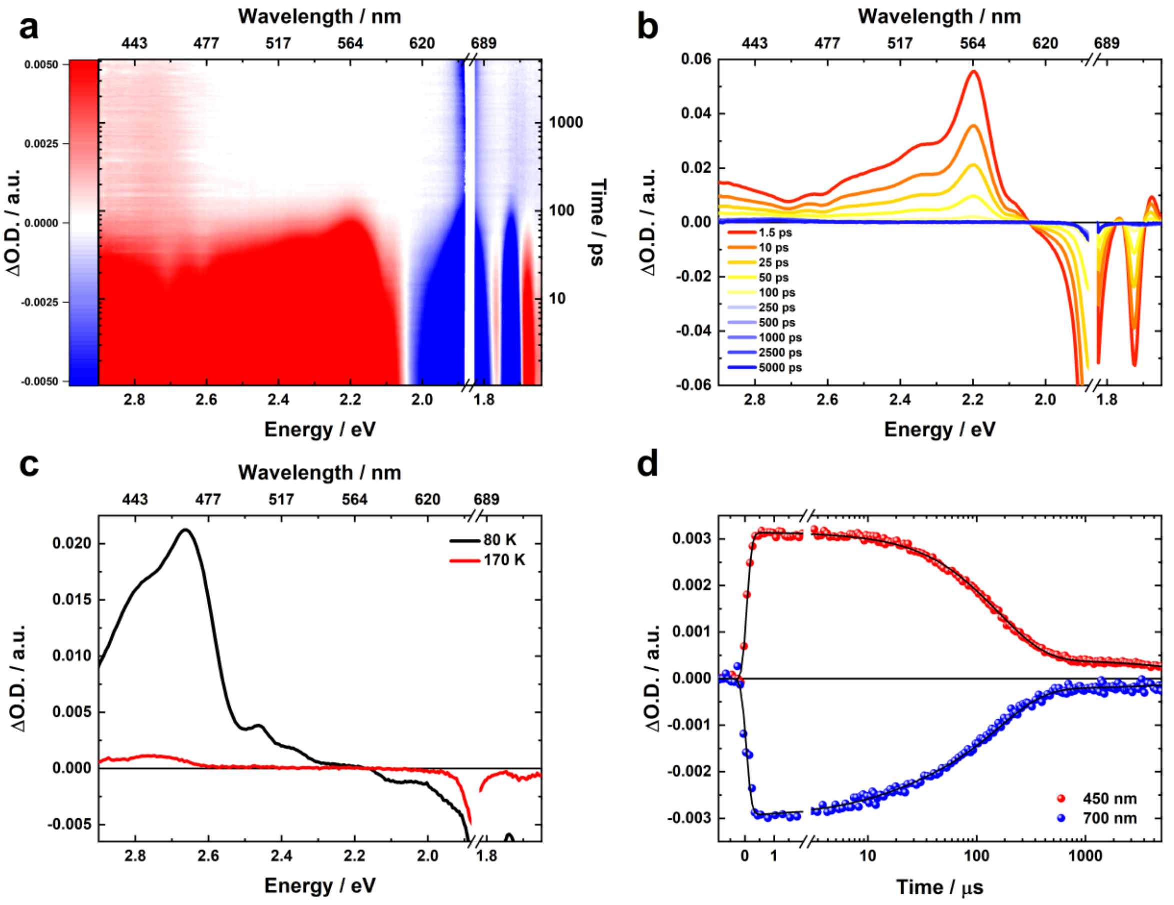

Figure S9: **a**, Zero point and chirp corrected fsTA heat map in the visible and near-infrared part of the optical spectrum of **3** obtained at 80 K upon photoexcitation at 670 nm and concentration of 1×10^{-3} M. **b**, Corresponding spectral slices illustrating the excited state dynamics. **c**, Spectral slices at a time delay of 1000 ps obtained at 80 (black) and 170 K (red). **d**, Single wavelength kinetics at 450 (red) and 700 nm (blue) as well as fit to the data (black line).

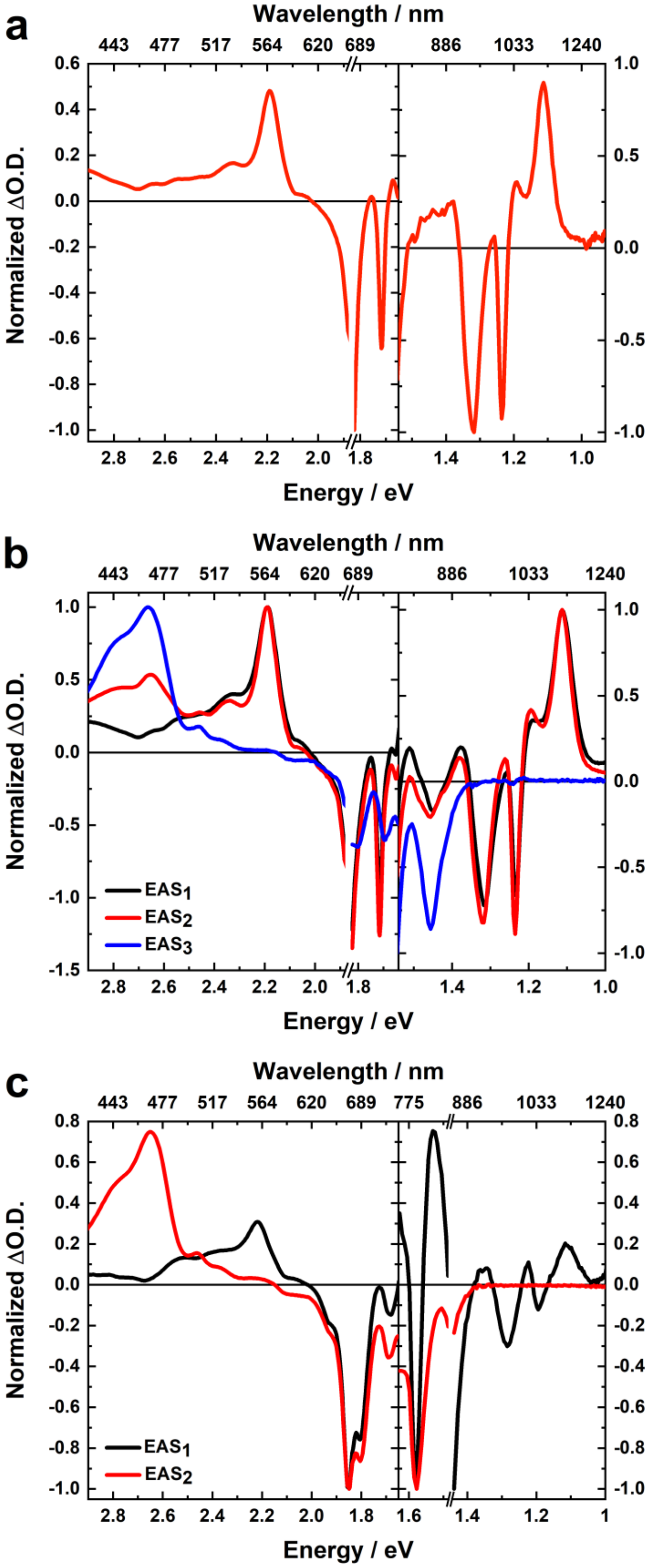

Figure S10: **a**, Evolution associated spectra of **3** obtained by global analysis at 80 K upon photoexcitation at 670 nm and concentration of 5×10^{-6} M. **b**, Evolution associated spectra of **3** obtained by global analysis at 80 K upon photoexcitation at 670 nm and concentration of 1×10^{-3} M. **c**, Evolution associated spectra of **3** obtained by global analysis at 80 K upon photoexcitation at 850 nm and concentration of 1×10^{-3} M.

Section S4. Nanosecond Transient Absorption Spectroscopy

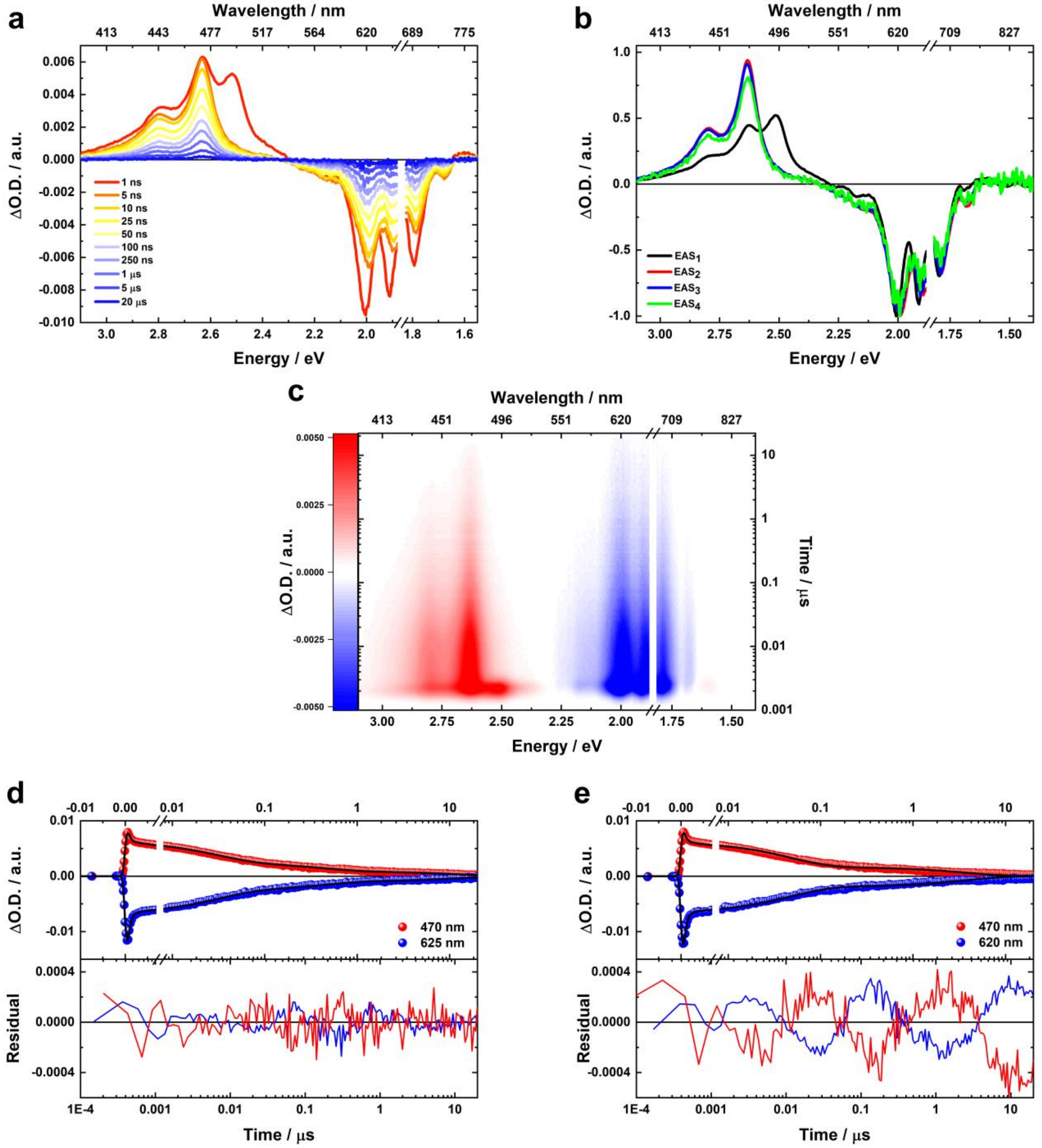

Figure S11: **a**, nsTA spectral slices in the visible part of the optical spectrum of **2** obtained at 80 K upon photoexcitation at 670 nm and concentration of 1×10^{-3} M. **b**, Evolution associated spectrum obtained by global analysis. **c**, Corresponding nsTA heat map. **d**, Single wavelength kinetics at 470 (red) and 625 nm (blue) as well as fit to the data (black line) obtained by global analysis with four kinetic components. **e**, Single wavelength kinetics at 470 (red) and 625 nm (blue) as well as fit to the data (black line) obtained by global analysis with three kinetic components. Comparison of the residuals in **d**,**e** reveals the necessity of four kinetic parameters to describe the deactivation kinetics of **2** in the ns time domain.

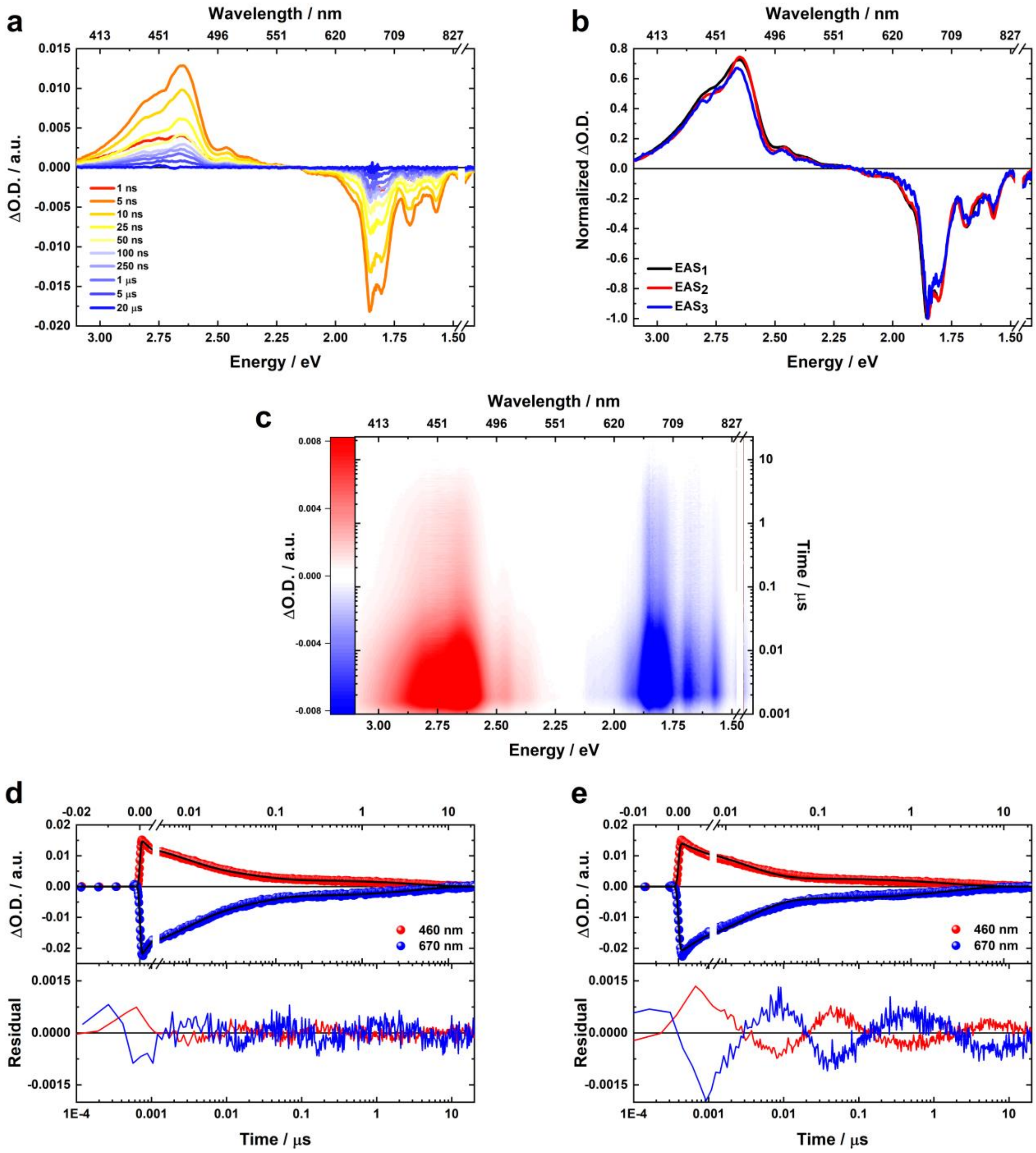

Figure S12: **a,** nsTA spectral slices in the visible part of the optical spectrum of **3** obtained at 80 K upon photoexcitation at 850 nm and concentration of 1×10^{-3} M. **b**, Evolution associated spectrum obtained by global analysis. **c**, Corresponding nsTA heat map. **d**, Single wavelength kinetics at 460 (red) and 670 nm (blue) as well as fit to the data (black line) obtained by global analysis with three kinetic components. **e**, Single wavelength kinetics at 460 (red) and 670 nm (blue) as well as fit to the data (black line) obtained by global analysis with two kinetic components. Comparison of the residuals in **d**,**e** reveals the necessity of three kinetic parameters to describe the triplet deactivation of **3** in the ns time domain.

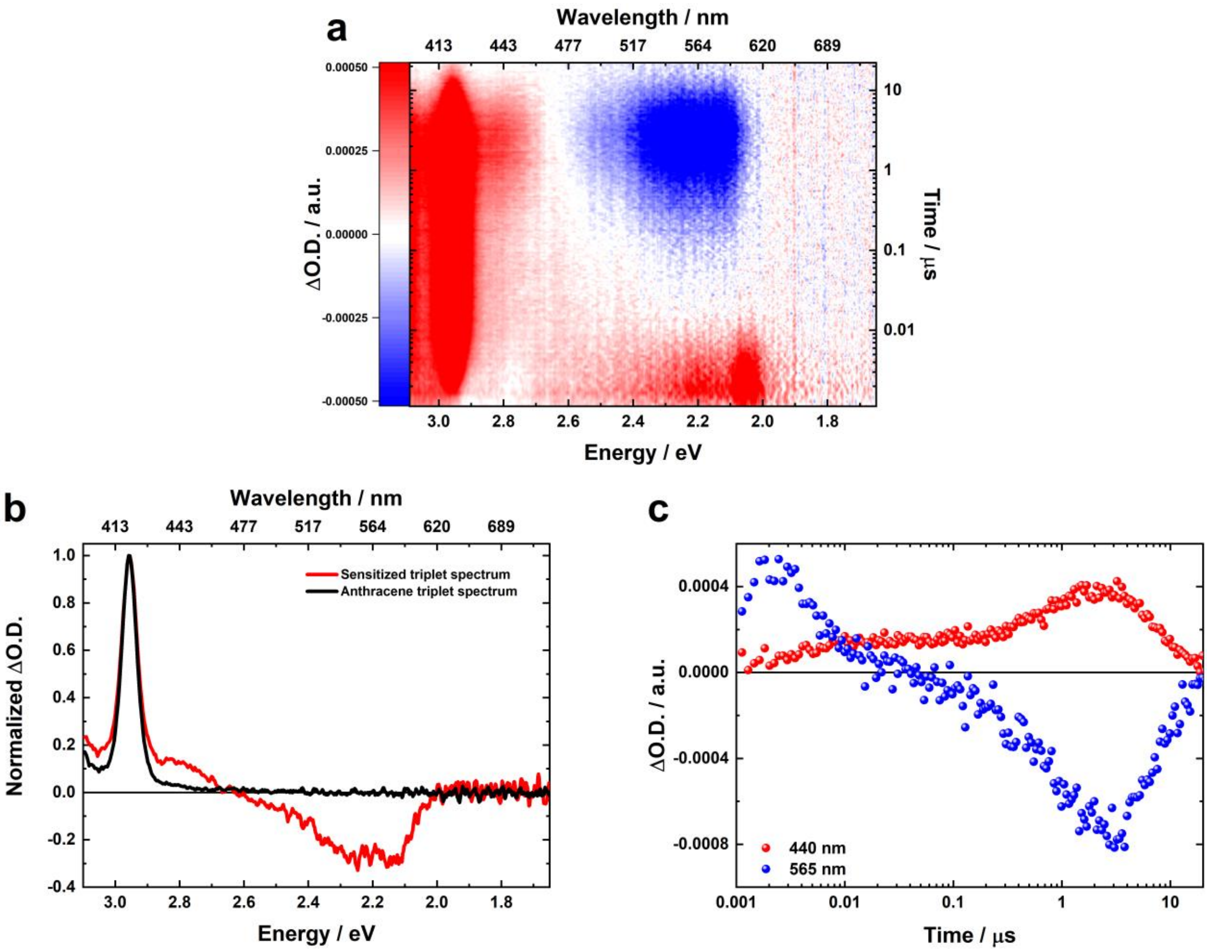

Figure S13: **a**, nsTA heat map in the visible part of the optical spectrum of the triplet-triplet sensitization experiment of **1** (3×10^{-5} M) with anthracene (8×10^{-5} M) obtained at room temperature upon photoexcitation at 387 nm. **b**, Normalized TA spectra of anthracene (black) and sensitized triplet (red) obtained at a time delay of 2.2 µs. **c**, Corresponding single wavelength kinetics at 440 (red) and 670 nm (blue).

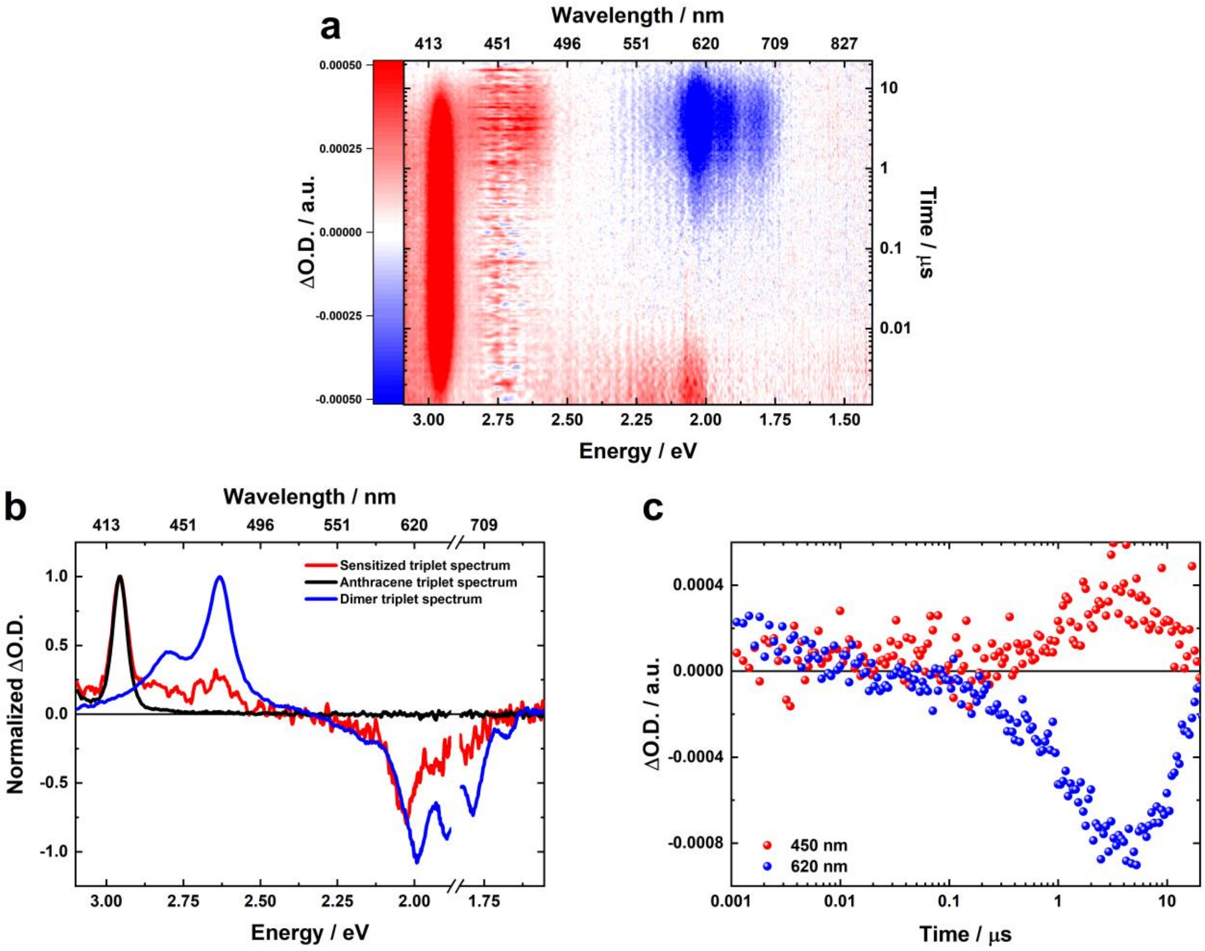

Figure S14: **a**, nsTA heat map in the visible part of the optical spectrum of the triplet-triplet sensitization experiment of **2** (3×10^{-5} M) with anthracene (8×10^{-5} M) obtained at room temperature upon photoexcitation at 387 nm. **b**, Normalized TA spectra of anthracene (black) and sensitized triplet (red) obtained at a time delay of 2.2 µs. **c,** Corresponding single wavelength kinetics at 450 (red) and 620 nm (blue).

Section S5. Computational Details – General

The molecular geometries were optimized using ORCA 4.0.1[2] with the B3LYP hybrid functional[3-6] in conjunction with Grimme's 3rd generation atom-pairwise dispersion correction[7] including Becke–Johnson damping[8] (D3BJ) starting from the structural parameters as obtained from the solid state structures of the monomers.[1] For the optimization, the singlet multiplicity was chosen, which led to better agreement with the solid state structures than the triplet multiplicity.[9] For the dimers, the structural parameters of the singlet, triplet and quintet multiplicities were optimized. In all geometry optimization, the def2-SVP basis set,[10] the resolution of identity and chain of spheres approximation[11,12] (RIJCOSX) and the related auxiliary basis sets[13] (def2/J) were used. Tighter than default convergence criteria (tightopt, tightscf) as well as finer than default grid values (grid7, finalgrid7, gridx6) were applied. Test calculations with the PBE0 functional led to comparable results.

The complete active-space self-consistent-field[14] (CASSCF) calculations were performed on truncated DFT-optimized structures (menthyl and Dipp groups were replaced by methyl groups; the position of the hydrogen atoms were optimized with restrained positions for all other atoms). The ZORA approximation[15-17] was employed along with the ZORA-def2-TZVPP basis sets[10,18] and the related auxiliary basis set[19] (SARC/J). Test calculations with OpenMolcas led to comparable results as obtained with ORCA. For the state-averaged sa-CASSCF calculations, various number of roots (quintet: 1–2; triplet 3–5; singlet 5–20) and active spaces (4e/4o; 8e/8o; 12e/12o) were calculated. The same weight was applied to all roots. Second-order perturbation theory NEVPT2 including the treatment of all the core electrons (nofrozencore) was applied to account for dynamic electron correlation.[20] Quasi-degenerate perturbation theory (QDPT) was used to calculate model pin-orbit coupling. Calculated molecular structures and molecular orbitals were visualized with Avogadro 1.1.1, IboView and CYLView 1.0b.[21-23]

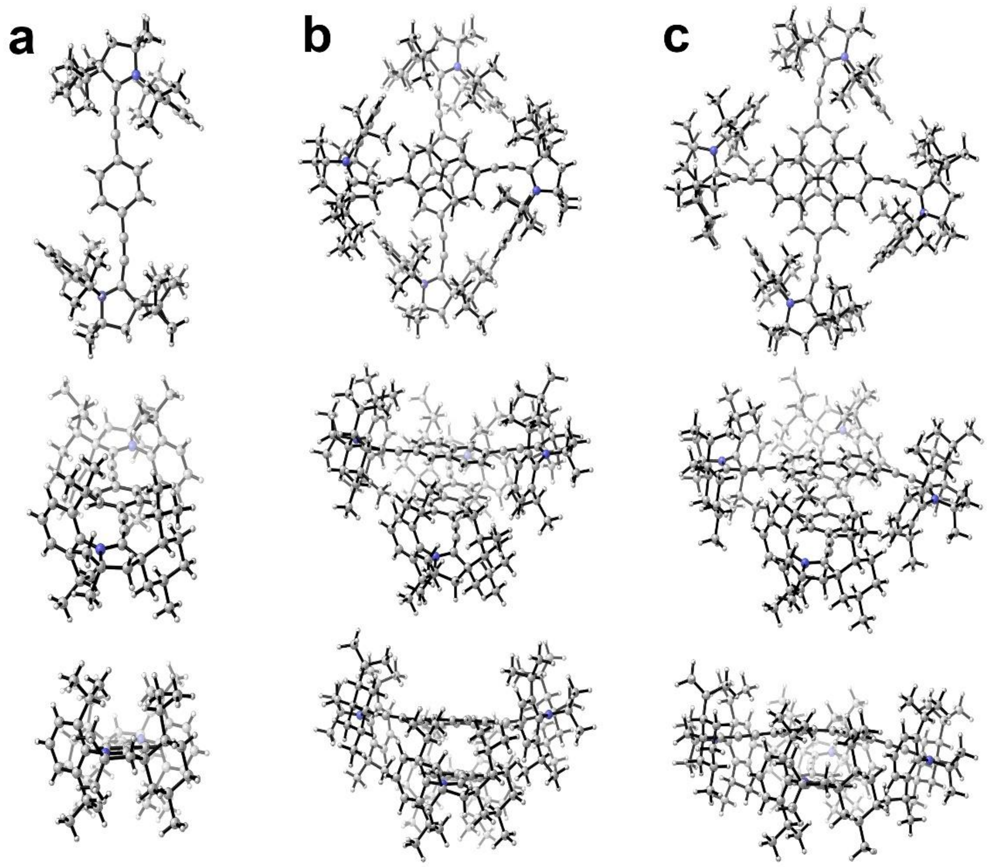

Figure S15: Optimized structures of **a**, phenyl bridged compound $\mathbf{1}^{\mathbf{monomer}}$, **b**, $\mathbf{2}^{\mathbf{dimer}}$ and **c**, $\mathbf{3}^{\mathbf{dimer}}$.

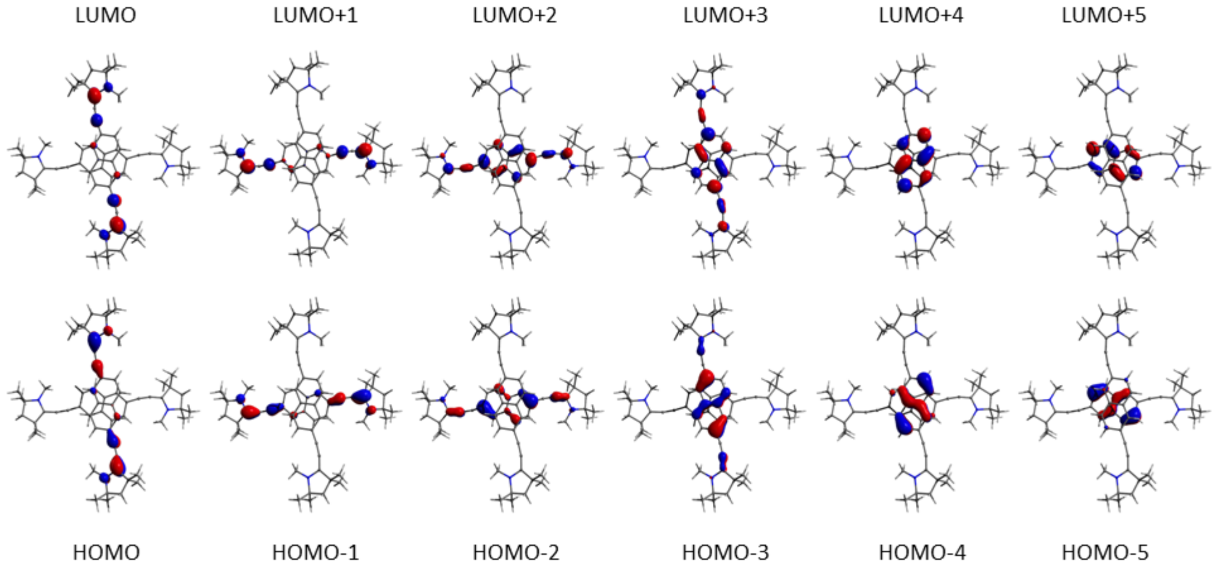

Figure S16: sa-CASSCF optimized orbitals of $\mathbf{2}^{\mathbf{dimer}}$ from state averaged calculation (singlet geometry, four singlet roots, four triplet roots and two quintet roots).

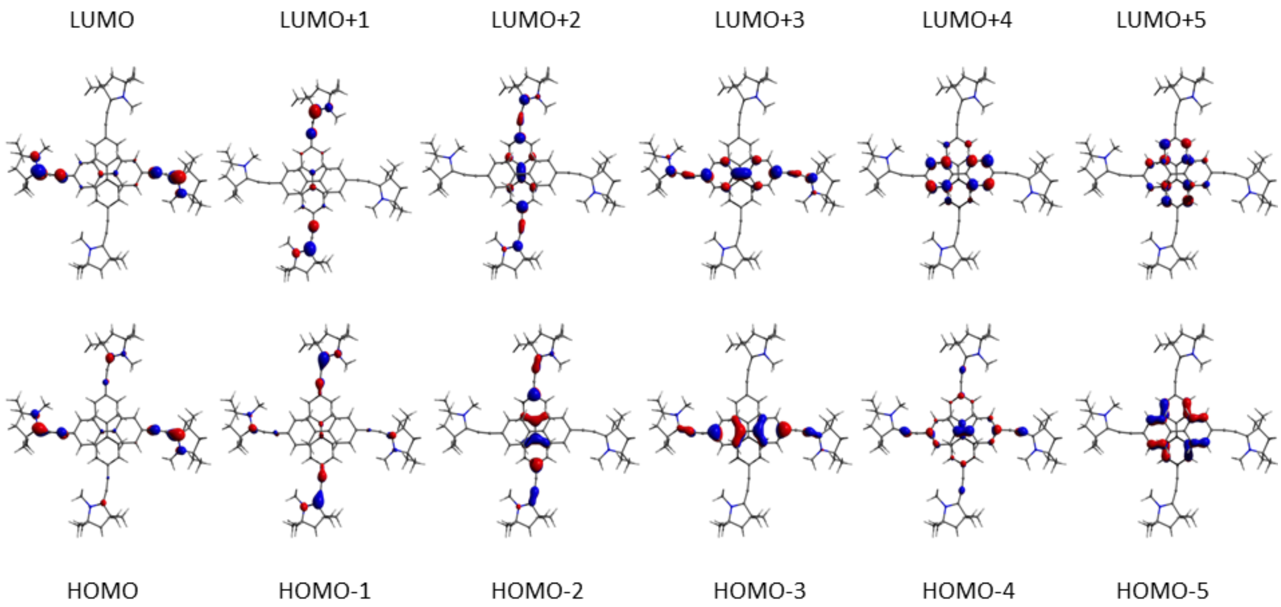

Figure S17: sa-CASSCF optimized orbitals **3**$^{\mathbf{dimer}}$ from state averaged calculation (singlet geometry, four singlet roots, four triplet roots and two quintet roots).

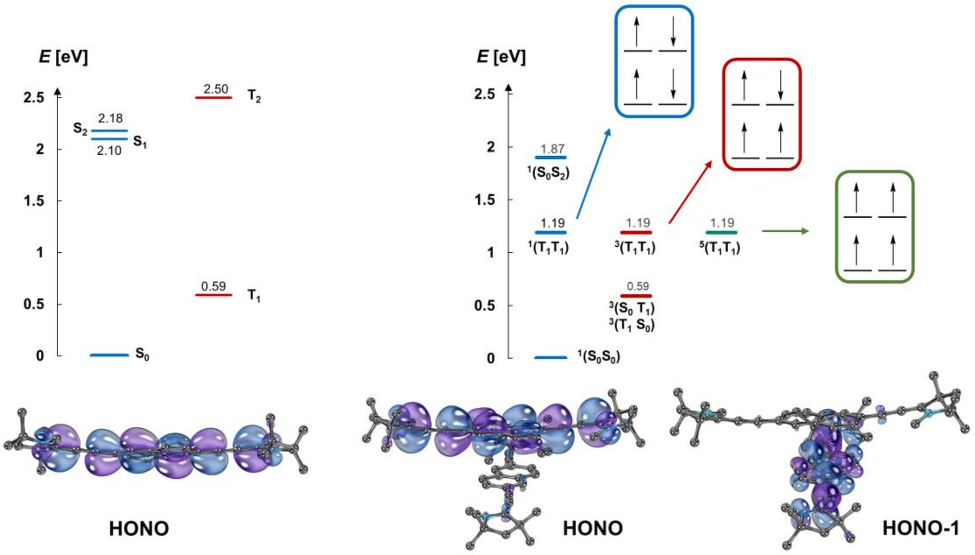

Figure S18: Electronic structure of **2**$^{\mathbf{monomer}}$ (left) and **2**$^{\mathbf{dimer}}$ as suggested by NEVPT2//sa-CASSCF(12,12) calculations.

Section S6. Configuration State Functions (CSFs)

2[dimer], sa-CASSCF(4,4) with eight singlet, four triplet and one quintet roots.

Q_1
1.00000 [0]: 1111

T_1
0.80775 [1]: 2101
0.19022 [11]: 0121

T_2
0.75160 [3]: 1210
0.20122 [9]: 1012
0.04498 [6]: 1111

T_3
0.99389 [6]: 1111

T_4
0.98974 [0]: 2110
0.00645 [2]: 2011
0.00264 [6]: 1111

S_0
0.60663 [13]: 0220
0.15815 [18]: 0022
0.13955 [0]: 2200
0.04064 [5]: 2002
0.03672 [16]: 0121
0.00760 [2]: 2101
0.00446 [4]: 2011
0.00312 [7]: 1201

S_1
0.60848 [0]: 2200
0.16335 [5]: 2002
0.14326 [13]: 0220
0.03840 [18]: 0022
0.03653 [2]: 2101
0.00857 [16]: 0121

S_2
0.74800 [6]: 1210
0.19817 [12]: 1012
0.04484 [9]: 1111
0.00356 [4]: 2011

S_3
0.66263 [2]: 2101
0.15257 [16]: 0121
0.09243 [0]: 2200
0.04603 [5]: 2002
0.02369 [13]: 0220
0.01002 [18]: 0022
0.00548 [4]: 2011
0.00289 [8]: 1120

S_4
0.98797 [7]: 1201
0.00353 [0]: 2200

S_5
0.99085 [9]: 1111
0.00369 [1]: 2110

S_6
0.98597 [1]: 2110
0.00661 [4]: 2011
0.00411 [9]: 1111

S_7
0.96891 [4]: 2011
0.00834 [8]: 1120
0.00714 [1]: 2110
0.00394 [0]: 2200
0.00278 [18]: 0022

3[dimer], sa-CASSCF(4,4) with eight singlet, four triplet and one quintet roots.

Q_1
1.00000 [0]: 1111

T_1
0.60825 [1]: 2101
0.33117 [11]: 0121
0.04810 [6]: 1111
0.00580 [8]: 1021
0.00580 [4]: 1201

T_2
0.46938 [3]: 1210
0.33174 [9]: 1012
0.18407 [6]: 1111
0.00868 [0]: 2110
0.00293 [12]: 0112

T_3
0.97266 [6]: 1111
0.01738 [2]: 2011
0.00965 [10]: 0211

T_4
0.90527 [0]: 2110
0.06945 [2]: 2011
0.01374 [5]: 1120
0.00785 [3]: 1210

S_0
0,26089 [13]: 0220
0,17411 [18]: 0022
0,10749 [6]: 1210
0,08996 [16]: 0121
0,07864 [0]: 2200
0,07201 [12]: 1012
0,05549 [7]: 1201
0,04503 [5]: 2002
0,04240 [9]: 1111
0,04049 [2]: 2101
0,01412 [11]: 1021
0,01022 [8]: 1120
0,00619 [10]: 1102

S_1
0,29336 [0]: 2200
0,20252 [5]: 2002
0,15566 [13]: 0220
0,11298 [2]: 2101

0,11270 [18]: 0022
0,06194 [16]: 0121
0,02273 [6]: 1210
0,01614 [12]: 1012
0,00915 [9]: 1111
0,00547 [8]: 1120
0,00337 [10]: 1102

S_2

0,64236 [7]: 1201
0,20840 [10]: 1102
0,03443 [14]: 0211
0,01627 [12]: 1012
0,01571 [5]: 2002
0,01551 [6]: 1210
0,01348 [17]: 0112
0,01318 [13]: 0220
0,01017 [2]: 2101
0,00798 [18]: 0022
0,00651 [15]: 0202
0,00638 [16]: 0121
0,00367 [9]: 1111
0,00262 [8]: 1120

S_3

0,95902 [9]: 1111
0,02607 [4]: 2011
0,01447 [14]: 0211

S_4

0,26545 [6]: 1210
0,18327 [12]: 1012
0,13360 [4]: 2011
0,11788 [9]: 1111
0,08334 [0]: 2200
0,06714 [5]: 2002
0,03703 [13]: 0220
0,03353 [2]: 2101
0,03035 [18]: 0022
0,01627 [16]: 0121
0,01365 [8]: 1120
0,00898 [1]: 2110
0,00413 [7]: 1201

S_5

0,75170 [4]: 2011
0,06440 [1]: 2110
0,05119 [6]: 1210
0,03906 [12]: 1012
0,03016 [9]: 1111
0,01781 [0]: 2200
0,01465 [5]: 2002
0,01197 [11]: 1021
0,00688 [13]: 0220
0,00431 [2]: 2101
0,00357 [18]: 0022
0,00292 [16]: 0121

S_6

0,90792 [1]: 2110
0,06560 [4]: 2011
0,01362 [8]: 1120
0,00683 [6]: 1210
0,00295 [9]: 1111

S_7

0,85151 [8]: 1120
0,05198 [11]: 1021
0,03100 [3]: 2020
0,01304 [12]: 1012
0,01174 [1]: 2110
0,01146 [6]: 1210
0,00854 [4]: 2011
0,00784 [9]: 1111
0,00395 [16]: 0121
0,00335 [5]: 2002

2$^{\mathbf{dimer}}$, sa-CASSCF(8,8) with eight singlet, three triplet and one quintet roots.

Q_1

0.88893 [0]: 22111100
0.00957 [238]: 11111111
0.00762 [34]: 21111200
0.00683 [10]: 22011110
0.00661 [7]: 22101101
0.00661 [128]: 12112100
0.00494 [28]: 21120110
0.00482 [16]: 21211010
0.00466 [27]: 21121001
0.00451 [19]: 21210101
0.00431 [114]: 12211001
0.00412 [115]: 12210110
0.00412 [123]: 12121010
0.00395 [126]: 12120101
0.00298 [67]: 21011210
0.00289 [51]: 21101201
0.00257 [161]: 12012110
0.00253 [95]: 20111120
0.00253 [145]: 12102101

T_1

0.26788 [0]: 22211000
0.17028 [11]: 22120100
0.13483 [1]: 22210100
0.13426 [10]: 22121000
0.05792 [49]: 22011200
0.03892 [24]: 22102100
0.03011 [46]: 22012100
0.02965 [27]: 22101200
0.01104 [367]: 12111101
0.01033 [119]: 21111110
0.00712 [120]: 21111101
0.00628 [366]: 12111110
0.00531 [77]: 21220010
0.00352 [325]: 12220001
0.00309 [80]: 21211100
0.00278 [16]: 22111010
0.00278 [78]: 21220001
0.00277 [465]: 11211011
0.00267 [324]: 12220010

S_2

0.21621 [10]: 22121000
0.20927 [1]: 22210100
0.18134 [11]: 22120100
0.09879 [0]: 22211000
0.04777 [27]: 22101200
0.04730 [46]: 22012100
0.04145 [24]: 22102100
0.02164 [49]: 22011200
0.01077 [120]: 21111101
0.01015 [366]: 12111110
0.00851 [119]: 21111110
0.00543 [367]: 12111101
0.00459 [78]: 21220001
0.00405 [324]: 12220010
0.00352 [106]: 21121100
0.00317 [325]: 12220001
0.00296 [19]: 22110110

T_3

0.21942 [14]: 22112000
0.21879 [4]: 22201100
0.21698 [18]: 22110200
0.21675 [40]: 22021100
0.01433 [15]: 22111100
0.00477 [107]: 21121010
0.00475 [84]: 21210110
0.00467 [82]: 21211001
0.00458 [111]: 21120101
0.00422 [328]: 12211010
0.00419 [357]: 12120110
0.00410 [355]: 12121001
0.00408 [332]: 12210101
0.00395 [115]: 21112100
0.00349 [28]: 22101110
0.00333 [51]: 22011101
0.00325 [365]: 12111200

S_0

0.56184 [0]: 22220000
0.12131 [20]: 22111100
0.03365 [5]: 22202000
0.03133 [49]: 22020200
0.03062 [45]: 22022000
0.02974 [9]: 22200200
0.02791 [71]: 22002200
0.01208 [125]: 21120110
0.01179 [100]: 21210101
0.01086 [389]: 12121010
0.01079 [364]: 12211001
0.00960 [96]: 21211010
0.00881 [123]: 21121001
0.00843 [393]: 12120101
0.00814 [366]: 12210110
0.00716 [91]: 21220100
0.00648 [357]: 12221000
0.00575 [17]: 22120010
0.00561 [4]: 22210001
0.00504 [491]: 11220011
0.00326 [553]: 11111111

S_1

0.22058 [20]: 22111100
0.16221 [5]: 22202000
0.16114 [45]: 22022000
0.16081 [9]: 22200200
0.15899 [49]: 22020200
0.00566 [19]: 22112000
0.00553 [23]: 22110200
0.00507 [6]: 22201100
0.00497 [46]: 22021100
0.00485 [125]: 21120110
0.00470 [96]: 21211010
0.00458 [100]: 21210101
0.00454 [123]: 21121001
0.00423 [364]: 12211001
0.00418 [389]: 12121010
0.00415 [366]: 12210110
0.00404 [393]: 12120101
0.00295 [165]: 21022100
0.00288 [104]: 21202100
0.00276 [147]: 21102110
0.00275 [38]: 22100210
0.00262 [65]: 22010201
0.00259 [183]: 21012101

S_2

0.27932 [1]: 22211000
0.24264 [16]: 22120100
0.07019 [10]: 22200110
0.06239 [50]: 22020110
0.04783 [15]: 22121000
0.03531 [365]: 12210200
0.03282 [361]: 12212000
0.01728 [21]: 22111010
0.01390 [391]: 12120200
0.01358 [387]: 12122000
0.01216 [51]: 22020101
0.01105 [11]: 22200101
0.01036 [388]: 12121100
0.00955 [398]: 12112010
0.00841 [436]: 12021110
0.00774 [2]: 22210100
0.00598 [8]: 22201001
0.00558 [24]: 22110110
0.00520 [22]: 22111001
0.00494 [406]: 12110210
0.00487 [362]: 12211100
0.00462 [48]: 22021001
0.00394 [134]: 21111110
0.00367 [375]: 12201110
0.00357 [120]: 21122000
0.00329 [36]: 22101011
0.00292 [67]: 22010111
0.00288 [402]: 12111101
0.00270 [121]: 21121100

S_3

0.26027 [2]: 22210100

0.22883 [15]: 22121000
0.07622 [51]: 22020101
0.07265 [11]: 22200101
0.04594 [391]: 12120200
0.04231 [387]: 12122000
0.03555 [1]: 22211000
0.03433 [16]: 22120100
0.02305 [22]: 22111001
0.01479 [362]: 12211100
0.01170 [399]: 12112001
0.01130 [376]: 12201101
0.01067 [365]: 12210200
0.01036 [10]: 22200110
0.01023 [361]: 12212000
0.00912 [50]: 22020110
0.00674 [407]: 12110201
0.00596 [437]: 12021101
0.00371 [135]: 21111101
0.00325 [21]: 22111010
0.00315 [401]: 12111110
0.00303 [388]: 12121100
0.00292 [41]: 22100111
0.00282 [25]: 22110101
0.00277 [108]: 21201110
0.00275 [131]: 21112010
0.00270 [62]: 22011011

S_4

0.07160 [71]: 22002200
0.06682 [4]: 22210001
0.06610 [17]: 22120010
0.06457 [20]: 22111100
0.06305 [91]: 21220100
0.05643 [357]: 12221000
0.05562 [3]: 22210010
0.05019 [90]: 21221000
0.04263 [19]: 22112000
0.04042 [6]: 22201100
0.02863 [18]: 22120001
0.02700 [5]: 22202000
0.02284 [358]: 12220100
0.02246 [46]: 22021100
0.02089 [23]: 22110200
0.01611 [9]: 22200200
0.01450 [45]: 22022000
0.01229 [0]: 22220000
0.01088 [49]: 22020200
0.00714 [33]: 22101110
0.00707 [60]: 22011101
0.00672 [100]: 21210101
0.00668 [130]: 21112100
0.00659 [125]: 21120110
0.00624 [364]: 12211001
0.00612 [389]: 12121010
0.00610 [400]: 12111200
0.00581 [27]: 22110011
0.00559 [59]: 22011110
0.00512 [484]: 11221100

0.00496 [168]: 21021200
0.00494 [64]: 22010210
0.00484 [133]: 21111200
0.00476 [142]: 21110111
0.00417 [404]: 12111011
0.00411 [532]: 11121110
0.00396 [498]: 11211101
0.00395 [56]: 22012010
0.00353 [107]: 21201200
0.00337 [96]: 21211010
0.00325 [39]: 22100201
0.00291 [432]: 12022100
0.00284 [34]: 22101101
0.00284 [148]: 21102101
0.00277 [38]: 22100210
0.00274 [31]: 22102001
0.00271 [65]: 22010201
0.00261 [12]: 22200020
0.00256 [152]: 21101210
0.00255 [182]: 21012110

S_5

0.07332 [18]: 22120001
0.05602 [358]: 12220100
0.05461 [46]: 22021100
0.05422 [3]: 22210010
0.05146 [90]: 21221000
0.05072 [23]: 22110200
0.04744 [71]: 22002200
0.04613 [17]: 22120010
0.04527 [91]: 21220100
0.04409 [19]: 22112000
0.04377 [20]: 22111100
0.04135 [6]: 22201100
0.04061 [4]: 22210001
0.03353 [357]: 12221000
0.02181 [49]: 22020200
0.01166 [33]: 22101110
0.01108 [45]: 22022000
0.01101 [130]: 21112100
0.01076 [60]: 22011101
0.01057 [9]: 22200200
0.00903 [400]: 12111200
0.00760 [31]: 22102001
0.00757 [0]: 22220000
0.00635 [56]: 22012010
0.00594 [107]: 21201200
0.00588 [371]: 12202100
0.00487 [39]: 22100201
0.00462 [168]: 21021200
0.00460 [125]: 21120110
0.00437 [64]: 22010210
0.00436 [100]: 21210101
0.00429 [182]: 21012110
0.00425 [152]: 21101210
0.00423 [432]: 12022100
0.00412 [188]: 21011201
0.00393 [34]: 22101101

0.00389 [389]: 12121010
0.00385 [148]: 21102101
0.00375 [454]: 12011210
0.00372 [5]: 22202000
0.00371 [27]: 22110011
0.00361 [364]: 12211001
0.00344 [414]: 12102110
0.00334 [137]: 21111011
0.00334 [450]: 12012101
0.00333 [484]: 11221100
0.00332 [126]: 21120101
0.00330 [420]: 12101201
0.00286 [404]: 12111011
0.00284 [142]: 21110111
0.00283 [397]: 12112100
0.00281 [497]: 11211110
0.00265 [498]: 11211101
0.00252 [532]: 11121110

S_6

0.10613 [7]: 22201010
0.10520 [47]: 22021010
0.08712 [94]: 21212000
0.08543 [15]: 22121000
0.08147 [98]: 21210200
0.07165 [2]: 22210100
0.04966 [24]: 22110110
0.04276 [121]: 21121100
0.02668 [22]: 22111001
0.02356 [169]: 21021110
0.02327 [139]: 21110210
0.02313 [131]: 21112010
0.02291 [362]: 12211100
0.02283 [11]: 22200101
0.02171 [108]: 21201110
0.02107 [51]: 22020101
0.02012 [387]: 12122000
0.01828 [391]: 12120200
0.01523 [407]: 12110201
0.01508 [437]: 12021101
0.00835 [399]: 12112001
0.00787 [376]: 12201101
0.00526 [16]: 22120100
0.00480 [1]: 22211000
0.00440 [21]: 22111010
0.00263 [95]: 21211100

S_7

0.10060 [8]: 22201001
0.09930 [48]: 22021001
0.08756 [120]: 21122000
0.08278 [124]: 21120200
0.05224 [25]: 22110101
0.04649 [95]: 21211100
0.04434 [1]: 22211000
0.04118 [16]: 22120100
0.03727 [50]: 22020110
0.03560 [10]: 22200110
0.03469 [21]: 22111010
0.03188 [361]: 12212000
0.03036 [365]: 12210200
0.02893 [388]: 12121100
0.02578 [132]: 21112001
0.02407 [109]: 21201101
0.02376 [170]: 21021101
0.02369 [140]: 21110201
0.01890 [406]: 12110210
0.01857 [375]: 12201110
0.01171 [398]: 12112010
0.01119 [436]: 12021110
0.00366 [22]: 22111001
0.00300 [2]: 22210100
0.00292 [121]: 21121100

3^{dimer}, sa-CASSCF(8,8) with eight singlet, three triplet and one quintet roots.

Q_1

0.90953 [0]: 22111100
0.00770 [10]: 22011110
0.00531 [7]: 22101101
0.00370 [34]: 21111200
0.00367 [128]: 12112100
0.00326 [26]: 21121010
0.00320 [125]: 12120110
0.00318 [31]: 21112100
0.00317 [131]: 12111200
0.00290 [116]: 12210101
0.00290 [17]: 21211001
0.00260 [380]: 02111111
0.00258 [96]: 20111111

T_1

0.32772 [0]: 22211000
0.18849 [10]: 22121000
0.11073 [11]: 22120100
0.09045 [49]: 22011200
0.06832 [27]: 22101200
0.04248 [1]: 22210100
0.04068 [24]: 22102100
0.01566 [119]: 21111110
0.01392 [120]: 21111101
0.01179 [46]: 22012100
0.00516 [324]: 12220010
0.00438 [366]: 12111110
0.00367 [325]: 12220001
0.00349 [16]: 22111010
0.00302 [93]: 21201110
0.00296 [80]: 21211100

T_2

0.32211 [1]: 22210100
0.19062 [11]: 22120100
0.11196 [10]: 22121000
0.09074 [46]: 22012100
0.07066 [24]: 22102100
0.04203 [0]: 22211000
0.04097 [27]: 22101200

0.01509 [366]: 12111110
0.01433 [367]: 12111101
0.01179 [49]: 22011200
0.00535 [77]: 21220010
0.00439 [119]: 21111110
0.00358 [78]: 21220001
0.00342 [19]: 22110110
0.00294 [340]: 12201110
0.00285 [327]: 12211100

T_3

0.25004 [4]: 22201100
0.20380 [40]: 22021100
0.15979 [14]: 22112000
0.15820 [18]: 22110200
0.13112 [15]: 22111100
0.00379 [81]: 21211010
0.00371 [28]: 22101110
0.00366 [331]: 12210110
0.00321 [118]: 21111200
0.00316 [362]: 12112100
0.00262 [51]: 22011101
0.00256 [358]: 12120101
0.00252 [108]: 21121001

S_0

0.48934 [0]: 22220000
0.11214 [20]: 22111100
0.05042 [71]: 22002200
0.04685 [5]: 22202000
0.04439 [9]: 22200200
0.03421 [49]: 22020200
0.03355 [45]: 22022000
0.02365 [19]: 22112000
0.02257 [23]: 22110200
0.00887 [99]: 21210110
0.00877 [363]: 12211010
0.00672 [390]: 12121001
0.00666 [126]: 21120101
0.00589 [125]: 21120110
0.00553 [389]: 12121010
0.00547 [364]: 12211001
0.00516 [100]: 21210101
0.00493 [122]: 21121010
0.00492 [392]: 12120110
0.00463 [97]: 21211001
0.00457 [367]: 12210101
0.00351 [17]: 22120010
0.00342 [4]: 22210001
0.00296 [182]: 21012110
0.00287 [454]: 12011210
0.00270 [148]: 21102101
0.00265 [420]: 12101201

S_1

0.22203 [20]: 22111100
0.13273 [5]: 22202000
0.13168 [9]: 22200200
0.10903 [6]: 22201100
0.10838 [45]: 22022000
0.10714 [49]: 22020200
0.08884 [46]: 22021100
0.00345 [122]: 21121010
0.00332 [392]: 12120110
0.00315 [15]: 22121000
0.00282 [152]: 21101210
0.00280 [367]: 12210101
0.00280 [97]: 21211001
0.00274 [414]: 12102110
0.00262 [168]: 21021200
0.00259 [432]: 12022100

S_2

0.14365 [3]: 22210010
0.08279 [357]: 12221000
0.07909 [6]: 22201100
0.07616 [19]: 22112000
0.06896 [23]: 22110200
0.06773 [91]: 21220100
0.06757 [18]: 22120001
0.03099 [46]: 22021100
0.02110 [33]: 22101110
0.01862 [5]: 22202000
0.01500 [64]: 22010210
0.01428 [9]: 22200200
0.01420 [56]: 22012010
0.01295 [374]: 12201200
0.01194 [104]: 21202100
0.01166 [60]: 22011101
0.01112 [400]: 12111200
0.00940 [147]: 21102110
0.00912 [419]: 12101210
0.00912 [130]: 21112100
0.00905 [397]: 12112100
0.00892 [39]: 22100201
0.00851 [133]: 21111200
0.00817 [31]: 22102001
0.00752 [45]: 22022000
0.00686 [358]: 12220100
0.00633 [435]: 12021200
0.00624 [389]: 12121010
0.00565 [455]: 12011201
0.00549 [165]: 21022100
0.00546 [183]: 21012101
0.00534 [49]: 22020200
0.00479 [125]: 21120110
0.00478 [38]: 22100210
0.00458 [182]: 21012110
0.00449 [454]: 12011210
0.00442 [142]: 21110111
0.00397 [449]: 12012110
0.00395 [187]: 21011210
0.00392 [30]: 22102010
0.00390 [497]: 11211110
0.00384 [420]: 12101201
0.00366 [148]: 21102101
0.00350 [363]: 12211010
0.00347 [404]: 12111011

0.00329 [90]: 21221000
0.00328 [415]: 12102101
0.00322 [153]: 21101201
0.00309 [364]: 12211001
0.00291 [65]: 22010201
0.00279 [533]: 11121101
0.00277 [366]: 12210110
0.00265 [26]: 22110020
0.00257 [99]: 21210110
0.00253 [96]: 21211010

S_3

0.14176 [71]: 22002200
0.11816 [17]: 22120010
0.10010 [4]: 22210001
0.05775 [20]: 22111100
0.05709 [91]: 21220100
0.04509 [90]: 21221000
0.04341 [357]: 12221000
0.04134 [358]: 12220100
0.03406 [0]: 22220000
0.02343 [49]: 22020200
0.01915 [45]: 22022000
0.01861 [9]: 22200200
0.01631 [5]: 22202000
0.01580 [23]: 22110200
0.01038 [130]: 21112100
0.01011 [59]: 22011110
0.00830 [19]: 22112000
0.00826 [30]: 22102010
0.00785 [400]: 12111200
0.00746 [100]: 21210101
0.00738 [125]: 21120110
0.00713 [34]: 22101101
0.00698 [498]: 11211101
0.00693 [364]: 12211001
0.00662 [27]: 22110011
0.00660 [38]: 22100210
0.00641 [532]: 11121110
0.00632 [97]: 21211001
0.00613 [367]: 12210101
0.00604 [389]: 12121010
0.00600 [122]: 21121010
0.00571 [99]: 21210110
0.00556 [392]: 12120110
0.00528 [57]: 22012001
0.00477 [2]: 22210100
0.00463 [65]: 22010201
0.00458 [363]: 12211010
0.00433 [404]: 12111011
0.00395 [126]: 21120101
0.00394 [553]: 11111111
0.00377 [104]: 21202100
0.00362 [390]: 12121001
0.00341 [142]: 21110111
0.00341 [409]: 12110111
0.00325 [137]: 21111011
0.00292 [56]: 22012010
0.00283 [12]: 22200020
0.00282 [220]: 20220200
0.00279 [484]: 11221100
0.00274 [165]: 21022100
0.00269 [107]: 21201200

S_4

0.12997 [1]: 22211000
0.11058 [7]: 22201010
0.08089 [15]: 22121000
0.07729 [47]: 22021010
0.05444 [94]: 21212000
0.05336 [98]: 21210200
0.05126 [95]: 21211100
0.04416 [21]: 22111010
0.03502 [2]: 22210100
0.03463 [10]: 22200110
0.03067 [121]: 21121100
0.02586 [16]: 22120100
0.02403 [50]: 22020110
0.02142 [169]: 21021110
0.02090 [108]: 21201110
0.01845 [131]: 21112010
0.01733 [139]: 21110210
0.01640 [134]: 21111110
0.01270 [24]: 22110110
0.01108 [22]: 22111001
0.00742 [170]: 21021101
0.00571 [135]: 21111101
0.00565 [48]: 22021001
0.00531 [140]: 21110201
0.00437 [8]: 22201001
0.00418 [132]: 21112001
0.00365 [362]: 12211100
0.00343 [25]: 22110101
0.00308 [120]: 21122000
0.00299 [365]: 12210200
0.00274 [361]: 12212000
0.00274 [109]: 21201101

S_5

0.11716 [2]: 22210100
0.10846 [10]: 22200110
0.08089 [16]: 22120100
0.07568 [50]: 22020110
0.05438 [361]: 12212000
0.05394 [365]: 12210200
0.05213 [362]: 12211100
0.04304 [24]: 22110110
0.03663 [1]: 22211000
0.03409 [7]: 22201010
0.03223 [388]: 12121100
0.02375 [47]: 22021010
0.02329 [15]: 22121000
0.02149 [436]: 12021110
0.02140 [375]: 12201110
0.01827 [406]: 12110210
0.01763 [398]: 12112010
0.01641 [401]: 12111110

0.01202 [25]: 22110101
0.01200 [21]: 22111010
0.00817 [437]: 12021101
0.00720 [51]: 22020101
0.00636 [11]: 22200101
0.00613 [402]: 12111101
0.00599 [399]: 12112001
0.00472 [407]: 12110201
0.00404 [22]: 22111001
0.00379 [95]: 21211100
0.00338 [391]: 12120200
0.00330 [376]: 12201101
0.00307 [94]: 21212000
0.00289 [98]: 21210200
0.00273 [387]: 12122000
0.00267 [4]: 22210001

S_6

0.20740 [1]: 22211000
0.11312 [8]: 22201001
0.06971 [15]: 22121000
0.06438 [48]: 22021001
0.05153 [121]: 21121100
0.04581 [124]: 21120200
0.04324 [120]: 21122000
0.04216 [2]: 22210100
0.03573 [95]: 21211100
0.02774 [16]: 22120100
0.02691 [109]: 21201101
0.02679 [22]: 22111001
0.02200 [11]: 22200101
0.01424 [170]: 21021101
0.01268 [51]: 22020101
0.01256 [135]: 21111101
0.01185 [25]: 22110101
0.01177 [108]: 21201110
0.01162 [132]: 21112001
0.01103 [140]: 21110201
0.01072 [387]: 12122000
0.00772 [21]: 22111010
0.00488 [134]: 21111110
0.00428 [169]: 21021110
0.00411 [362]: 12211100
0.00406 [139]: 21110210
0.00392 [391]: 12120200
0.00324 [376]: 12201101
0.00288 [47]: 22021010
0.00282 [98]: 21210200
0.00263 [131]: 21112010
0.00253 [36]: 22101011

S_7

0.22316 [2]: 22210100
0.10937 [11]: 22200101
0.06026 [16]: 22120100
0.06025 [51]: 22020101
0.04928 [388]: 12121100
0.04569 [1]: 22211000
0.04456 [387]: 12122000
0.04058 [391]: 12120200
0.03451 [362]: 12211100
0.02730 [376]: 12201101
0.02600 [25]: 22110101
0.02569 [15]: 22121000
0.02238 [8]: 22201001
0.01420 [437]: 12021101
0.01241 [402]: 12111101
0.01218 [48]: 22021001
0.01127 [124]: 21120200
0.01103 [22]: 22111001
0.01099 [407]: 12110201
0.01074 [399]: 12112001
0.01060 [375]: 12201110
0.00664 [24]: 22110110
0.00421 [401]: 12111110
0.00409 [95]: 21211100
0.00387 [17]: 22120010
0.00384 [436]: 12021110
0.00351 [120]: 21122000
0.00339 [398]: 12112010
0.00314 [109]: 21201101
0.00293 [50]: 22020110
0.00268 [29]: 22102100
0.00261 [41]: 22100111
0.00260 [49]: 22020200

2^{dimer}, sa-CASSCF(8,8) with twenty singlet, three triplet and one quintet roots.

Q_1

0.89148 [0]: 22111100
0.00903 [238]: 11111111
0.00735 [10]: 22011110
0.00716 [7]: 22101101
0.00713 [34]: 21111200
0.00646 [128]: 12112100
0.00476 [28]: 21120110
0.00467 [16]: 21211010
0.00435 [27]: 21121001
0.00423 [19]: 21210101
0.00410 [114]: 12211001
0.00384 [115]: 12210110
0.00383 [123]: 12121010
0.00381 [126]: 12120101
0.00303 [67]: 21011210
0.00286 [51]: 21101201
0.00257 [145]: 12102101
0.00256 [161]: 12012110

T_1

0.30622 [0]: 22211000
0.14887 [11]: 22120100
0.13457 [10]: 22121000
0.11340 [1]: 22210100
0.07019 [49]: 22011200
0.03593 [24]: 22102100
0.03149 [27]: 22101200
0.02681 [46]: 22012100
0.01115 [119]: 21111110

0.01074 [367]: 12111101
0.00666 [120]: 21111101
0.00624 [366]: 12111110
0.00490 [77]: 21220010
0.00369 [325]: 12220001
0.00303 [80]: 21211100
0.00297 [465]: 11211011
0.00274 [16]: 22111010
0.00268 [78]: 21220001

T_2

0.21804 [1]: 22210100
0.21226 [11]: 22120100
0.20270 [10]: 22121000
0.06851 [0]: 22211000
0.05188 [46]: 22012100
0.05093 [24]: 22102100
0.04717 [27]: 22101200
0.01584 [49]: 22011200
0.01118 [120]: 21111101
0.01007 [366]: 12111110
0.00775 [119]: 21111110
0.00566 [367]: 12111101
0.00439 [78]: 21220001
0.00408 [324]: 12220010
0.00376 [106]: 21121100
0.00300 [19]: 22110110
0.00262 [325]: 12220001

T_3

0.21481 [4]: 22201100
0.21312 [40]: 22021100
0.20939 [14]: 22112000
0.20741 [18]: 22110200
0.04289 [15]: 22111100
0.00449 [82]: 21211001
0.00446 [111]: 21120101
0.00445 [84]: 21210110
0.00444 [107]: 21121010
0.00408 [328]: 12211010
0.00401 [357]: 12120110
0.00381 [355]: 12121001
0.00377 [332]: 12210101
0.00375 [28]: 22101110
0.00372 [115]: 21112100
0.00358 [51]: 22011101
0.00317 [365]: 12111200

S_0

0.55392 [0]: 22220000
0.11815 [20]: 22111100
0.03701 [5]: 22202000
0.03474 [49]: 22020200
0.03057 [71]: 22002200
0.02981 [45]: 22022000
0.02900 [9]: 22200200
0.01185 [125]: 21120110
0.01123 [100]: 21210101
0.01046 [364]: 12211001
0.01026 [389]: 12121010
0.00947 [96]: 21211010
0.00844 [123]: 21121001
0.00829 [393]: 12120101
0.00779 [366]: 12210110
0.00698 [91]: 21220100
0.00595 [357]: 12221000
0.00480 [491]: 11220011
0.00472 [17]: 22120010
0.00455 [4]: 22210001
0.00389 [19]: 22112000
0.00379 [23]: 22110200
0.00311 [553]: 11111111
0.00256 [187]: 21011210

S_1

0.22147 [20]: 22111100
0.15181 [5]: 22202000
0.15084 [45]: 22022000
0.15057 [9]: 22200200
0.14931 [49]: 22020200
0.01976 [6]: 22201100
0.01950 [46]: 22021100
0.01132 [19]: 22112000
0.01112 [23]: 22110200
0.00467 [125]: 21120110
0.00454 [96]: 21211010
0.00431 [100]: 21210101
0.00426 [123]: 21121001
0.00405 [364]: 12211001
0.00391 [389]: 12121010
0.00387 [366]: 12210110
0.00387 [393]: 12120101
0.00279 [38]: 22100210
0.00279 [147]: 21102110
0.00273 [165]: 21022100
0.00268 [65]: 22010201
0.00266 [104]: 21202100
0.00256 [183]: 21012101

S_2

0.25533 [16]: 22120100
0.22844 [1]: 22211000
0.05860 [29]: 22102100
0.05332 [58]: 22011200
0.04536 [21]: 22111010
0.03720 [25]: 22110101
0.02841 [50]: 22020110
0.02382 [8]: 22201001
0.02035 [72]: 22002110
0.01962 [95]: 21211100
0.01798 [78]: 22001201
0.01578 [48]: 22021001
0.01443 [10]: 22200110
0.01417 [388]: 12121100
0.01365 [124]: 21120200
0.01226 [134]: 21111110
0.00996 [402]: 12111101
0.00933 [361]: 12212000
0.00752 [146]: 21102200
0.00590 [448]: 12012200
0.00477 [109]: 21201101
0.00446 [120]: 21122000
0.00446 [155]: 21101111
0.00440 [436]: 12021110
0.00436 [365]: 12210200
0.00411 [140]: 21110201
0.00388 [457]: 12011111
0.00348 [398]: 12112010

0.00337 [7]: 22201010
0.00290 [102]: 21210011
0.00278 [15]: 22121000

$\mathbf{S_3}$

0.24184 [2]: 22210100
0.21865 [15]: 22121000
0.05584 [55]: 22012100
0.05207 [32]: 22101200
0.04154 [22]: 22111001
0.03899 [24]: 22110110
0.02336 [11]: 22200101
0.02311 [47]: 22021010
0.02016 [73]: 22002101
0.01893 [77]: 22001210
0.01800 [121]: 21121100
0.01779 [16]: 22120100
0.01767 [7]: 22201010
0.01652 [51]: 22020101
0.01445 [362]: 12211100
0.01248 [98]: 21210200
0.01190 [135]: 21111101
0.01029 [401]: 12111110
0.00858 [387]: 12122000
0.00732 [181]: 21012200
0.00634 [413]: 12102200
0.00567 [1]: 22211000
0.00533 [29]: 22102100
0.00489 [391]: 12120200
0.00485 [169]: 21021110
0.00472 [94]: 21212000
0.00463 [190]: 21011111
0.00423 [376]: 12201101
0.00421 [139]: 21110210
0.00394 [422]: 12101111
0.00341 [399]: 12112001
0.00281 [128]: 21120011
0.00259 [50]: 22020110

$\mathbf{S_4}$

0.28590 [1]: 22211000
0.24088 [16]: 22120100
0.06874 [10]: 22200110
0.06411 [15]: 22121000
0.06069 [50]: 22020110
0.03231 [365]: 12210200
0.03005 [361]: 12212000
0.01559 [21]: 22111010
0.01168 [388]: 12121100
0.01152 [391]: 12120200
0.01128 [387]: 12122000
0.00995 [51]: 22020101
0.00894 [398]: 12112010
0.00874 [11]: 22200101
0.00806 [24]: 22110110
0.00805 [436]: 12021110
0.00795 [8]: 22201001
0.00681 [362]: 12211100
0.00661 [48]: 22021001
0.00481 [120]: 21122000
0.00473 [22]: 22111001
0.00437 [406]: 12110210
0.00425 [134]: 21111110
0.00370 [36]: 22101011
0.00330 [375]: 12201110
0.00305 [67]: 22010111
0.00298 [402]: 12111101

$\mathbf{S_5}$

0.07297 [71]: 22002200
0.07249 [4]: 22210001
0.06091 [357]: 12221000
0.05976 [17]: 22120010
0.05694 [3]: 22210010
0.05641 [20]: 22111100
0.05634 [90]: 21221000
0.05484 [91]: 21220100
0.05122 [19]: 22112000
0.04266 [6]: 22201100
0.03533 [5]: 22202000
0.02947 [18]: 22120001
0.01941 [358]: 12220100
0.01936 [46]: 22021100
0.01535 [23]: 22110200
0.01438 [45]: 22022000
0.01405 [0]: 22220000
0.01219 [9]: 22200200
0.00762 [60]: 22011101
0.00680 [400]: 12111200
0.00671 [49]: 22020200
0.00666 [364]: 12211001
0.00650 [100]: 21210101
0.00638 [33]: 22101110
0.00624 [64]: 22010210
0.00610 [168]: 21021200
0.00609 [130]: 21112100
0.00608 [59]: 22011110
0.00586 [389]: 12121010
0.00579 [125]: 21120110
0.00571 [133]: 21111200
0.00533 [27]: 22110011
0.00496 [142]: 21110111
0.00461 [484]: 11221100
0.00447 [65]: 22010201
0.00424 [532]: 11121110
0.00394 [39]: 22100201
0.00370 [96]: 21211010
0.00358 [404]: 12111011
0.00357 [498]: 11211101
0.00353 [107]: 21201200
0.00352 [38]: 22100210
0.00322 [56]: 22012010
0.00320 [34]: 22101101
0.00314 [435]: 12021200
0.00299 [148]: 21102101
0.00292 [432]: 12022100
0.00280 [97]: 21211001
0.00268 [414]: 12102110
0.00264 [182]: 21012110
0.00259 [152]: 21101210

$\mathbf{S_6}$

0.07269 [18]: 22120001
0.05867 [358]: 12220100
0.05704 [23]: 22110200
0.05503 [46]: 22021100

0.05415 [17]: 22120010
0.05394 [91]: 21220100
0.05361 [3]: 22210010
0.04937 [71]: 22002200
0.04520 [90]: 21221000
0.04067 [20]: 22111100
0.03575 [4]: 22210001
0.03541 [6]: 22201100
0.03532 [19]: 22112000
0.03106 [49]: 22020200
0.02775 [357]: 12221000
0.01180 [9]: 22200200
0.01179 [33]: 22101110
0.01167 [130]: 21112100
0.00966 [60]: 22011101
0.00921 [0]: 22220000
0.00866 [31]: 22102001
0.00830 [45]: 22022000
0.00812 [400]: 12111200
0.00693 [56]: 22012010
0.00660 [371]: 12202100
0.00587 [107]: 21201200
0.00565 [125]: 21120110
0.00453 [30]: 22102010
0.00447 [100]: 21210101
0.00430 [188]: 21011201
0.00426 [168]: 21021200
0.00412 [34]: 22101101
0.00411 [152]: 21101210
0.00406 [39]: 22100201
0.00404 [182]: 21012110
0.00402 [432]: 12022100
0.00380 [389]: 12121010
0.00379 [454]: 12011210
0.00378 [64]: 22010210
0.00377 [148]: 21102101
0.00373 [104]: 21202100
0.00368 [126]: 21120101
0.00365 [137]: 21111011
0.00358 [27]: 22110011
0.00352 [397]: 12112100
0.00332 [414]: 12102110
0.00323 [420]: 12101201
0.00321 [450]: 12012101
0.00319 [404]: 12111011
0.00314 [484]: 11221100
0.00304 [364]: 12211001
0.00290 [498]: 11211101
0.00285 [392]: 12120110
0.00274 [497]: 11211110
0.00253 [57]: 22012001

S_7

0.27917 [2]: 22210100
0.22303 [15]: 22121000
0.07568 [51]: 22020101
0.07094 [11]: 22200101
0.04321 [391]: 12120200
0.03993 [387]: 12122000
0.03237 [16]: 22120100
0.02339 [22]: 22111001
0.02155 [1]: 22211000
0.01573 [362]: 12211100
0.01108 [376]: 12201101
0.01080 [399]: 12112001
0.01069 [10]: 22200110
0.01014 [365]: 12210200
0.00983 [361]: 12212000
0.00959 [50]: 22020110
0.00631 [388]: 12121100
0.00607 [407]: 12110201
0.00566 [437]: 12021101
0.00523 [25]: 22110101
0.00405 [135]: 21111101
0.00387 [401]: 12111110
0.00373 [21]: 22111010
0.00312 [41]: 22100111
0.00274 [62]: 22011011
0.00266 [131]: 21112010
0.00261 [108]: 21201110
0.00255 [68]: 22010102

S_8

0.10556 [7]: 22201010
0.10450 [47]: 22021010
0.08198 [94]: 21212000
0.08082 [15]: 22121000
0.07747 [98]: 21210200
0.06205 [2]: 22210100
0.05063 [24]: 22110110
0.04389 [121]: 21121100
0.02773 [22]: 22111001
0.02463 [11]: 22200101
0.02363 [169]: 21021110
0.02309 [362]: 12211100
0.02269 [51]: 22020101
0.02253 [131]: 21112010
0.02247 [139]: 21110210
0.02218 [108]: 21201110
0.01980 [387]: 12122000
0.01819 [391]: 12120200
0.01484 [437]: 12021101
0.01463 [407]: 12110201
0.01045 [95]: 21211100
0.00948 [21]: 22111010
0.00796 [399]: 12112001
0.00776 [376]: 12201101
0.00503 [1]: 22211000
0.00493 [134]: 21111110
0.00330 [16]: 22120100

S_9

0.21997 [23]: 22110200
0.19734 [19]: 22112000
0.10859 [90]: 21221000
0.09550 [358]: 12220100
0.03239 [60]: 22011101
0.02796 [20]: 22111100
0.02223 [39]: 22100201
0.02089 [33]: 22101110
0.02061 [9]: 22200200
0.01557 [390]: 12121001
0.01466 [126]: 21120101
0.01316 [31]: 22102001
0.00974 [99]: 21210110

0.00967 [432]: 12022100
0.00882 [130]: 21112100
0.00830 [5]: 22202000
0.00804 [91]: 21220100
0.00801 [400]: 12111200
0.00687 [56]: 22012010
0.00641 [168]: 21021200
0.00601 [367]: 12210101
0.00595 [18]: 22120001
0.00572 [85]: 22000211
0.00562 [64]: 22010210
0.00502 [104]: 21202100
0.00500 [75]: 22002011
0.00475 [46]: 22021100
0.00458 [107]: 21201200
0.00453 [363]: 12211010
0.00443 [49]: 22020200
0.00438 [420]: 12101201
0.00433 [97]: 21211001
0.00391 [435]: 12021200
0.00359 [148]: 21102101
0.00327 [17]: 22120010
0.00318 [65]: 22010201
0.00300 [30]: 22102010
0.00286 [71]: 22002200
0.00263 [122]: 21121010

S_{10}

0.09714 [8]: 22201001
0.09613 [48]: 22021001
0.08099 [120]: 21122000
0.07742 [124]: 21120200
0.05319 [25]: 22110101
0.04671 [95]: 21211100
0.03879 [50]: 22020110
0.03713 [10]: 22200110
0.03645 [21]: 22111010
0.03640 [1]: 22211000
0.03503 [16]: 22120100
0.03081 [361]: 12212000
0.02956 [365]: 12210200
0.02889 [388]: 12121100
0.02485 [132]: 21112001
0.02388 [170]: 21021101
0.02364 [109]: 21201101
0.02266 [140]: 21110201
0.01833 [375]: 12201110
0.01833 [406]: 12110210
0.01138 [398]: 12112010
0.01114 [436]: 12021110
0.01045 [121]: 21121100
0.00768 [22]: 22111001
0.00675 [2]: 22210100
0.00433 [362]: 12211100
0.00432 [135]: 21111101
0.00408 [24]: 22110110
0.00270 [401]: 12111110
0.00261 [51]: 22020101
0.00257 [11]: 22200101

S_{11}

0.11410 [9]: 22200200
0.10307 [4]: 22210001
0.08229 [45]: 22022000
0.08003 [17]: 22120010
0.06616 [46]: 22021100
0.06225 [3]: 22210010
0.04284 [6]: 22201100
0.03621 [18]: 22120001
0.03364 [23]: 22110200
0.03107 [49]: 22020200
0.02281 [64]: 22010210
0.02112 [38]: 22100210
0.01717 [65]: 22010201
0.01677 [366]: 12210110
0.01525 [59]: 22011110
0.01383 [5]: 22202000
0.01242 [374]: 12201200
0.01206 [397]: 12112100
0.01193 [400]: 12111200
0.01169 [34]: 22101101
0.01041 [363]: 12211010
0.00862 [389]: 12121010
0.00861 [90]: 21221000
0.00748 [435]: 12021200
0.00748 [357]: 12221000
0.00730 [39]: 22100201
0.00699 [393]: 12120101
0.00657 [60]: 22011101
0.00570 [367]: 12210101
0.00532 [364]: 12211001
0.00527 [33]: 22101110
0.00512 [123]: 21121001
0.00510 [133]: 21111200
0.00500 [419]: 12101210
0.00500 [371]: 12202100
0.00461 [392]: 12120110
0.00442 [91]: 21220100
0.00428 [358]: 12220100
0.00344 [455]: 12011201
0.00299 [97]: 21211001
0.00276 [454]: 12011210
0.00276 [57]: 22012001

S_{12}

0.10998 [46]: 22021100
0.09529 [18]: 22120001
0.08403 [6]: 22201100
0.08202 [5]: 22202000
0.07884 [3]: 22210010
0.06943 [17]: 22120010
0.04538 [4]: 22210001
0.03018 [45]: 22022000
0.02978 [49]: 22020200
0.02233 [33]: 22101110
0.01903 [392]: 12120110
0.01880 [59]: 22011110
0.01488 [39]: 22100201
0.01442 [60]: 22011101
0.01425 [397]: 12112100
0.01388 [64]: 22010210
0.01375 [393]: 12120101
0.01332 [371]: 12202100
0.01288 [38]: 22100210
0.01272 [367]: 12210101

0.01221 [400]: 12111200
0.01194 [34]: 22101101
0.01121 [19]: 22112000
0.01115 [432]: 12022100
0.01005 [366]: 12210110
0.00792 [9]: 22200200
0.00678 [374]: 12201200
0.00635 [414]: 12102110
0.00596 [363]: 12211010
0.00573 [30]: 22102010
0.00523 [364]: 12211001
0.00494 [65]: 22010201
0.00467 [390]: 12121001
0.00447 [450]: 12012101
0.00389 [435]: 12021200
0.00336 [20]: 22111100
0.00293 [357]: 12221000
0.00270 [419]: 12101210

S_{13}

0.08939 [45]: 22022000
0.08715 [49]: 22020200
0.08400 [357]: 12221000
0.07682 [5]: 22202000
0.05030 [91]: 21220100
0.04322 [6]: 22201100
0.04005 [133]: 21111200
0.03815 [9]: 22200200
0.03318 [34]: 22101101
0.03277 [57]: 22012001
0.02901 [71]: 22002200
0.02648 [59]: 22011110
0.02202 [123]: 21121001
0.02054 [364]: 12211001
0.01957 [165]: 21022100
0.01955 [30]: 22102010
0.01732 [27]: 22110011
0.01533 [96]: 21211010
0.01409 [104]: 21202100
0.01304 [484]: 11221100
0.01218 [18]: 22120001
0.01183 [3]: 22210010
0.00935 [20]: 22111100
0.00786 [389]: 12121010
0.00711 [4]: 22210001
0.00645 [183]: 21012101
0.00563 [142]: 21110111
0.00549 [393]: 12120101
0.00548 [64]: 22010210
0.00517 [147]: 21102110
0.00413 [90]: 21221000
0.00411 [432]: 12022100
0.00408 [220]: 20220200
0.00407 [168]: 21021200
0.00403 [60]: 22011101
0.00397 [153]: 21101201
0.00396 [392]: 12120110
0.00390 [366]: 12210110
0.00386 [76]: 22002002
0.00383 [400]: 12111200
0.00371 [39]: 22100201
0.00341 [74]: 22002020
0.00337 [404]: 12111011
0.00331 [33]: 22101110
0.00329 [125]: 21120110
0.00318 [216]: 20222000
0.00295 [367]: 12210101
0.00288 [19]: 22112000
0.00277 [374]: 12201200
0.00277 [390]: 12121001
0.00270 [187]: 21011210
0.00262 [498]: 11211101
0.00254 [532]: 11121110
0.00253 [23]: 22110200

S_{14}

0.08945 [71]: 22002200
0.06360 [91]: 21220100
0.05768 [49]: 22020200
0.05054 [34]: 22101101
0.04807 [397]: 12112100
0.04529 [65]: 22010201
0.04330 [27]: 22110011
0.03783 [59]: 22011110
0.03691 [9]: 22200200
0.03469 [5]: 22202000
0.03130 [484]: 11221100
0.02935 [435]: 12021200
0.02933 [38]: 22100210
0.02410 [393]: 12120101
0.02399 [100]: 21210101
0.02142 [125]: 21120110
0.01767 [374]: 12201200
0.01662 [45]: 22022000
0.01630 [366]: 12210110
0.01539 [357]: 12221000
0.01317 [133]: 21111200
0.01072 [389]: 12121010
0.00965 [96]: 21211010
0.00851 [123]: 21121001
0.00803 [104]: 21202100
0.00765 [455]: 12011201
0.00698 [142]: 21110111
0.00692 [404]: 12111011
0.00692 [30]: 22102010
0.00692 [6]: 22201100
0.00666 [165]: 21022100
0.00644 [17]: 22120010
0.00574 [364]: 12211001
0.00568 [498]: 11211101
0.00561 [532]: 11121110
0.00533 [57]: 22012001
0.00523 [415]: 12102101
0.00470 [54]: 22020002
0.00461 [419]: 12101210
0.00438 [754]: 02220200
0.00427 [12]: 22200020
0.00423 [20]: 22111100
0.00413 [80]: 22001111
0.00391 [750]: 02222000
0.00388 [86]: 22000202
0.00379 [371]: 12202100
0.00372 [69]: 22010021
0.00361 [84]: 22000220

0.00359 [44]: 22100012
0.00333 [544]: 11112200
0.00318 [14]: 22200002
0.00310 [46]: 22021100
0.00309 [449]: 12012110
0.00270 [52]: 22020020
0.00266 [627]: 10221200

S_{15}

0.07437 [120]: 21122000
0.07304 [124]: 21120200
0.06976 [8]: 22201001
0.06806 [48]: 22021001
0.06670 [10]: 22200110
0.06588 [361]: 12212000
0.06549 [50]: 22020110
0.06416 [365]: 12210200
0.05248 [95]: 21211100
0.04650 [25]: 22110101
0.04500 [21]: 22111010
0.04460 [388]: 12121100
0.02318 [134]: 21111110
0.01629 [402]: 12111101
0.01470 [105]: 21202010
0.01133 [121]: 21121100
0.01038 [113]: 21200210
0.00983 [166]: 21022010
0.00923 [442]: 12020201
0.00851 [362]: 12211100
0.00738 [24]: 22110110
0.00730 [170]: 21021101
0.00727 [375]: 12201110
0.00621 [381]: 12200201
0.00599 [434]: 12022001
0.00574 [174]: 21020210
0.00558 [22]: 22111001
0.00510 [436]: 12021110
0.00431 [109]: 21201101
0.00395 [373]: 12202001

S_{16}

0.07385 [98]: 21210200
0.07244 [7]: 22201010
0.07220 [94]: 21212000
0.07031 [47]: 22021010
0.06270 [11]: 22200101
0.06222 [391]: 12120200
0.06115 [387]: 12122000
0.05967 [51]: 22020101
0.05361 [121]: 21121100
0.04730 [24]: 22110110
0.04674 [22]: 22111001
0.04443 [362]: 12211100
0.02059 [135]: 21111101
0.01695 [401]: 12111110
0.01320 [167]: 21022001
0.01118 [95]: 21211100
0.00978 [380]: 12200210
0.00884 [175]: 21020201
0.00842 [388]: 12121100
0.00838 [106]: 21202001
0.00772 [108]: 21201110
0.00741 [21]: 22111010
0.00705 [437]: 12021101
0.00667 [441]: 12020210
0.00640 [372]: 12202010
0.00537 [25]: 22110101
0.00503 [169]: 21021110
0.00503 [114]: 21200201
0.00462 [376]: 12201101
0.00377 [433]: 12022010
0.00350 [48]: 22021001
0.00336 [8]: 22201001
0.00286 [365]: 12210200
0.00253 [361]: 12212000

S_{17}

0.04134 [31]: 22102001
0.03742 [30]: 22102010
0.03506 [126]: 21120101
0.03339 [56]: 22012010
0.03171 [130]: 21112100
0.03169 [125]: 21120110
0.02987 [122]: 21121010
0.02937 [104]: 21202100
0.02826 [99]: 21210110
0.02805 [165]: 21022100
0.02344 [107]: 21201200
0.02324 [168]: 21021200
0.02295 [33]: 22101110
0.02033 [39]: 22100201
0.01957 [97]: 21211001
0.01932 [100]: 21210101
0.01925 [57]: 22012001
0.01913 [38]: 22100210
0.01889 [392]: 12120110
0.01816 [123]: 21121001
0.01768 [371]: 12202100
0.01668 [64]: 22010210
0.01665 [432]: 12022100
0.01555 [34]: 22101101
0.01447 [133]: 21111200
0.01431 [60]: 22011101
0.01431 [96]: 21211010
0.01383 [393]: 12120101
0.01306 [390]: 12121001
0.01207 [59]: 22011110
0.01195 [46]: 22021100
0.01186 [367]: 12210101
0.01177 [18]: 22120001
0.01113 [65]: 22010201
0.01084 [389]: 12121010
0.01074 [363]: 12211010
0.01046 [6]: 22201100
0.01028 [148]: 21102101
0.00980 [366]: 12210110
0.00942 [147]: 21102110
0.00907 [3]: 22210010
0.00891 [152]: 21101210
0.00890 [17]: 22120010
0.00875 [397]: 12112100
0.00798 [182]: 21012110
0.00792 [435]: 12021200
0.00756 [374]: 12201200
0.00643 [415]: 12102101

0.00618 [153]: 21101201
0.00618 [400]: 12111200
0.00605 [5]: 22202000
0.00598 [188]: 21011201
0.00549 [364]: 12211001
0.00533 [414]: 12102110
0.00513 [183]: 21012101
0.00509 [45]: 22022000
0.00483 [449]: 12012110
0.00477 [4]: 22210001
0.00459 [187]: 21011210
0.00340 [420]: 12101201
0.00303 [454]: 12011210
0.00295 [450]: 12012101

S_{18}

0.04188 [96]: 21211010
0.03469 [57]: 22012001
0.03464 [59]: 22011110
0.03141 [56]: 22012010
0.03071 [97]: 21211001
0.03044 [133]: 21111200
0.02964 [30]: 22102010
0.02771 [107]: 21201200
0.02491 [123]: 21121001
0.02373 [104]: 21202100
0.02248 [60]: 22011101
0.02247 [168]: 21021200
0.02203 [363]: 12211010
0.02185 [364]: 12211001
0.02159 [374]: 12201200
0.02104 [100]: 21210101
0.02080 [122]: 21121010
0.01948 [165]: 21022100
0.01928 [33]: 22101110
0.01855 [65]: 22010201
0.01830 [435]: 12021200
0.01820 [34]: 22101101
0.01800 [389]: 12121010
0.01770 [31]: 22102001
0.01667 [64]: 22010210
0.01641 [99]: 21210110
0.01585 [38]: 22100210
0.01568 [125]: 21120110
0.01424 [49]: 22020200
0.01409 [366]: 12210110
0.01385 [130]: 21112100
0.01278 [187]: 21011210
0.01118 [400]: 12111200
0.01114 [390]: 12121001
0.01038 [4]: 22210001
0.00978 [371]: 12202100
0.00936 [188]: 21011201
0.00867 [367]: 12210101
0.00859 [397]: 12112100
0.00846 [153]: 21101201
0.00846 [126]: 21120101
0.00829 [393]: 12120101
0.00784 [39]: 22100201
0.00750 [6]: 22201100
0.00717 [3]: 22210010
0.00700 [432]: 12022100
0.00650 [454]: 12011210
0.00632 [449]: 12012110
0.00618 [17]: 22120010
0.00604 [152]: 21101210
0.00596 [18]: 22120001
0.00593 [392]: 12120110
0.00519 [183]: 21012101
0.00497 [46]: 22021100
0.00495 [415]: 12102101
0.00490 [147]: 21102110
0.00478 [5]: 22202000
0.00455 [182]: 21012110
0.00412 [9]: 22200200
0.00410 [455]: 12011201
0.00356 [420]: 12101201
0.00331 [419]: 12101210
0.00287 [450]: 12012101

S_{19}

0.04777 [27]: 22110011
0.04198 [123]: 21121001
0.04068 [96]: 21211010
0.03903 [100]: 21210101
0.03776 [125]: 21120110
0.03625 [366]: 12210110
0.03611 [12]: 22200020
0.03611 [389]: 12121010
0.03609 [52]: 22020020
0.03496 [364]: 12211001
0.03255 [393]: 12120101
0.03186 [14]: 22200002
0.03155 [54]: 22020002
0.03004 [484]: 11221100
0.02941 [404]: 12111011
0.02895 [142]: 21110111
0.02651 [216]: 20222000
0.02598 [220]: 20220200
0.02353 [498]: 11211101
0.02323 [532]: 11121110
0.01669 [750]: 02222000
0.01639 [553]: 11111111
0.01621 [754]: 02220200
0.00892 [115]: 21200120
0.00846 [176]: 21020120
0.00799 [178]: 21020102
0.00757 [117]: 21200102
0.00739 [438]: 12021020
0.00721 [262]: 20122010
0.00712 [270]: 20120210
0.00704 [377]: 12201020
0.00689 [236]: 20210201
0.00689 [228]: 20212001
0.00672 [379]: 12201002
0.00635 [136]: 21111020
0.00628 [440]: 12021002
0.00555 [138]: 21111002
0.00518 [408]: 12110120
0.00452 [796]: 02122010
0.00452 [230]: 20211110
0.00451 [410]: 12110102
0.00451 [804]: 02120210
0.00445 [762]: 02212001

0.00435 [111]: 21201011
0.00431 [502]: 11210210
0.00430 [172]: 21021011
0.00429 [770]: 02210201
0.00424 [494]: 11212010
0.00420 [266]: 20121101
0.00408 [538]: 11120201
0.00401 [530]: 11122001
0.00359 [383]: 12200111
0.00357 [444]: 12020111
0.00345 [217]: 20221100
0.00275 [26]: 22110020
0.00267 [764]: 02211110

3^{dimer}, sa-CASSCF(8,8) with eight singlet, three triplet and one quintet roots.

Q_1
0.91283 [0]: 22111100
0.00771 [10]: 22011110
0.00651 [34]: 21111200
0.00599 [115]: 12210110
0.00597 [128]: 12112100
0.00579 [7]: 22101101
0.00466 [27]: 21121001
0.00446 [161]: 12012110
0.00404 [51]: 21101201
0.00368 [379]: 02111120
0.00361 [17]: 21211001
0.00348 [125]: 12120110
0.00314 [97]: 20111102
0.00265 [96]: 20111111

T_1
0.34725 [10]: 22121000
0.29798 [0]: 22211000
0.13048 [27]: 22101200
0.08539 [49]: 22011200
0.02461 [120]: 21111101
0.01718 [11]: 22120100
0.01161 [119]: 21111110
0.00683 [24]: 22102100
0.00677 [324]: 12220010
0.00427 [94]: 21201101
0.00389 [16]: 22111010
0.00348 [155]: 21021101
0.00286 [80]: 21211100

T_2
0.42413 [1]: 22210100
0.21744 [11]: 22120100
0.13144 [46]: 22012100
0.08846 [24]: 22102100
0.02817 [366]: 12111110
0.01750 [10]: 22121000
0.00811 [367]: 12111101
0.00671 [27]: 22101200
0.00594 [78]: 21220001
0.00480 [340]: 12201110
0.00399 [327]: 12211100
0.00390 [401]: 12021110
0.00284 [77]: 21220010
0.00276 [83]: 21210200
0.00266 [19]: 22110110

T_3
0.44378 [15]: 22111100
0.23992 [4]: 22201100
0.19555 [40]: 22021100
0.01301 [14]: 22112000
0.01256 [18]: 22110200
0.00602 [331]: 12210110
0.00476 [108]: 21121001
0.00394 [414]: 12012110
0.00382 [50]: 22011110
0.00372 [138]: 21101201
0.00353 [118]: 21111200
0.00350 [357]: 12120110
0.00347 [82]: 21211001
0.00346 [28]: 22101110
0.00321 [29]: 22101101
0.00303 [362]: 12112100
0.00279 [379]: 12102110
0.00258 [51]: 22011101

S_0
0.47739 [0]: 22220000
0.07812 [19]: 22112000
0.07161 [23]: 22110200
0.06511 [9]: 22200200
0.05598 [71]: 22002200
0.05254 [45]: 22022000
0.03194 [5]: 22202000
0.02194 [49]: 22020200
0.01621 [389]: 12121010
0.01561 [100]: 21210101
0.01044 [363]: 12211010
0.00890 [20]: 22111100
0.00841 [126]: 21120101
0.00598 [99]: 21210110
0.00574 [419]: 12101210
0.00484 [125]: 21120110
0.00474 [183]: 21012101
0.00414 [364]: 12211001
0.00402 [390]: 12121001
0.00360 [357]: 12221000
0.00358 [148]: 21102101
0.00345 [454]: 12011210
0.00321 [91]: 21220100
0.00254 [17]: 22120010

S_1
0.33807 [6]: 22201100
0.27553 [46]: 22021100
0.24882 [20]: 22111100
0.01045 [5]: 22202000
0.01008 [9]: 22200200
0.00850 [45]: 22022000
0.00824 [49]: 22020200
0.00593 [366]: 12210110

0.00480 [33]: 22101110
0.00472 [123]: 21121001
0.00444 [1]: 22211000
0.00362 [449]: 12012110
0.00361 [392]: 12120110
0.00357 [60]: 22011101
0.00351 [97]: 21211001
0.00349 [153]: 21101201
0.00314 [414]: 12102110
0.00292 [15]: 22121000
0.00252 [168]: 21021200

S_2

0.13151 [3]: 22210010
0.09178 [357]: 12221000
0.08271 [19]: 22112000
0.06635 [91]: 21220100
0.06515 [9]: 22200200
0.05662 [18]: 22120001
0.05610 [23]: 22110200
0.03870 [5]: 22202000
0.03708 [45]: 22022000
0.02495 [38]: 22100210
0.02036 [374]: 12201200
0.01878 [400]: 12111200
0.01752 [130]: 21112100
0.01664 [64]: 22010210
0.01648 [419]: 12101210
0.01504 [17]: 22120010
0.01435 [57]: 22012001
0.01149 [389]: 12121010
0.01130 [183]: 21012101
0.01070 [56]: 22012010
0.01011 [31]: 22102001
0.00965 [49]: 22020200
0.00918 [454]: 12011210
0.00881 [165]: 21022100
0.00873 [148]: 21102101
0.00851 [104]: 21202100
0.00842 [4]: 22210001
0.00692 [363]: 12211010
0.00655 [30]: 22102010
0.00619 [182]: 21012110
0.00611 [6]: 22201100
0.00513 [142]: 21110111
0.00492 [147]: 21102110
0.00488 [39]: 22100201
0.00422 [435]: 12021200
0.00415 [99]: 21210110
0.00400 [497]: 11211110
0.00392 [100]: 21210101
0.00391 [420]: 12101201
0.00361 [126]: 21120101
0.00353 [404]: 12111011
0.00327 [750]: 02222000
0.00308 [533]: 11121101
0.00282 [65]: 22010201
0.00267 [26]: 22110020
0.00254 [46]: 22021100

S_3

0.14457 [71]: 22002200
0.11061 [17]: 22120010
0.10174 [4]: 22210001
0.10165 [91]: 21220100
0.07955 [357]: 12221000
0.04231 [23]: 22110200
0.03899 [0]: 22220000
0.02854 [9]: 22200200
0.02551 [19]: 22112000
0.02363 [45]: 22022000
0.01936 [100]: 21210101
0.01626 [389]: 12121010
0.01482 [49]: 22020200
0.01278 [130]: 21112100
0.01249 [57]: 22012001
0.01125 [38]: 22100210
0.00840 [125]: 21120110
0.00827 [31]: 22102001
0.00819 [364]: 12211001
0.00806 [165]: 21022100
0.00780 [400]: 12111200
0.00754 [404]: 12111011
0.00750 [532]: 11121110
0.00746 [498]: 11211101
0.00717 [5]: 22202000
0.00672 [56]: 22012010
0.00670 [64]: 22010210
0.00669 [363]: 12211010
0.00667 [374]: 12201200
0.00644 [126]: 21120101
0.00581 [142]: 21110111
0.00489 [30]: 22102010
0.00484 [104]: 21202100
0.00462 [553]: 11111111
0.00459 [18]: 22120001
0.00457 [27]: 22110011
0.00419 [99]: 21210110
0.00401 [20]: 22111100
0.00399 [220]: 20220200
0.00354 [2]: 22210100
0.00296 [750]: 02222000
0.00267 [90]: 21221000
0.00252 [39]: 22100201

S_4

0.21245 [16]: 22120100
0.07885 [15]: 22121000
0.06107 [2]: 22210100
0.05850 [29]: 22102100
0.04284 [25]: 22110101
0.03119 [1]: 22211000
0.03039 [55]: 22012100
0.02815 [21]: 22111010
0.02721 [98]: 21210200
0.02538 [32]: 22101200
0.02195 [47]: 22021010

0.02123 [73]: 22002101
0.02087 [51]: 22020101
0.02062 [72]: 22002110
0.01746 [24]: 22110110
0.01584 [124]: 21120200
0.01572 [10]: 22200110
0.01404 [58]: 22011200
0.01388 [77]: 22001210
0.01225 [401]: 12111110
0.01218 [388]: 12121100
0.01186 [50]: 22020110
0.01169 [361]: 12212000
0.00928 [387]: 12122000
0.00867 [48]: 22021001
0.00863 [11]: 22200101
0.00736 [181]: 21012200
0.00709 [146]: 21102200
0.00675 [402]: 12111101
0.00639 [413]: 12102200
0.00581 [135]: 21111101
0.00551 [78]: 22001201
0.00449 [140]: 21110201
0.00390 [359]: 12220010
0.00363 [422]: 12101111
0.00339 [398]: 12112010
0.00339 [114]: 21200201
0.00312 [201]: 21002201
0.00276 [457]: 12011111
0.00273 [92]: 21220010
0.00265 [93]: 21220001

S_5

0.22531 [15]: 22121000
0.06856 [32]: 22101200
0.06567 [16]: 22120100
0.04532 [47]: 22021010
0.04501 [2]: 22210100
0.03507 [21]: 22111010
0.03322 [77]: 22001210
0.02782 [1]: 22211000
0.02387 [387]: 12122000
0.02071 [55]: 22012100
0.01890 [24]: 22110110
0.01841 [361]: 12212000
0.01794 [25]: 22110101
0.01672 [29]: 22102100
0.01516 [51]: 22020101
0.01429 [98]: 21210200
0.01381 [73]: 22002101
0.01133 [135]: 21111101
0.01111 [58]: 22011200
0.01070 [22]: 22111001
0.00929 [413]: 12102200
0.00883 [33]: 22101110
0.00799 [7]: 22201010
0.00769 [46]: 22021100
0.00735 [6]: 22201100
0.00724 [59]: 22011110
0.00708 [48]: 22021001
0.00692 [121]: 21121100
0.00650 [401]: 12111110
0.00556 [10]: 22200110
0.00539 [124]: 21120200
0.00529 [366]: 12210110
0.00488 [359]: 12220010
0.00482 [72]: 22002110
0.00463 [11]: 22200101
0.00455 [146]: 21102200
0.00454 [433]: 12022010
0.00443 [398]: 12112010
0.00414 [448]: 12012200
0.00412 [134]: 21111110
0.00398 [358]: 12220100
0.00368 [467]: 12002210
0.00365 [78]: 22001201
0.00360 [190]: 21011111
0.00324 [181]: 21012200
0.00286 [402]: 12111101
0.00285 [371]: 12202100
0.00284 [107]: 21201200

S_6

0.18848 [1]: 22211000
0.14492 [95]: 21211100
0.11532 [7]: 22201010
0.07961 [15]: 22121000
0.07815 [47]: 22021010
0.05156 [21]: 22111010
0.04772 [121]: 21121100
0.04152 [134]: 21111110
0.03159 [22]: 22111001
0.03108 [135]: 21111101
0.02728 [8]: 22201001
0.02505 [48]: 22021001
0.01617 [108]: 21201110
0.01612 [169]: 21021110
0.01389 [170]: 21021101
0.00714 [109]: 21201101
0.00462 [61]: 22011020
0.00438 [94]: 21212000
0.00415 [98]: 21210200
0.00314 [35]: 22101020

S_7

0.17219 [362]: 12211100
0.14791 [10]: 22200110
0.11660 [16]: 22120100
0.11008 [2]: 22210100
0.10609 [50]: 22020110
0.07878 [24]: 22110110
0.06591 [401]: 12111110
0.03335 [388]: 12121100
0.03185 [436]: 12021110
0.02851 [375]: 12201110
0.00725 [402]: 12111101
0.00537 [25]: 22110101
0.00488 [361]: 12212000

0.00471 [365]: 12210200
0.00358 [437]: 12021101
0.00269 [9]: 22200200
0.00268 [41]: 22100111

S_8

0.24804 [2]: 22210100
0.12736 [388]: 12121100
0.12602 [11]: 22200101
0.12057 [16]: 22120100
0.05911 [51]: 22020101
0.04675 [362]: 12211100
0.02935 [402]: 12111101
0.02681 [25]: 22110101
0.02325 [376]: 12201101
0.01762 [401]: 12111110
0.01733 [375]: 12201110
0.01566 [24]: 22110110
0.01158 [50]: 22020110
0.01060 [437]: 12021101
0.00723 [436]: 12021110
0.00455 [42]: 22100102
0.00395 [29]: 22102100
0.00379 [49]: 22020200
0.00370 [17]: 22120010
0.00362 [387]: 12122000
0.00340 [391]: 12120200
0.00330 [55]: 22012100
0.00278 [68]: 22010102
0.00258 [10]: 22200110

S_9

0.27111 [1]: 22211000
0.15608 [121]: 21121100
0.12001 [8]: 22201001
0.07661 [48]: 22021001
0.06748 [15]: 22121000
0.05599 [22]: 22111001
0.04255 [135]: 21111101
0.03989 [109]: 21201101
0.03457 [95]: 21211100
0.01964 [170]: 21021101
0.00632 [134]: 21111110
0.00614 [108]: 21201110
0.00548 [7]: 22201010
0.00527 [21]: 22111010
0.00408 [124]: 21120200
0.00402 [120]: 21122000
0.00316 [36]: 22101011
0.00313 [62]: 22011011
0.00297 [32]: 22101200
0.00287 [20]: 22111100
0.00285 [2]: 22210100
0.00267 [398]: 12112010

S_{10}

0.17358 [18]: 22120001
0.12066 [49]: 22020200
0.11363 [9]: 22200200
0.10349 [3]: 22210010
0.05495 [45]: 22022000
0.03540 [5]: 22202000
0.03200 [39]: 22100201
0.02619 [364]: 12211001
0.02311 [400]: 12111200
0.02181 [4]: 22210001
0.02044 [65]: 22010201
0.01715 [23]: 22110200
0.01560 [38]: 22100210
0.01418 [390]: 12121001
0.01191 [57]: 22012001
0.01163 [64]: 22010210
0.01138 [374]: 12201200
0.01108 [165]: 21022100
0.01085 [435]: 12021200
0.01077 [125]: 21120110
0.01047 [46]: 22021100
0.00885 [130]: 21112100
0.00863 [31]: 22102001
0.00776 [100]: 21210101
0.00753 [6]: 22201100
0.00745 [455]: 12011201
0.00743 [56]: 22012010
0.00734 [357]: 12221000
0.00674 [420]: 12101201
0.00564 [183]: 21012101
0.00453 [30]: 22102010
0.00437 [419]: 12101210
0.00424 [389]: 12121010
0.00377 [53]: 22020011
0.00263 [91]: 21220100

S_{11}

0.12726 [5]: 22202000
0.12341 [4]: 22210001
0.10284 [45]: 22022000
0.08770 [17]: 22120010
0.05049 [56]: 22012010
0.05004 [3]: 22210010
0.04592 [9]: 22200200
0.03337 [30]: 22102010
0.03151 [99]: 21210110
0.02588 [130]: 21112100
0.02368 [49]: 22020200
0.01941 [38]: 22100210
0.01856 [104]: 21202100
0.01444 [100]: 21210101
0.01443 [374]: 12201200
0.01279 [364]: 12211001
0.01230 [64]: 22010210
0.01221 [71]: 22002200
0.01206 [27]: 22110011
0.01138 [19]: 22112000
0.00998 [363]: 12211010
0.00923 [147]: 21102110
0.00870 [400]: 12111200
0.00813 [484]: 11221100
0.00759 [182]: 21012110

0.00574 [419]: 12101210
0.00546 [126]: 21120101
0.00483 [532]: 11121110
0.00476 [91]: 21220100
0.00395 [404]: 12111011
0.00372 [142]: 21110111
0.00344 [390]: 12121001
0.00341 [12]: 22200020
0.00335 [59]: 22011110
0.00324 [11]: 22200101
0.00315 [2]: 22210100
0.00303 [52]: 22020020
0.00253 [165]: 21022100

S_{12}

0.18313 [46]: 22021100
0.12228 [358]: 12220100
0.10801 [6]: 22201100
0.09286 [60]: 22011101
0.07755 [20]: 22111100
0.05646 [34]: 22101101
0.05235 [432]: 12022100
0.04538 [367]: 12210101
0.04131 [393]: 12120101
0.02013 [397]: 12112100
0.01732 [450]: 12012101
0.01361 [392]: 12120110
0.01287 [415]: 12102101
0.01224 [90]: 21221000
0.00794 [123]: 21121001
0.00739 [81]: 22001102
0.00679 [366]: 12210110
0.00665 [45]: 22022000
0.00558 [449]: 12012110
0.00521 [32]: 22101200
0.00442 [49]: 22020200
0.00396 [59]: 22011110
0.00390 [133]: 21111200
0.00379 [371]: 12202100
0.00368 [9]: 22200200
0.00343 [188]: 21011201
0.00333 [97]: 21211001
0.00300 [409]: 12110111
0.00283 [57]: 22012001
0.00281 [5]: 22202000
0.00258 [168]: 21021200

S_{13}

0.27390 [20]: 22111100
0.11643 [90]: 21221000
0.09500 [34]: 22101101
0.08027 [123]: 21121001
0.07174 [6]: 22201100
0.04787 [60]: 22011101
0.04210 [168]: 21021200
0.03983 [133]: 21111200
0.03294 [97]: 21211001
0.02679 [153]: 21101201
0.01470 [46]: 22021100
0.01444 [33]: 22101110
0.01246 [188]: 21011201
0.01173 [358]: 12220100
0.00842 [19]: 22112000
0.00830 [23]: 22110200
0.00718 [80]: 22001111
0.00637 [432]: 12022100
0.00595 [59]: 22011110
0.00434 [367]: 12210101
0.00415 [393]: 12120101
0.00323 [96]: 21211010
0.00281 [137]: 21111011
0.00278 [414]: 12102110
0.00268 [79]: 22001120

S_{14}

0.10338 [107]: 21201200
0.08485 [33]: 22101110
0.06817 [59]: 22011110
0.06691 [20]: 22111100
0.06441 [122]: 21121010
0.06143 [34]: 22101101
0.05715 [96]: 21211010
0.05554 [123]: 21121001
0.05389 [90]: 21221000
0.05216 [97]: 21211001
0.03857 [133]: 21111200
0.03426 [60]: 22011101
0.03103 [168]: 21021200
0.02698 [6]: 22201100
0.02417 [153]: 21101201
0.02274 [152]: 21101210
0.01854 [187]: 21011210
0.01397 [188]: 21011201
0.01137 [46]: 22021100
0.00368 [15]: 22121000
0.00325 [79]: 22001120
0.00303 [366]: 12210110
0.00251 [22]: 22111001

S_{15}

0.14112 [366]: 12210110
0.12701 [59]: 22011110
0.10249 [33]: 22101110
0.08831 [371]: 12202100
0.07017 [392]: 12120110
0.06290 [397]: 12112100
0.04520 [449]: 12012110
0.04340 [358]: 12220100
0.03601 [46]: 22021100
0.03201 [20]: 22111100
0.02891 [414]: 12102110
0.02675 [432]: 12022100
0.01450 [367]: 12210101
0.01417 [15]: 22121000
0.01380 [6]: 22201100
0.01146 [60]: 22011101
0.00677 [393]: 12120101
0.00604 [58]: 22011200

0.00542 [415]: 12102101
0.00371 [387]: 12122000
0.00369 [21]: 22111010
0.00360 [450]: 12012101
0.00341 [34]: 22101101
0.00303 [77]: 22001210
0.00284 [56]: 22012010
0.00271 [64]: 22010210
0.00268 [30]: 22102010
0.00265 [48]: 22021001
0.00260 [1]: 22211000

S_{16}

0.07764 [71]: 22002200
0.05620 [17]: 22120010
0.05253 [484]: 11221100
0.05210 [27]: 22110011
0.04485 [57]: 22012001
0.04046 [125]: 21120110
0.03891 [400]: 12111200
0.03106 [130]: 21112100
0.03034 [31]: 22102001
0.03002 [39]: 22100201
0.02996 [165]: 21022100
0.02598 [49]: 22020200
0.02567 [390]: 12121001
0.02261 [45]: 22022000
0.02251 [38]: 22100210
0.02207 [364]: 12211001
0.02122 [65]: 22010201
0.01741 [435]: 12021200
0.01605 [389]: 12121010
0.01504 [374]: 12201200
0.01412 [99]: 21210110
0.01326 [64]: 22010210
0.01294 [498]: 11211101
0.01138 [404]: 12111011
0.01137 [183]: 21012101
0.01089 [532]: 11121110
0.01074 [142]: 21110111
0.01003 [363]: 12211010
0.01002 [126]: 21120101
0.00908 [23]: 22110200
0.00706 [420]: 12101201
0.00704 [12]: 22200020
0.00696 [18]: 22120001
0.00672 [4]: 22210001
0.00670 [148]: 21102101
0.00668 [100]: 21210101
0.00582 [91]: 21220100
0.00572 [104]: 21202100
0.00543 [5]: 22202000
0.00535 [9]: 22200200
0.00532 [54]: 22020002
0.00527 [455]: 12011201
0.00509 [357]: 12221000
0.00505 [419]: 12101210
0.00472 [454]: 12011210
0.00450 [497]: 11211110
0.00412 [69]: 22010021
0.00389 [14]: 22200002
0.00367 [177]: 21020111
0.00359 [34]: 22101101
0.00328 [44]: 22100012
0.00326 [56]: 22012010
0.00305 [141]: 21110120
0.00304 [13]: 22200011
0.00290 [52]: 22020020
0.00288 [30]: 22102010
0.00257 [533]: 11121101

S_{17}

0.15752 [121]: 21121100
0.12396 [95]: 21211100
0.09833 [8]: 22201001
0.05528 [48]: 22021001
0.05155 [22]: 22111001
0.04393 [362]: 12211100
0.03321 [170]: 21021101
0.03170 [10]: 22200110
0.03168 [109]: 21201101
0.03048 [388]: 12121100
0.02922 [135]: 21111101
0.02594 [108]: 21201110
0.02310 [21]: 22111010
0.02295 [7]: 22201010
0.01976 [134]: 21111110
0.01644 [50]: 22020110
0.01539 [375]: 12201110
0.01423 [47]: 22021010
0.01231 [169]: 21021110
0.01059 [401]: 12111110
0.01014 [436]: 12021110
0.00921 [24]: 22110110
0.00801 [25]: 22110101
0.00527 [120]: 21122000
0.00484 [94]: 21212000
0.00435 [155]: 21101111
0.00421 [11]: 22200101
0.00340 [124]: 21120200
0.00277 [191]: 21011102
0.00274 [90]: 21221000
0.00270 [156]: 21101102
0.00263 [98]: 21210200
0.00259 [437]: 12021101

S_{18}

0.18191 [362]: 12211100
0.10129 [388]: 12121100
0.08350 [10]: 22200110
0.05331 [375]: 12201110
0.05114 [24]: 22110110
0.04675 [121]: 21121100
0.04657 [50]: 22020110
0.04520 [11]: 22200101
0.03624 [436]: 12021110
0.03345 [401]: 12111110

0.02915 [95]: 21211100
0.02387 [51]: 22020101
0.01778 [22]: 22111001
0.01715 [8]: 22201001
0.01508 [7]: 22201010
0.01444 [25]: 22110101
0.01150 [402]: 12111101
0.01070 [437]: 12021101
0.01043 [48]: 22021001
0.00961 [47]: 22021010
0.00860 [170]: 21021101
0.00823 [108]: 21201110
0.00810 [109]: 21201101
0.00721 [135]: 21111101
0.00680 [376]: 12201101
0.00644 [134]: 21111110
0.00516 [365]: 12210200
0.00450 [456]: 12011120
0.00406 [169]: 21021110
0.00388 [361]: 12212000
0.00379 [421]: 12101120
0.00308 [457]: 12011111
0.00287 [486]: 11221001
0.00281 [21]: 22111010

S_{19}

0.17228 [29]: 22102100
0.09319 [55]: 22012100
0.08255 [24]: 22110110
0.06908 [92]: 21220010
0.06778 [72]: 22002110
0.06098 [120]: 21122000
0.04373 [10]: 22200110
0.04333 [25]: 22110101
0.04089 [94]: 21212000
0.03002 [50]: 22020110
0.02437 [93]: 21220001
0.02245 [11]: 22200101
0.01681 [146]: 21102200
0.01622 [73]: 22002101
0.01554 [51]: 22020101
0.01342 [166]: 21022010
0.01307 [131]: 21112010
0.01089 [181]: 21012200
0.01056 [113]: 21200210
0.00895 [200]: 21002210
0.00676 [139]: 21110210
0.00609 [132]: 21112001
0.00571 [114]: 21200201
0.00564 [401]: 12111110
0.00560 [362]: 12211100
0.00536 [16]: 22120100
0.00446 [106]: 21202001
0.00445 [2]: 22210100
0.00410 [140]: 21110201
0.00409 [436]: 12021110
0.00397 [58]: 22011200
0.00321 [388]: 12121100
0.00285 [41]: 22100111
0.00284 [105]: 21202010
0.00272 [121]: 21121100
0.00260 [201]: 21002201
0.00257 [485]: 11221010

2^{dimer}, sa-CASSCF(12,12) with four singlet, two triplet and two quintet roots.

Q_1

0.81994 [0]: 222211110000
0.02044 [125]: 222111120000
0.01782 [35]: 222201111000
0.01519 [71]: 222121101000
0.01094 [755]: 221212010100
0.00963 [275]: 222101121000
0.00796 [6049]: 211211110110
0.00707 [17224]: 122111111001
0.00689 [764]: 221211210000
0.00669 [2587]: 220211110200
0.00666 [475]: 222011112000
0.00636 [16]: 222210110100
0.00606 [815]: 221210210100
0.00455 [11201]: 202211110020
0.00438 [39635]: 022211110002

Q_2

0.42715 [1]: 222211101000
0.32876 [70]: 222121110000
0.11538 [126]: 222111111000
0.01280 [471]: 222011121000
0.01196 [279]: 222101112000
0.00579 [758]: 221212001100
0.00447 [1056]: 221122010100
0.00418 [6058]: 211211101110
0.00375 [19]: 222210101100
0.00352 [2596]: 220211101200
0.00322 [6637]: 211121110110
0.00317 [818]: 221210201100
0.00310 [765]: 221211201000
0.00301 [1065]: 221121210000
0.00289 [16735]: 122211110001
0.00271 [2860]: 220121110200

T_1

0.67097 [0]: 222221100000
0.16683 [118]: 222201120000
0.04048 [342]: 222111111000
0.00915 [1473]: 221222000100
0.00653 [8895]: 211221100110
0.00549 [3803]: 220221100200
0.00535 [1476]: 221221200000
0.00526 [8]: 222220100100
0.00510 [767]: 222021102000
0.00500 [24144]: 122121101001
0.00495 [1499]: 221220200100
0.00373 [14937]: 202221100020
0.00362 [49293]: 022221100002
0.00320 [23713]: 122211110001

0.00318 [1]: 222221010000

T_2

0.62682 [22]: 222212010000
0.17344 [48]: 222210210000
0.03793 [1575]: 221211110100
0.01503 [321]: 222112020000
0.01368 [103]: 222202011000
0.01179 [240]: 222122001000
0.00729 [496]: 222102021000
0.00541 [24298]: 122112011001
0.00517 [9049]: 211212010110
0.00508 [906]: 222012012000
0.00461 [3942]: 220212010200
0.00458 [21]: 222212100000
0.00434 [386]: 222110220000
0.00373 [163]: 222200211000
0.00351 [15076]: 202212010020
0.00344 [1548]: 221212110000
0.00337 [6661]: 212211110010
0.00335 [49432]: 022212010002
0.00331 [35]: 222211010100
0.00322 [266]: 222120201000

T_3

0.80150 [28]: 222211110000
0.01988 [341]: 222111120000
0.01739 [119]: 222201111000
0.01509 [246]: 222121101000
0.01093 [1555]: 221212010100
0.00951 [541]: 222101121000
0.00777 [9094]: 211211110110
0.00691 [24343]: 122111111001
0.00666 [1568]: 221211210000
0.00653 [3987]: 220211110200
0.00650 [951]: 222011112000
0.00625 [55]: 222210110100
0.00603 [1620]: 221210210100
0.00485 [27]: 222211200000
0.00467 [33]: 222211020000
0.00445 [15121]: 202211110020
0.00428 [49477]: 022211110002
0.00255 [6]: 222220110000
0.00251 [1]: 222221010000

T_4

0.32642 [23]: 222212001000
0.25211 [239]: 222122010000
0.09046 [49]: 222210201000
0.08836 [322]: 222112011000
0.06906 [265]: 222120210000
0.02437 [387]: 222110211000
0.01990 [1579]: 221211101100
0.01521 [2002]: 221121110100
0.00959 [902]: 222012021000
0.00909 [500]: 222102012000
0.00543 [2205]: 221111111100
0.00397 [21]: 222212100000
0.00272 [1022]: 222010221000
0.00271 [9058]: 211212001110
0.00252 [620]: 222100212000

S_0 0.52333 [0]: 222222000000
0.14253 [7]: 222220200000
0.12785 [111]: 222202020000
0.03592 [176]: 222200220000
0.03145 [1563]: 221221100100
0.03129 [350]: 222112011000
0.00866 [415]: 222110211000
0.00783 [1858]: 221201120100
0.00432 [9085]: 211222000110
0.00397 [781]: 222022002000
0.00390 [24586]: 122122001001
0.00385 [3873]: 220222000200
0.00310 [1554]: 221222100000
0.00294 [15133]: 202222000020
0.00283 [49741]: 022222000002
0.00280 [6775]: 212221100010
0.00275 [4]: 222221000100

S_1

0.79242 [35]: 222211110000
0.01960 [369]: 222111120000
0.01717 [132]: 222201111000
0.01504 [274]: 222121101000
0.01092 [1639]: 221212010100
0.00945 [569]: 222101121000
0.00768 [9304]: 211211110110
0.00721 [34]: 222211200000
0.00692 [40]: 222211020000
0.00683 [24805]: 122111111001
0.00655 [1652]: 221211210000
0.00646 [4092]: 220211110200
0.00643 [1000]: 222011112000
0.00620 [62]: 222210110100
0.00602 [1704]: 221210210100
0.00440 [15352]: 202211110020
0.00424 [49960]: 022211110002
0.00379 [2]: 222221010000
0.00376 [8]: 222220110000
0.00363 [106]: 222202110000

S_2

0.22947 [7]: 222220200000
0.20514 [4]: 222221000100
0.17467 [1554]: 221222100000
0.05260 [176]: 222200220000
0.05102 [147]: 222201020100
0.04524 [1563]: 221221100100
0.04351 [1797]: 221202120000
0.01776 [0]: 222222000000
0.01426 [415]: 222110211000
0.01232 [389]: 222111011100
0.01223 [1858]: 221201120100
0.01165 [22]: 222220000200
0.01066 [2225]: 221112111000
0.00910 [3858]: 220222200000
0.00827 [266]: 222122100000
0.00680 [111]: 222202020000
0.00289 [225]: 222200020200
0.00275 [2289]: 221111111100

S_3

0.21810 [111]: 222202020000
0.19156 [30]: 222212001000

0.15686 [267]: 222122010000
0.06591 [176]: 222200220000
0.05283 [56]: 222210201000
0.04258 [293]: 222120210000
0.04048 [0]: 222222000000
0.02849 [350]: 222112011000
0.02352 [116]: 222202002000
0.02035 [776]: 222022020000
0.01267 [1858]: 221201120100
0.01170 [1663]: 221211101100
0.00946 [2086]: 221121110100
0.00770 [7]: 222220200000
0.00733 [415]: 222110211000
0.00638 [181]: 222200202000
0.00567 [841]: 222020220000
0.00319 [106]: 222202110000
0.00307 [1563]: 221221100100

3$^{\mathbf{dimer}}$, sa-CASSCF(12,12) with four singlet, two triplet and two quintet roots.

Q$_1$

0.87061 [0]: 222211110000
0.01969 [125]: 222111120000
0.01699 [35]: 222201111000
0.01077 [71]: 222121101000
0.00840 [275]: 222101121000
0.00683 [755]: 221212010100
0.00584 [475]: 222011112000
0.00511 [815]: 221210210100
0.00504 [2587]: 220211110200
0.00496 [764]: 221211210000
0.00405 [4092]: 212211110100
0.00333 [16]: 222210110100
0.00313 [720]: 221221010100
0.00292 [106]: 222112101000

Q$_2$

0.44742 [1]: 222211101000
0.27388 [70]: 222121110000
0.11394 [126]: 222111111000
0.05823 [105]: 222112110000
0.01315 [471]: 222011121000
0.01085 [85]: 222120111000
0.00811 [279]: 222101112000
0.00637 [245]: 222102111000
0.00367 [1056]: 221122010100
0.00359 [758]: 221212001100
0.00302 [4090]: 212211120000
0.00271 [818]: 221210201100
0.00268 [2596]: 220211101200

T$_1$

0.53637 [0]: 222221100000
0.14406 [118]: 222201120000
0.14210 [21]: 222212100000
0.05508 [53]: 222210120000
0.03868 [342]: 222111111000
0.00760 [1473]: 221222000100
0.00503 [271]: 222120111000
0.00388 [482]: 222102111000
0.00374 [28]: 222211110000
0.00362 [767]: 222021102000
0.00307 [3803]: 220221100200
0.00305 [1499]: 221220200100
0.00288 [1476]: 221221200000

T$_2$

0.42959 [22]: 222212010000
0.20236 [1]: 222221010000
0.15269 [48]: 222210210000
0.05064 [113]: 222201210000
0.03547 [1575]: 221211110100
0.00966 [321]: 222112020000
0.00964 [240]: 222122001000
0.00653 [103]: 222202011000
0.00555 [34]: 222211011000
0.00520 [1504]: 221220110100
0.00441 [250]: 222121020000
0.00411 [1548]: 221212110000
0.00398 [163]: 222200211000
0.00395 [496]: 222102021000
0.00383 [1715]: 221202110100
0.00370 [1477]: 221221110000
0.00343 [35]: 222211010100
0.00329 [386]: 222110220000
0.00318 [356]: 222111021000
0.00289 [906]: 222012012000
0.00267 [1574]: 221211111000
0.00259 [3942]: 220212010200

T$_3$

0.51907 [28]: 222211110000
0.19382 [6]: 222220110000
0.14507 [98]: 222202110000
0.01225 [119]: 222201111000
0.01134 [341]: 222111120000
0.01042 [246]: 222121101000
0.00757 [541]: 222101121000
0.00693 [1555]: 221212010100
0.00684 [21]: 222212100000
0.00501 [1620]: 221210210100
0.00488 [54]: 222210111000
0.00407 [270]: 222120120000
0.00399 [481]: 222102120000
0.00348 [951]: 222011112000
0.00326 [53]: 222210120000
0.00318 [1568]: 221211210000
0.00311 [317]: 222112101000
0.00299 [3987]: 220211110200
0.00294 [401]: 222110121000
0.00293 [1484]: 221221010100
0.00276 [55]: 222210110100

T$_4$

0.22407 [29]: 222211101000
0.19962 [118]: 222201120000
0.17588 [245]: 222121110000
0.08062 [53]: 222210120000
0.07559 [0]: 222221100000
0.03679 [316]: 222112110000
0.03066 [342]: 222111111000
0.02674 [7]: 222220101000
0.02015 [762]: 222021120000
0.01948 [99]: 222202101000
0.01823 [123]: 222201102000
0.00386 [21]: 222212100000

0.00370 [58]: 222210102000
0.00368 [271]: 222120111000
0.00262 [887]: 222012120000

S_0

0.49134 [0]: 222222000000
0.09685 [7]: 222220200000
0.07326 [111]: 222202020000
0.06275 [40]: 222211020000
0.05809 [34]: 222211200000
0.04780 [176]: 222200220000
0.02693 [1563]: 221221100100
0.02469 [350]: 222112011000
0.01299 [13]: 222220020000
0.00975 [279]: 222121011000
0.00862 [415]: 222110211000
0.00834 [105]: 222202200000
0.00789 [1634]: 221212100100
0.00745 [1858]: 221201120100
0.00576 [1554]: 221222100000
0.00333 [781]: 222022002000
0.00304 [1718]: 221210120100
0.00298 [4]: 222221000100
0.00292 [3873]: 220222000200
0.00273 [555]: 222101211000
0.00255 [2]: 222221010000

S_1

0.34955 [35]: 222211110000
0.28738 [8]: 222220110000
0.21520 [106]: 222202110000
0.01029 [274]: 222121101000
0.01001 [132]: 222201111000
0.00786 [28]: 222212100000
0.00731 [369]: 222111120000
0.00718 [569]: 222101121000
0.00699 [1639]: 221212010100
0.00642 [61]: 222210111000
0.00603 [298]: 222120120000
0.00591 [509]: 222102120000
0.00496 [1704]: 221210210100
0.00377 [60]: 222210120000
0.00359 [1]: 222221100000
0.00322 [429]: 222110121000
0.00320 [345]: 222112101000
0.00282 [1568]: 221221010100
0.00250 [62]: 222210110100

S_2

0.12890 [267]: 222122010000
0.09329 [40]: 222211020000
0.08948 [3]: 222221001000
0.08929 [30]: 222212001000
0.08276 [111]: 222202020000
0.06717 [7]: 222220200000
0.04078 [56]: 222210201000
0.02810 [293]: 222120210000
0.02411 [13]: 222220020000
0.02357 [1554]: 221222100000
0.02217 [34]: 222211200000
0.01703 [1858]: 221201120100
0.01699 [176]: 222200220000
0.01654 [4]: 222221000100
0.01544 [127]: 222201201000
0.01534 [350]: 222112011000
0.01518 [0]: 222222000000
0.01489 [364]: 222111210000
0.01332 [415]: 222110211000
0.01317 [776]: 222022020000
0.01254 [147]: 222201020100
0.01044 [31]: 222212000100
0.01000 [1657]: 221211120000
0.00914 [1663]: 221211101100
0.00754 [1718]: 221210120100
0.00737 [1797]: 221202120000
0.00663 [116]: 222202002000
0.00631 [2086]: 221121110100
0.00586 [45]: 222211002000
0.00550 [181]: 222200202000
0.00489 [76]: 222210020100
0.00425 [279]: 222121011000
0.00390 [555]: 222101211000
0.00359 [841]: 222020220000
0.00278 [1586]: 221220120000

S_3

0.16012 [1554]: 221222100000
0.14119 [4]: 222221000100
0.11537 [176]: 222200220000
0.08219 [7]: 222220200000
0.05329 [34]: 222211200000
0.04459 [30]: 222212001000
0.03748 [1563]: 221221100100
0.03743 [0]: 222222000000
0.03052 [267]: 222122010000
0.02937 [147]: 222201020100
0.02455 [31]: 222212000100
0.01755 [1797]: 221202120000
0.01612 [1657]: 221211120000
0.01417 [111]: 222202020000
0.01108 [76]: 222210020100
0.01053 [389]: 222111011100
0.01004 [1634]: 221212100100
0.00889 [2225]: 221112111000
0.00782 [105]: 222202200000
0.00700 [56]: 222210201000
0.00617 [2]: 222221010000
0.00581 [350]: 222112011000
0.00548 [3858]: 220222200000
0.00519 [415]: 222110211000
0.00511 [40]: 222211020000
0.00405 [293]: 222120210000
0.00392 [2289]: 221111111100
0.00384 [1586]: 221220120000
0.00347 [776]: 222022020000
0.00339 [364]: 222111210000
0.00326 [146]: 222201021000
0.00294 [2086]: 221121110100
0.00282 [279]: 222121011000
0.00279 [2085]: 221121111000

Section S7. Diabatic Electronic States

The diabatic representation of states was calculated in order to obtain their electronic coupling. Particular attention was given to the singlet ground state ($^1|S_0S_0>$), the triplet pair states ($^1|T_1T_1>$), the initially excited singlet states ($^1|S_0S_1>$ and $^1|S_1S_0>$), the further locally excited singlet states ($^1|LE>$), the charge transfer anion−cation ($^1|AC>$) and cation−anion ($^1|CA>$) singlet states and the doubly excited singlet states ($^1|DE>$).

The diabatic wavefunctions (ϕ) are represented as a linear combination of adiabatic wavefunctions (ψ_i) where the expansion coefficients (C_i) are the configuration state function (CSF) coefficients as obtained from the sa-CASSCF calculations.

$$\Phi_i = \sum_i C_i \psi_i \qquad \textbf{Eq.1}$$

The completeness of an adiabatic state (Σ) is the sum of the CSF coefficients used for the construction of the diabatic representation. Based on this definition an adiabatic state is well represented when Σ approaches unity. Note that for some adiabatic states, the Σ was moderate due to high multireference character. The active space of 4e/4o with eight singlet roots provides an excellent completeness, however the energetic ordering of the states is unsatisfactory in reference with the experiment (Table S1). The active space of 8e/8o with eight singlet roots provides a reasonable ordering of the electronic states; unfortunately with insufficient completeness (Table S2). The active space of 8e/8o with twenty singlet roots provides better energetic ordering and as well an improved completeness (Table S3). Increasing the active space to 12e/12o increases the completeness further (Table S4); unfortunately, at tremendous computational cost of the NEVPT2 calculation.

Table S1: Completeness and energies obtained by NEVPT2//sa-CASSCF(4,4) calculation with eight singlet, four triplet and one quintet roots.

	**2**[dimer]			**3**[dimer]		
**Adiabatic state**	Σ	E_{NEVPT2} [eV]	E_{CASSCF} [eV]	Σ	E_{NEVPT2} [eV]	E_{CASSCF} [eV]
**S_0**	0.96	0.00	5.66	0.77	0.0	5.81
**S_1**	0.99	0.88	0.0	0.80	1.35	0.0
**S_2**	0.80	0.99	5.00	0.70	1.36	5.78
**S_3**	0.84	1.01	5.14	0.99	1.56	0.14
**S_4**	0.99	1.87	4.40	0.76	1.84	5.47
**S_5**	0.99	1.95	1.05	0.94	2.35	5.44
**S_6**	1.00	2.15	3.95	1.00	2.65	4.58
**S_7**	0.98	3.00	5.50	0.89	3.02	5.59

Table S2: Completeness and energies obtained by NEVPT2//sa-CASSCF(8,8) calculation with eight singlet, three triplet and one quintet roots.

Adiabatic state	$\mathbf{2}^{\mathbf{dimer}}$			$\mathbf{3}^{\mathbf{dimer}}$		
	Σ	E_{NEVPT2} [eV]	E_{CASSCF} [eV]	Σ	E_{NEVPT2} [eV]	E_{CASSCF} [eV]
$\mathbf{S_0}$	0.81	0.0	0.0	0.76	0.0	0.0
$\mathbf{S_1}$	0.86	1.14	0.82	0.81	0.79	0.82
$\mathbf{S_2}$	0.58	1.74	3.42	0.12	1.59	2.21
$\mathbf{S_3}$	0.56	1.87	3.43	0.17	1.64	2.25
$\mathbf{S_4}$	0.15	1.95	2.21	0.16	1.88	2.98
$\mathbf{S_5}$	0.10	1.96	2.25	0.15	1.92	3.01
$\mathbf{S_6}$	0.17	2.54	2.98	0.25	1.98	3.42
$\mathbf{S_7}$	0.09	2.76	3.01	0.27	2.01	3.43

Table S3: Completeness and energies obtained by NEVPT2//sa-CASSCF(8,8) calculation with twenty singlet, three triplet and one quintet roots.

Adiabatic state	$\mathbf{2}^{\mathbf{dimer}}$			$\mathbf{3}^{\mathbf{dimer}}$		
	Σ	E_{NEVPT2} [eV]	E_{CASSCF} [eV]	Σ	E_{NEVPT2} [eV]	E_{CASSCF} [eV]
$\mathbf{S_0}$	0.81	0.00	0.00	0.70	0.00	0.0
$\mathbf{S_1}$	0.86	1.11	1.26	0.90	0.77	0.82
$\mathbf{S_2}$	0.52	1.58	4.00	0.29	1.57	2.21
$\mathbf{S_3}$	0.51	1.59	4.02	0.40	1.62	3.65
$\mathbf{S_4}$	0.57	1.77	3.69	0.36	1.62	2.24
$\mathbf{S_5}$	0.54	1.90	3.74	0.40	1.63	3.64
$\mathbf{S_6}$	0.28	1.93	2.50	0.30	1.84	2.96
$\mathbf{S_7}$	0.23	1.94	2.52	0.25	1.92	3.00
$\mathbf{S_8}$	0.29	2.49	3.47	0.36	1.96	3.41
$\mathbf{S_9}$	0.46	2.62	4.97	0.32	2.07	3.39
$\mathbf{S_{10}}$	0.38	2.63	4.91	0.36	2.25	4.25
$\mathbf{S_{11}}$	0.20	2.71	3.52	0.48	2.31	4.14
$\mathbf{S_{12}}$	0.21	2.83	4.86	0.38	2.68	4.48
$\mathbf{S_{13}}$	0.32	2.87	4.95	0.35	2.72	4.44
$\mathbf{S_{14}}$	0.15	3.47	5.06	0.21	2.88	3.88

Table S4: Completeness and energies obtained by NEVPT2//sa-CASSCF(12,12) calculation with four singlet, three triplet and two quintet roots.

Adiabatic state	$\mathbf{2}^{\mathbf{dimer}}$			$\mathbf{3}^{\mathbf{dimer}}$		
	Σ	E_{NEVPT2} [eV]	E_{CASSCF} [eV]	Σ	E_{NEVPT2} [eV]	E_{CASSCF} [eV]
$\mathbf{S_0}$	0.87	0.00	0.00	0.84	0.00	0.00
$\mathbf{S_1}$	0.79	1.19	1.05	0.35	0.78	0.78
$\mathbf{S_2}$	0.73	1.87	2.50	0.47	1.62	2.11
$\mathbf{S_3}$	0.68	1.92	2.57	0.64	1.64	2.17

The selected expansion coefficients were orthogonalized by the Gram-Schmidt procedure.

Electronic couplings were calculated via the explicit construction of orthogonal diabatic states expressed as linear combinations of adiabats.[24-26] Then the electronic couplings (V_{ij}) were calculated as follows:[27-29]

$$V_{ij} = \langle \Phi_i | H | \Phi_j \rangle = \sum_{a,b} C_{ia} C_{jb} \langle \psi_i | H | \psi_j \rangle = \sum_{a} C_{ia} C_{ja} \varepsilon_a \qquad \textbf{Eq.2}$$

ε_a are the energies of the adiabatic states obtained from the NEVPT2//sa-CASSCF calculations.

For the **direct mechanism** the electronic coupling between $|^1(T_1T_1)>$ and $|^1(S_1S_0)>$ (or $|^1(T_1T1)>$ and $|^1(S_0S_1)>$, respectively) was considered. Alternatively charge-transfer states could overall mediate the formation of $|^1(T_1T1)>$ (**mediated mechanism**) according to Eq. **3**.

$$V_{eff} = \underbrace{V_{(S_1S_0)(T_1T_1)}}_{\text{direct}} \underbrace{- 2\frac{V_{(S_1S_0)(CA)}V_{(CA)(T_1T_1)} + V_{(S_1S_0)(AC)}V_{(AC)(T_1T_1)}}{\left[\left(E_{CT} - E_{T_1T_1}\right) + \left(E_{CT} - E_{S_1S_0}\right)\right]}}_{\text{mediated}} \qquad \textbf{Eq.3}$$

Table S5: Orthogonalized CSF coefficients for **2**$^{\mathbf{dimer}}$ obtained by NEVPT2//sa-CASSCF(8,8) calculation with twenty singlet, three triplet and one quintet roots at the TZVPP level of theory.

	$\|^1(S_0S_0)>$	$\|^1(T_1T_1)>$	$\|^1(DE)>$	$\|^1(DE)>$	$\|^1(DE)>$	$\|^1(DE)>$	$\|^1(S_1S_0)>$	$\|^1(S_0S_1)>$	$\|^1(AC)>$	$\|^1(CA)>$	$\|^1(AC)>$	$\|^1(CA)>$	$\|^1(CA)>$	$\|^1(AC)>$	$\|^1(AC)>$
S_0	1.00	-0.01	0.01	0.00	0.00	0.00	0.00	0.00	0.00	0.00	-0.02	-0.02	0.00	0.00	0.00
S_1	0.00	0.95	0.02	0.05	0.00	0.00	0.00	0.00	0.00	-0.08	-0.23	-0.20	0.02	-0.01	0.02
S_2	0.00	0.00	0.00	0.00	0.00	0.00	0.75	-0.65	-0.14	0.01	-0.03	0.03	0.00	-0.04	0.02
S_3	0.00	0.00	0.00	0.00	0.00	0.00	0.03	-0.09	0.67	0.06	-0.14	0.16	-0.28	0.16	0.63
S_4	0.00	0.00	0.00	0.00	0.00	0.00	0.65	0.75	0.02	0.01	-0.02	0.02	-0.07	-0.10	0.03
S_5	0.00	0.00	0.00	0.00	0.00	0.00	0.09	-0.08	0.71	-0.10	0.24	-0.27	0.06	-0.17	-0.56
S_6	0.02	0.25	-0.06	-0.21	-0.01	-0.36	0.00	0.00	0.00	-0.07	0.33	0.69	-0.28	0.18	-0.26
S_7	0.02	0.18	-0.26	0.03	-0.15	0.42	0.00	0.00	0.00	0.11	0.66	0.12	0.32	-0.21	0.30
S_8	0.00	0.00	0.00	0.00	0.00	0.00	0.02	0.02	0.19	0.17	-0.42	0.48	0.72	-0.03	-0.11
S_9	0.00	0.08	0.00	-0.10	0.52	-0.06	0.00	0.00	0.00	0.82	0.10	-0.12	-0.07	0.04	-0.06
S_{10}	0.00	0.00	0.09	0.91	0.26	-0.21	0.00	0.00	0.00	-0.07	0.15	0.12	0.03	-0.02	0.03
S_{11}	0.00	0.00	0.00	0.00	0.00	0.00	0.12	0.05	0.02	-0.08	0.19	-0.22	0.30	0.90	-0.01
S_{12}	0.00	0.01	0.66	-0.26	0.14	-0.41	0.00	0.00	0.00	-0.15	0.27	-0.11	0.30	-0.19	0.28
S_{13}	0.00	0.05	0.64	0.18	-0.51	0.32	0.00	0.00	0.00	0.34	0.02	0.12	-0.17	0.11	-0.16
S_{14}	0.00	0.02	0.28	-0.10	0.60	0.62	0.00	0.00	0.00	-0.33	-0.03	0.21	-0.09	0.06	-0.08

Table S6: Orthogonalized CSF coefficients for **3**$^{\mathbf{dimer}}$ obtained by NEVPT2//sa-CASSCF(8,8) calculation with twenty singlet, three triplet and one quintet roots at the TZVPP level of theory.

	$\|^1(S_0S_0)>$	$\|^1(T_1T_1)>$	$\|^1(DE)>$	$\|^1(DE)>$	$\|^1(DE)>$	$\|^1(DE)>$	$\|^1(S_1S_0)>$	$\|^1(S_0S_1)>$	$\|^1(AC)>$	$\|^1(CA)>$	$\|^1(AC)>$	$\|^1(CA)>$	$\|^1(LE)>$	$\|^1(DE)>$	$\|^1(AC)>$
S_0	1.00	0.00	0.00	-0.04	0.05	-0.04	0.00	0.00	0.00	0.00	0.00	0.00	0.00	0.00	0.00
S_1	0.00	0.67	0.03	-0.01	0.09	0.10	-0.01	0.00	0.00	0.00	0.71	-0.15	-0.02	-0.02	0.00
S_2	0.00	0.00	0.29	0.14	0.51	0.42	-0.03	0.00	-0.01	0.01	0.01	0.66	0.17	0.01	0.00
S_3	0.00	0.00	0.00	0.00	0.00	0.00	0.81	0.01	-0.48	-0.15	0.00	-0.03	0.24	-0.21	-0.02
S_4	0.07	0.01	0.03	0.52	-0.65	0.55	0.00	0.00	0.00	0.00	0.00	0.02	0.00	0.00	0.00
S_5	0.00	0.00	0.00	0.00	0.00	0.00	0.21	0.07	0.00	0.96	0.00	0.01	-0.07	0.05	0.13
S_6	0.00	0.00	0.00	0.00	0.00	0.00	0.00	0.64	0.01	0.05	0.00	0.00	-0.02	0.01	-0.77
S_7	0.00	0.00	0.00	0.00	0.06	0.07	0.42	-0.04	0.18	-0.16	0.00	0.09	-0.64	0.58	-0.01

S_8	0.00	0.00	0.00	0.00	0.00	0.00	0.36	-0.02	0.86	-0.04	0.00	-0.03	0.27	-0.24	-0.02
S_9	0.00	0.01	0.00	0.00	0.00	0.00	0.00	0.77	0.03	-0.14	-0.01	0.00	0.00	0.01	0.62
S_{10}	0.00	0.00	0.20	0.79	0.36	-0.32	0.00	0.00	0.00	0.00	-0.06	-0.31	-0.06	-0.02	0.00
S_{11}	0.00	0.00	0.94	-0.23	-0.21	-0.08	0.01	0.00	0.00	0.00	-0.04	-0.13	-0.03	0.01	0.00
S_{12}	0.00	0.27	0.00	0.16	-0.36	-0.60	0.03	-0.01	0.01	-0.01	0.01	0.63	0.00	0.00	0.00
S_{13}	0.00	0.66	-0.03	-0.06	0.08	0.15	-0.01	-0.01	0.00	0.00	-0.68	-0.07	-0.16	-0.18	0.00
S_{14}	0.00	0.19	-0.01	0.00	-0.02	-0.03	0.00	0.00	0.00	0.01	-0.15	-0.13	0.64	0.72	-0.01

Table S7*:* Energies and coupling matrix elements of the low-lying diabatic electronic states of **2**$^{\mathbf{dimer}}$ calculated at the NEVPT2//sa-CASSCF(8,8) with twenty singlet, three triplet and one quintet roots level of theory. The electronic coupling *V* for the direct mechanism is highlighted in yellow, and those for the mediated mechanism are highlighted in green and orange.

**V**$_{\mathbf{ij}}$	$|^1(S_0S_0)>$	$|^1(T_1T_1)>$	$|^1(DE)>$	$|^1(DE)>$	$|^1(DE)>$	$|^1(DE)>$	$|^1(S_1S_0)>$	$|^1(S_0S_1)>$	$|^1(AC)>$	$|^1(CA)>$	$|^1(AC)>$	$|^1(CA)>$	$|^1(CA)>$	$|^1(AC)>$	$|^1(AC)>$
$|^1(S_0S_0)>$	1	16	-10	-8	-5	-3	0	0	0	0	34	33	-3	2	-2
$|^1(T_1T_1)>$	16	1205	32	-45	22	26	0	0	0	107	185	159	-26	16	-24
$|^1(DE)>$	-10	32	2829	-36	54	203	0	0	0	-26	172	109	40	-26	38
$|^1(DE)>$	-8	-45	-36	2627	-76	-77	0	0	0	45	46	109	-64	41	-60
$|^1(DE)>$	-5	22	54	-76	2981	305	0	0	0	-204	63	106	18	-12	17
$|^1(DE)>$	-3	26	203	-77	305	2801	0	0	0	-182	-142	265	-244	157	-227
$|^1(S_1S_0)>$	0	0	0	0	0	0	1682	98	29	-9	23	-26	45	102	-15
$|^1(S_0S_1)>$	0	0	0	0	0	0	98	1697	-11	2	-4	5	19	43	16
$|^1(AC)>$	0	0	0	0	0	0	29	-11	1782	7	-17	19	144	-23	-142
$|^1(CA)>$	0	107	-26	45	-204	-182	-9	2	7	2714	-26	-85	-31	-4	-106
$|^1(AC)>$	34	185	172	46	63	-142	23	-4	-17	-26	2100	-201	-62	99	129
$|^1(CA)>$	33	159	109	109	106	265	-26	5	19	-85	-201	2159	92	-126	-124
$|^1(CA)>$	-3	-26	40	-64	18	-244	45	19	144	-31	-62	92	2381	133	132
$|^1(AC)>$	2	16	-26	41	-12	157	102	43	-23	-4	99	-126	133	2602	-111
$|^1(AC)>$	-2	-24	38	-60	17	-227	-15	16	-142	-106	129	-124	132	-111	1900

In the **2**$^{\mathbf{dimer}}$, no direct coupling between $|^1(T_1T_1)>$ and $|^1(S_1S_0)>$ (or $|^1(T_1T_1)>$ and $|^1(S_0S_1)>$) was obtained (Figure S7, yellow). Instead, a mediated mechanism based on coupling between the $|^1(S_1S_0)>$ and $|^1(S_0S_1)>$ states with the $|^1(AC)>$ and $|^1(CA)>$ states (Table S7, green), which in turn couple with the $|^1(T_1T_1)>$ state, was predicted (Table S7, green). Overall, this leads to V_{eff} =3 meV.

Table S8: Energies and coupling matrix elements of the low-lying diabatic electronic states of **3**$^{\mathbf{dimer}}$ calculated at the NEVPT2//sa-CASSCF(8,8) with twenty singlet, three triplet and one quintet roots level of theory. The electronic coupling *V* for the direct mechanism is highlighted in yellow, and those for the mediated mechanism are highlighted in green and orange.

**V$_{ij}$**	$\|^1(S_0S_0)>$	$\|^1(T_1T_1)>$	$\|^1(DE)>$	$\|^1(DE)>$	$\|^1(DE)>$	$\|^1(DE)>$	$\|^1(S_1S_0)>$	$\|^1(S_0S_1)>$	$\|^1(AC)>$	$\|^1(CA)>$	$\|^1(AC)>$	$\|^1(CA)>$	$\|^1(LE)>$	$\|^1(AC)>$	$\|^1(DE)>$
$\|^1(S_0S_0)>$	9	1	4	62	-77	66	0	0	0	0	1	2	1	0	0
$\|^1(T_1T_1)>$	1	1841	-42	9	-103	-129	7	-9	3	-4	-939	185	49	53	-4
$\|^1(DE)>$	4	-42	2243	-48	-101	-102	6	0	3	-2	-27	-126	-37	-4	0
$\|^1(DE)>$	62	9	-48	2081	142	-267	5	0	2	-2	32	-24	-11	6	0
$\|^1(DE)>$	-77	-103	-101	142	1860	172	-4	0	-1	1	-118	-299	-57	-29	1
$\|^1(DE)>$	66	-129	-102	-267	172	2087	-9	1	-4	3	-156	-340	-57	-41	2
$\|^1(S_1S_0)>$	0	7	6	5	-4	-9	1716	-8	130	-23	8	25	-47	48	-4
$\|^1(S_0S_1)>$	0	-9	0	0	0	1	-8	1972	4	-39	1	-2	0	0	110
$\|^1(AC)>$	0	3	3	2	-1	-4	130	4	1883	-23	3	1	45	-39	1
$\|^1(CA)>$	0	-4	-2	-2	1	3	-23	-39	-23	1650	-4	-13	35	-14	-46
$\|^1(AC)>$	1	-939	-27	32	-118	-156	8	1	3	-4	1740	188	16	15	2
$\|^1(CA)>$	2	185	-126	-24	-299	-340	25	-2	1	-13	188	2107	-99	-87	-1
$\|^1(LE)>$	1	49	-37	-11	-57	-57	-47	0	45	35	16	-99	2304	473	-3
$\|^1(AC)>$	0	53	-4	6	-29	-41	48	0	-39	-14	15	-87	473	2429	-6
$\|^1(DE)>$	0	-4	0	0	1	2	-4	110	1	-46	2	-1	-3	-6	1923

In the **3**$^{\mathbf{dimer}}$, weak direct coupling between $|^1(T_1T_1)>$ and $|^1(S_1S_0)>$ (or $|^1(T_1T_1)>$ and $|^1(S_0S_1)>$) was obtained (Figure S8, yellow). Furthermore, a mediated mechanism including coupling between the $|^1(S_1S_0)>$ and $|^1(S_0S_1)>$ states with the $|^1(AC)>$ and $|^1(CA)>$ states (Table S7, green), which in turn couple with the $|^1(T_1T_1)>$ state was predicted (Table S7, green). Overall, this leads to V_{eff} = 17 meV.

Table S9*:* Spin-orbit coupling matrix elements (in cm^{-1}) obtained from sa-CASSCF(12,12) calculation with four singlet, three triplet and two quintet roots via quasi-degenerate perturbation theory (QDPT) with the NEVPT2 diagonal energies.

2^{dimer}							3^{dimer}						
I(Mult.)	J(Mult.)	I	J	$\langle I \vert L_X S \vert J \rangle$	$\langle I \vert L_Y S \vert J \rangle$	$\langle I \vert L_Z S \vert J \rangle$	I(Mult.)	J(Mult.)	I	J	$\langle I \vert L_X S \vert J \rangle$	$\langle I \vert L_Y S \vert J \rangle$	$\langle I \vert L_Z S \vert J \rangle$
0(5)	0(5)	0	0	0.00	0.00	0.00	0(5)	0(5)	0	0	0.00	0.00	0.00
0(5)	0(5)	1	0	0.08	-0.01	-0.06	0(5)	0(5)	1	0	-0.30	0.00	0.34
0(5)	0(5)	1	1	0.00	0.00	0.00	0(5)	0(5)	1	1	0.00	0.00	0.00
0(5)	1(3)	0	0	-0.06	0.04	0.06	0(5)	1(3)	0	0	0.07	0.00	-0.08
0(5)	1(3)	0	1	0.00	-0.15	-0.01	0(5)	1(3)	0	1	0.00	-0.08	0.01
0(5)	1(3)	0	2	0.00	0.00	0.00	0(5)	1(3)	0	2	0.00	0.00	0.00
0(5)	1(3)	0	3	0.00	0.00	0.00	0(5)	1(3)	0	3	-0.24	-0.01	0.28
0(5)	1(3)	1	0	-0.29	0.08	0.27	0(5)	1(3)	1	0	0.14	-0.04	-0.16
0(5)	1(3)	1	1	-0.01	0.00	0.01	0(5)	1(3)	1	1	-0.01	0.00	0.01
0(5)	1(3)	1	2	-0.04	0.00	0.03	0(5)	1(3)	1	2	0.14	0.00	-0.16
0(5)	1(3)	1	3	0.00	-0.15	-0.01	0(5)	1(3)	1	3	-0.04	0.01	0.04
0(5)	2(1)	0	0	0.00	0.00	0.00	0(5)	2(1)	0	0	0.00	0.00	0.00
0(5)	2(1)	0	1	0.00	0.00	0.00	0(5)	2(1)	0	1	0.00	0.00	0.00
0(5)	2(1)	0	2	0.00	0.00	0.00	0(5)	2(1)	0	2	0.00	0.00	0.00
0(5)	2(1)	0	3	0.00	0.00	0.00	0(5)	2(1)	0	3	0.00	0.00	0.00
0(5)	2(1)	1	0	0.00	0.00	0.00	0(5)	2(1)	1	0	0.00	0.00	0.00
0(5)	2(1)	1	1	0.00	0.00	0.00	0(5)	2(1)	1	1	0.00	0.00	0.00
0(5)	2(1)	1	2	0.00	0.00	0.00	0(5)	2(1)	1	2	0.00	0.00	0.00
0(5)	2(1)	1	3	0.00	0.00	0.00	0(5)	2(1)	1	3	0.00	0.00	0.00
1(3)	1(3)	0	0	0.00	0.00	0.00	1(3)	1(3)	0	0	0.00	0.00	0.00
1(3)	1(3)	1	0	0.00	0.00	0.00	1(3)	1(3)	1	0	0.00	0.00	0.00
1(3)	1(3)	1	1	0.00	0.00	0.00	1(3)	1(3)	1	1	0.00	0.00	0.00
1(3)	1(3)	2	0	-0.06	0.03	0.06	1(3)	1(3)	2	0	0.06	0.00	-0.07
1(3)	1(3)	2	1	-0.01	0.15	0.02	1(3)	1(3)	2	1	-0.01	0.07	-0.01
1(3)	1(3)	2	2	0.00	0.00	0.00	1(3)	1(3)	2	2	0.00	0.00	0.00
1(3)	1(3)	3	0	0.00	-0.01	0.00	1(3)	1(3)	3	0	0.01	0.00	-0.01
1(3)	1(3)	3	1	0.08	-0.01	-0.06	1(3)	1(3)	3	1	0.01	0.00	-0.01
1(3)	1(3)	3	2	0.00	0.00	0.00	1(3)	1(3)	3	2	0.21	0.00	-0.24
1(3)	1(3)	3	3	0.00	0.00	0.00	1(3)	1(3)	3	3	0.00	0.00	0.00
1(3)	2(1)	0	0	-0.01	0.15	0.02	1(3)	2(1)	0	0	0.00	0.07	-0.01
1(3)	2(1)	0	1	-0.04	0.02	0.03	1(3)	2(1)	0	1	0.04	0.00	-0.04
1(3)	2(1)	0	2	0.00	-0.01	0.00	1(3)	2(1)	0	2	0.00	-0.12	0.02
1(3)	2(1)	0	3	0.03	-0.34	-0.06	1(3)	2(1)	0	3	-0.02	0.25	-0.03
1(3)	2(1)	1	0	0.06	-0.03	-0.06	1(3)	2(1)	1	0	-0.06	0.00	0.07
1(3)	2(1)	1	1	0.00	-0.08	-0.01	1(3)	2(1)	1	1	0.00	-0.03	0.01
1(3)	2(1)	1	2	-0.13	0.05	0.11	1(3)	2(1)	1	2	-0.20	0.00	0.22
1(3)	2(1)	1	3	0.01	-0.01	-0.01	1(3)	2(1)	1	3	-0.08	-0.01	0.09
1(3)	2(1)	2	0	0.00	0.00	0.00	1(3)	2(1)	2	0	0.00	0.01	0.00
1(3)	2(1)	2	1	0.00	0.00	0.00	1(3)	2(1)	2	1	0.00	0.00	0.00
1(3)	2(1)	2	2	0.00	0.00	0.00	1(3)	2(1)	2	2	0.00	0.00	0.00
1(3)	2(1)	2	3	0.00	0.00	0.00	1(3)	2(1)	2	3	-0.01	0.03	0.00

1(3)	2(1)	3	0	0.29	-0.08	-0.27	1(3)	2(1)	3	0	0.01	0.02	-0.01
1(3)	2(1)	3	1	0.00	0.00	0.00	1(3)	2(1)	3	1	-0.11	0.00	0.13
1(3)	2(1)	3	2	-0.07	0.01	0.06	1(3)	2(1)	3	2	0.00	-0.06	0.01
1(3)	2(1)	3	3	0.00	0.00	0.00	1(3)	2(1)	3	3	0.00	0.02	0.00

Section S8. XYZ Coordinates

1[monomer]

N	-0.41120	-6.42400	-12.27530
N	-7.31540	-2.29070	-3.29580
C	-4.51780	-5.46380	-7.35300
C	-3.57630	-5.55860	-8.34020
C	-2.95010	-4.40340	-8.90020
C	-2.04690	-4.53790	-9.92550
C	-1.32040	-4.69650	-10.89600
C	3.35220	-2.64100	-11.97780
C	1.84640	-3.26140	-10.14830
C	2.09150	-3.42800	-11.64670
C	-2.96010	-2.04910	-14.40230
C	0.90270	-3.02770	-12.55910
C	0.05570	-1.87170	-11.99610
C	-1.00330	-1.39500	-12.97260
C	-1.90570	-2.50660	-13.38160
C	-1.10000	-3.65880	-13.95380
C	1.51240	-7.62470	-13.27780
C	-0.50420	-7.22660	-14.64040
C	2.16930	-8.41470	-9.82480
C	0.90310	-6.63290	-8.55540
C	1.07820	-7.33950	-9.88590
C	-3.85820	-8.23530	-13.62420
C	-4.29410	-6.35020	-12.05410
C	-3.21340	-7.19550	-12.71940
C	-0.25030	-7.91680	-10.35280
C	-0.83630	-8.92080	-9.58380
C	-2.09650	-9.37080	-9.84010
C	-2.82280	-8.84270	-10.85170
C	-2.31900	-7.84110	-11.67720
C	-0.98910	-7.43460	-11.44830
C	0.05150	-4.21300	-13.03980
C	0.87060	-5.23780	-13.83170
C	0.37220	-6.65210	-13.52190
C	-0.55820	-5.09490	-11.97020
C	-3.34890	-3.14980	-8.36770
C	-4.26590	-3.03690	-7.38350
C	-4.86800	-4.18510	-6.83730
C	-5.77840	-4.08490	-5.80580
C	-6.53350	-3.91680	-4.79110
C	-5.77640	-6.67830	-0.63020
C	-4.75530	-5.81470	-2.66260
C	-5.91860	-5.66080	-1.79590
C	-10.76000	-6.05110	-5.20160
C	-7.33300	-5.82030	-2.42760
C	-7.32470	-6.83180	-3.56590
C	-8.72100	-7.06420	-4.15330
C	-9.36890	-5.78710	-4.62330
C	-9.39630	-4.82200	-3.46360
C	-8.04690	-4.50450	-2.78460
C	-7.26350	-1.38410	-0.96590
C	-9.33420	-1.26490	-2.26430
C	-3.87890	-0.36650	-1.99580
C	-3.50430	-2.26560	-3.61470
C	-4.55440	-1.37880	-2.89600
C	-10.05570	-0.41670	-5.76310
C	-8.70490	-2.11870	-7.00050
C	-8.86950	-1.39110	-5.64710
C	-5.49500	-0.76680	-3.90160
C	-5.01730	0.22790	-4.72860
C	-5.77280	0.74560	-5.75850
C	-7.01940	0.24210	-6.00420
C	-7.56300	-0.77210	-5.21840
C	-6.81170	-1.23050	-4.13500
C	-8.34870	-3.54470	-1.63420
C	-8.06680	-2.10070	-2.03090
C	-7.21550	-3.59750	-3.68180
H	-4.92090	-6.77570	-0.47610
H	-9.32530	-3.70280	-1.60370
H	-4.93550	-6.24630	-7.01580
H	-3.33060	-6.41810	-8.66220
H	3.19900	-1.68890	-11.79930
H	4.09330	-2.96160	-11.42240
H	3.57480	-2.76390	-12.92380
H	1.03400	-3.74770	-9.89350
H	2.61020	-3.61920	-9.64940
H	1.73660	-2.30970	-9.93930
H	2.28690	-4.39400	-11.81300
H	-2.51500	-1.77060	-15.22930
H	-3.57180	-2.79090	-14.59460
H	-3.46580	-1.29550	-14.03310
H	1.32460	-2.65980	-13.38770
H	0.65190	-1.11480	-11.77030
H	-0.38510	-2.17070	-11.16150
H	-0.56270	-1.01720	-13.77520
H	-1.53750	-0.67620	-12.55150
H	-2.38400	-2.83570	-12.56680
H	-1.72110	-4.40270	-14.15670
H	-0.70180	-3.36410	-14.81130
H	1.14920	-8.49240	-13.00470
H	2.03110	-7.73320	-14.10330
H	2.09460	-7.27560	-12.57130
H	-1.24180	-6.60880	-14.82650
H	0.03480	-7.34540	-15.44910
H	-0.86620	-8.09310	-14.35810
H	1.89080	-9.12950	-9.21450
H	2.31160	-8.78660	-10.72050
H	3.00320	-8.01560	-9.50140
H	0.73520	-7.29530	-7.85350
H	1.71650	-6.12980	-8.34330
H	0.14280	-6.01650	-8.61030
H	1.36420	-6.65580	-10.55570
H	-4.41670	-8.83410	-13.08710
H	-4.41310	-7.78550	-14.29550
H	-3.15910	-8.75540	-14.07280
H	-3.87360	-5.66190	-11.49720
H	-4.84410	-5.92080	-12.74380
H	-4.85920	-6.92290	-11.49560
H	-2.65050	-6.59420	-13.28700
H	-0.34550	-9.30100	-8.86360
H	-2.46790	-10.06190	-9.30300
H	-3.70340	-9.16410	-11.00280
H	1.82700	-5.16070	-13.58910
H	0.78210	-5.05820	-14.80210
H	-2.95420	-2.35940	-8.71710
H	-4.50720	-2.17760	-7.05850
H	-6.33590	-6.39530	0.12210
H	-6.06240	-7.56490	-0.93530
H	-3.93860	-5.65110	-2.14530
H	-4.73610	-6.72450	-3.02570
H	-4.80990	-5.17030	-3.39960
H	-5.87320	-4.74490	-1.39610
H	-10.70540	-6.76520	-5.86990
H	-11.36640	-6.32310	-4.48140
H	-11.09910	-5.23420	-5.62270
H	-7.89380	-6.23670	-1.71350
H	-6.72160	-6.50850	-4.28150
H	-6.96600	-7.69140	-3.23170
H	-9.29550	-7.48550	-3.46520
H	-8.65130	-7.69160	-4.91620
H	-8.79900	-5.39300	-5.34500

H	-9.78750	-3.97050	-3.78250
H	-10.00760	-5.18680	-2.77550
H	-7.01390	-0.49360	-1.28930
H	-7.80340	-1.29750	-0.15260
H	-6.45250	-1.89790	-0.76600
H	-9.91650	-1.72440	-2.90370
H	-9.81080	-1.14840	-1.41440
H	-9.08510	-0.38660	-2.62140
H	-4.55450	0.22240	-1.60060
H	-3.39510	-0.83430	-1.28320
H	-3.24760	0.16810	-2.52220
H	-2.94590	-1.70710	-4.19300
H	-2.94050	-2.71010	-2.94640
H	-3.96310	-2.94120	-4.15480
H	-5.09830	-1.97870	-2.30860
H	-9.84210	0.28080	-6.41920
H	-10.85250	-0.90550	-6.05600
H	-10.22720	-0.00430	-4.89180
H	-7.98460	-2.78000	-6.93030
H	-9.54220	-2.57180	-7.23090
H	-8.48270	-1.46670	-7.69630
H	-9.11330	-2.07990	-4.96350
H	-4.14170	0.56770	-4.58360
H	-5.42940	1.44970	-6.29710
H	-7.52750	0.59170	-6.72590
H	-7.79180	-3.78590	-0.85140

2^{monomer}

N	2.87614	-6.44746	-14.54856
N	-5.13434	-1.21264	-4.15629
C	-1.33169	-5.75843	-9.99015
C	-0.45086	-5.70506	-11.02565
C	0.10138	-4.43941	-11.48268
C	1.02402	-4.45806	-12.49607
C	1.89765	-4.62517	-13.38069
C	6.88478	-3.72674	-12.84016
C	4.72521	-3.66976	-11.55401
C	5.38254	-4.03165	-12.89239
C	2.06958	-1.62275	-17.20087
C	4.73786	-3.37013	-14.15413
C	3.96937	-2.07827	-13.83746
C	3.45552	-1.38290	-15.09475
C	2.55645	-2.30473	-15.92200
C	3.31372	-3.59125	-16.24744
C	4.73588	-8.02802	-15.09609
C	3.31754	-7.12267	-16.92047
C	4.38053	-8.89598	-11.63269
C	3.16051	-6.89235	-10.68398
C	3.52825	-7.66269	-11.96390
C	-0.26884	-7.58052	-16.93257
C	-0.94264	-5.77209	-15.30189
C	0.18496	-6.72037	-15.74476
C	2.27476	-8.00934	-12.75446
C	1.36244	-8.92326	-12.21125
C	0.13906	-9.17013	-12.82804
C	-0.20788	-8.47536	-13.98371
C	0.67163	-7.55332	-14.56687
C	1.94406	-7.36780	-13.97289
C	3.90065	-4.35412	-15.03004
C	4.77933	-5.52439	-15.55067
C	3.92025	-6.80884	-15.53983
C	2.81827	-5.09503	-14.25533
C	-0.35000	-3.25103	-10.82623
C	-1.26277	-3.29200	-9.78951
C	-3.91499	-3.37228	-6.48192
C	-4.54881	-3.13641	-5.42439
C	-2.91957	-4.39209	-0.80634
C	-2.31380	-4.57423	-3.23874
C	-3.33030	-4.01342	-2.23475
C	-8.72139	-5.48558	-4.26076
C	-4.81874	-4.41450	-2.49527
C	-4.96826	-5.72417	-3.28307
C	-6.42755	-6.15178	-3.40690
C	-7.25307	-5.08083	-4.12179
C	-7.13279	-3.76522	-3.35382
C	-5.69053	-3.25876	-3.07956
C	-4.97831	0.34229	-2.19688
C	-7.20087	-0.22952	-3.13565
C	-1.61688	0.78973	-3.38019
C	-1.31702	-1.28127	-4.80756
C	-2.33112	-0.34858	-4.12263
C	-8.01631	0.36675	-6.61491
C	-6.86270	-1.60671	-7.69001
C	-6.88716	-0.66446	-6.47480
C	-3.34801	0.15382	-5.13725
C	-2.92561	1.04587	-6.13145
C	-3.77115	1.40924	-7.17570
C	-5.04905	0.86296	-7.25609
C	-5.52454	-0.01638	-6.27508
C	-4.67776	-0.33075	-5.18473
C	-5.76298	-2.07289	-2.07920
C	-5.77724	-0.76379	-2.89526
C	-5.08882	-2.58996	-4.30909
H	-1.87632	-4.10365	-0.59728
H	-6.63977	-2.15202	-1.42141
H	-1.73539	-6.72121	-9.66591
H	-0.13521	-6.61719	-11.53372
H	7.06125	-2.63870	-12.77897
H	7.35961	-4.18973	-11.95965
H	7.40511	-4.09598	-13.73942
H	3.64713	-3.87325	-11.55460
H	5.17736	-4.26562	-10.74521
H	4.87417	-2.60849	-11.29957
H	5.27613	-5.12376	-12.99652
H	2.92392	-1.35756	-17.84736
H	1.40330	-2.28033	-17.78269
H	1.51636	-0.69820	-16.97720
H	5.57050	-3.07246	-14.81873
H	4.62700	-1.39571	-13.27489
H	3.11724	-2.30508	-13.17927
H	4.30876	-1.06209	-15.72217
H	2.90966	-0.46404	-14.82455
H	1.68220	-2.57627	-15.30366
H	2.66576	-4.26813	-16.82538
H	4.15925	-3.33099	-16.91100
H	4.09223	-8.91234	-14.97412
H	5.49239	-8.26181	-15.86099
H	5.25882	-7.84349	-14.14942
H	2.74218	-6.27950	-17.32324
H	4.12345	-7.35588	-17.63367
H	2.65421	-7.99732	-16.86230
H	3.85323	-9.57512	-10.94352
H	4.63751	-9.47112	-12.53473
H	5.31881	-8.59358	-11.13973
H	2.57146	-7.51979	-9.99596
H	4.07238	-6.57317	-10.15410
H	2.56561	-5.99857	-10.91319
H	4.13087	-6.98581	-12.58265

H	-1.14850	-8.19076	-16.67178
H	-0.55410	-6.94170	-17.78433
H	0.52242	-8.26653	-17.27004
H	-0.62945	-5.15761	-14.44739
H	-1.22863	-5.09734	-16.12582
H	-1.84328	-6.33471	-15.00683
H	1.02048	-6.09244	-16.07530
H	1.60073	-9.42816	-11.27249
H	-0.56456	-9.87514	-12.38799
H	-1.19285	-8.63633	-14.42806
H	5.64261	-5.67084	-14.88832
H	5.17606	-5.31293	-16.55350
H	0.00807	-2.28261	-11.17536
H	-3.56444	-3.90441	-0.05666
H	-2.99811	-5.48239	-0.65237
H	-1.31782	-4.14881	-3.03610
H	-2.22347	-5.66907	-3.15853
H	-2.57452	-4.32878	-4.27560
H	-3.26270	-2.91584	-2.31345
H	-8.82547	-6.45724	-4.76662
H	-9.19469	-5.57748	-3.26808
H	-9.29434	-4.73856	-4.83451
H	-5.27457	-4.60818	-1.50622
H	-4.54011	-5.60821	-4.29109
H	-4.38425	-6.51455	-2.78380
H	-6.85630	-6.32261	-2.40115
H	-6.50536	-7.11352	-3.93812
H	-6.82541	-4.93475	-5.13108
H	-7.70375	-2.97986	-3.87187
H	-7.62241	-3.90660	-2.37270
H	-4.88924	1.23265	-2.83729
H	-5.49933	0.63896	-1.27350
H	-3.97014	0.00792	-1.92278
H	-7.85118	-0.97540	-3.60880
H	-7.65743	0.05951	-2.17631
H	-7.17363	0.66142	-3.77909
H	-2.32954	1.47979	-2.90351
H	-0.95744	0.38208	-2.59685
H	-0.98493	1.38226	-4.06137
H	-0.73249	-0.74308	-5.57118
H	-0.61203	-1.69261	-4.06671
H	-1.82103	-2.12067	-5.30453
H	-2.87186	-0.95349	-3.38368
H	-7.89075	0.97698	-7.52402
H	-8.99226	-0.13987	-6.69215
H	-8.05489	1.05354	-5.75611
H	-6.04600	-2.33568	-7.60743
H	-7.81459	-2.15712	-7.77151
H	-6.72376	-1.04347	-8.62732
H	-7.09604	-1.28227	-5.59354
H	-1.90498	1.43370	-6.10722
H	-3.42235	2.08413	-7.95630
H	-5.68147	1.10548	-8.11059
H	-4.87387	-2.07389	-1.43459
C	-1.75851	-2.09558	-9.15571
C	-1.77759	-4.56729	-9.30707
H	-1.41647	-1.12817	-9.53119
C	-2.64426	-2.14691	-8.12636
C	-2.66091	-4.60926	-8.24348
H	-3.01081	-1.22910	-7.66864
H	-3.03565	-5.57271	-7.89923
C	-3.11193	-3.41599	-7.59332

3[monomer]

N	2.44973	-6.57061	-15.94689
N	-7.03043	-2.45516	-3.73110
C	-1.43407	-5.76576	-10.99756
C	-0.63842	-5.77491	-12.10913
C	-0.11276	-4.55047	-12.65847
C	0.72353	-4.60635	-13.73857
C	1.48854	-4.79870	-14.70600
C	6.48926	-3.50404	-15.25965
C	4.70318	-3.88800	-13.53837
C	5.06748	-4.02541	-15.02169
C	0.43016	-1.81151	-18.01716
C	4.05725	-3.37205	-16.01693
C	3.30639	-2.17256	-15.41713
C	2.37602	-1.49530	-16.42214
C	1.36961	-2.48433	-17.01787
C	2.12587	-3.65413	-17.65087
C	4.53349	-7.77388	-16.56813
C	2.65714	-7.60661	-18.21145
C	4.57989	-8.55402	-13.05995
C	3.12318	-6.77283	-12.01906
C	3.50453	-7.49223	-13.32813
C	-0.85490	-8.15999	-17.82003
C	-1.44184	-6.32880	-16.17784
C	-0.31172	-7.20070	-16.75292
C	2.25337	-8.05922	-13.98965
C	1.53305	-9.05213	-13.31060
C	0.29969	-9.49897	-13.77765
C	-0.26255	-8.91320	-14.90977
C	0.41849	-7.91956	-15.62555
C	1.71572	-7.54633	-15.19560
C	3.09280	-4.39949	-16.68990
C	3.87772	-5.48179	-17.48640
C	3.37917	-6.88985	-17.05942
C	2.29938	-5.22729	-15.69891
C	-0.49245	-3.32770	-12.00879
C	-1.29122	-3.33849	-10.89774
C	-1.79318	-4.55299	-10.31390
C	-5.14545	-4.48814	-5.73672
C	-5.98071	-4.26609	-4.83582
C	-6.23291	-5.87017	0.10523
C	-4.62467	-5.49677	-1.78514
C	-6.05720	-5.25758	-1.29017
C	-9.66718	-6.84908	-5.74286
C	-7.18995	-5.74142	-2.25095
C	-6.77047	-6.92475	-3.13648
C	-7.90913	-7.44375	-4.01416
C	-8.51523	-6.33564	-4.88118
C	-8.96174	-5.17975	-3.98429
C	-7.84692	-4.59309	-3.07746
C	-7.43985	-1.22169	-1.60845
C	-9.23995	-1.36285	-3.33770
C	-3.87870	-0.64958	-1.81754
C	-3.11167	-2.51451	-3.33654
C	-4.31958	-1.68682	-2.85919
C	-9.05671	-0.69673	-6.87560
C	-7.58092	-2.60002	-7.63335
C	-8.01197	-1.73230	-6.43588
C	-5.02658	-1.06811	-4.05965
C	-4.34012	-0.11135	-4.82001
C	-4.86470	0.37137	-6.01629
C	-6.06493	-0.14348	-6.50049
C	-6.79233	-1.09807	-5.77638
C	-6.29367	-1.50766	-4.51441
C	-8.45438	-3.49623	-2.15891
C	-8.04726	-2.10167	-2.70868
C	-6.86743	-3.80617	-3.92571
H	-5.46594	-5.50339	0.80669

H	-9.54822	-3.59142	-2.11477
H	-1.81174	-6.71987	-10.63005
H	-0.38332	-6.71389	-12.60458
H	6.54714	-2.41739	-15.07281
H	7.21223	-3.99141	-14.58669
H	6.81806	-3.68574	-16.29653
H	3.67603	-4.21099	-13.32793
H	5.37833	-4.50693	-12.92705
H	4.81400	-2.84856	-13.19165
H	5.08316	-5.10586	-15.23691
H	0.99442	-1.36639	-18.85497
H	-0.29285	-2.52731	-18.44224
H	-0.14185	-1.00393	-17.53152
H	4.64966	-2.97684	-16.86339
H	4.04028	-1.44460	-15.03454
H	2.71268	-2.50190	-14.55220
H	2.96967	-1.05340	-17.24540
H	1.84326	-0.65935	-15.93750
H	0.75927	-2.88587	-16.19219
H	1.41292	-4.38054	-18.07796
H	2.72661	-3.27043	-18.49716
H	4.15620	-8.71901	-16.15130
H	5.20754	-8.01524	-17.40461
H	5.11993	-7.26295	-15.79323
H	1.86731	-6.97975	-18.64746
H	3.37904	-7.84656	-19.00633
H	2.20421	-8.54752	-17.86724
H	4.22416	-9.31823	-12.35065
H	4.88895	-9.06916	-13.98152
H	5.47161	-8.08344	-12.61378
H	2.66884	-7.47247	-11.29948
H	4.01584	-6.33513	-11.54450
H	2.40132	-5.96560	-12.20010
H	3.93216	-6.73962	-14.00289
H	-1.60941	-8.84465	-17.40040
H	-1.34150	-7.59650	-18.63244
H	-0.05576	-8.77318	-18.26264
H	-1.05964	-5.62334	-15.42691
H	-1.92814	-5.75147	-16.98186
H	-2.21449	-6.95003	-15.69676
H	0.40234	-6.52296	-17.23641
H	1.93775	-9.46335	-12.38363
H	-0.24499	-10.27502	-13.23384
H	-1.25792	-9.22030	-15.23677
H	4.95600	-5.39512	-17.29905
H	3.73103	-5.34056	-18.56641
H	-0.11595	-2.38473	-12.41297
H	-1.54745	-2.38195	-10.44275
H	-7.22168	-5.63098	0.53116
H	-6.14283	-6.96965	0.06464
H	-3.91156	-4.98365	-1.12103
H	-4.36608	-6.56765	-1.77406
H	-4.46465	-5.11782	-2.80277
H	-6.16740	-4.16628	-1.18125
H	-9.32348	-7.63629	-6.43350
H	-10.46880	-7.27803	-5.11702
H	-10.11137	-6.04090	-6.34731
H	-8.01460	-6.10815	-1.61086
H	-5.93429	-6.62234	-3.78275
H	-6.39252	-7.73786	-2.49516
H	-8.70833	-7.87105	-3.37903
H	-7.54676	-8.26667	-4.65319
H	-7.72730	-5.95909	-5.55463
H	-9.38930	-4.36837	-4.59743
H	-9.77582	-5.53512	-3.32446
H	-7.04459	-0.28271	-2.02346
H	-8.21055	-0.96810	-0.86460
H	-6.62341	-1.74255	-1.09087
H	-9.75765	-1.98574	-4.07936
H	-9.96489	-1.09227	-2.55522
H	-8.91028	-0.43648	-3.82987
H	-4.72987	-0.06862	-1.43309
H	-3.39146	-1.14943	-0.96428
H	-3.15002	0.06100	-2.23989
H	-2.35585	-1.87076	-3.81431
H	-2.63221	-3.02157	-2.48418
H	-3.41353	-3.27762	-4.06675
H	-5.01899	-2.38027	-2.37485
H	-8.65199	-0.01749	-7.64288
H	-9.93337	-1.19903	-7.31585
H	-9.40337	-0.08089	-6.03242
H	-6.85655	-3.36898	-7.33525
H	-8.45712	-3.10049	-8.07834
H	-7.11228	-1.98500	-8.41830
H	-8.48203	-2.40010	-5.70374
H	-3.36644	0.24396	-4.47703
H	-4.31483	1.11793	-6.59527
H	-6.43834	0.18786	-7.47142
H	-8.08595	-3.60858	-1.13148
C	-2.59518	-4.55433	-9.13519
C	-2.98241	-3.33895	-8.47203
C	-3.07248	-5.77004	-8.53319
H	-2.63750	-2.38110	-8.86102
H	-2.78539	-6.72757	-8.96772
C	-3.78051	-3.32960	-7.36276
C	-3.87531	-5.78040	-7.42444
H	-4.06798	-2.38817	-6.89079
H	-4.22858	-6.72484	-7.00302
C	-4.28553	-4.55551	-6.79789

Truncated $2^{monomer}$

N	7.28844	0.52561	-4.36207
C	3.12254	0.31923	1.93740
H	4.19738	0.40657	2.11240
C	2.24575	0.32779	3.04912
N	-2.06853	0.13927	6.22616
C	0.40421	0.10187	1.48841
H	-0.52719	0.00936	0.92960
C	5.04987	0.36303	-3.52834
C	-0.74581	0.30735	5.99490
C	0.86863	0.19289	2.77381
C	0.01942	0.54193	7.28037
C	8.07243	0.59201	-5.64119
C	5.93719	0.43944	-4.56147
C	0.34921	0.20052	3.65998
C	5.11241	-1.09420	-6.37148
H	4.95921	-1.20114	-7.45726
H	5.85163	-1.84395	-6.04969
C	-0.18174	0.22875	4.74674
C	2.38099	0.17857	0.57414
C	4.20172	0.29823	-2.64023
C	3.42099	0.22381	-0.32046
H	4.44955	0.28929	0.04211
C	1.03017	0.09136	0.16086
C	6.98151	0.56239	-6.69667
H	7.18519	-0.21168	-7.44975
H	6.97690	1.51760	-7.24416
C	8.89171	1.86843	-5.70643
H	8.24457	2.75362	-5.59355
H	9.40591	1.94545	-6.67783
H	9.65884	1.90534	-4.91661
C	5.60178	0.34293	-6.03386
C	3.18295	0.20834	-1.70935

C	1.83463	0.11917	-2.13580
H	1.62631	0.10450	-3.20821
C	-2.44128	0.27049	7.68326
C	4.54757	1.36370	-6.57070
H	4.41651	1.21999	-7.65416
C	0.81135	0.07140	-1.24239
H	-0.20529	0.01390	-1.63190
C	9.02293	-0.59303	-5.78825
H	9.79123	-0.60478	-4.99868
H	9.54140	-0.54107	-6.75834
H	8.47319	-1.54664	-5.74357
C	-1.13414	0.79859	8.28314
H	-0.92814	0.33975	9.26163
H	-1.23133	1.88129	8.45467
C	0.99809	1.73699	7.26620
C	-3.55340	1.28692	7.87079
H	-3.29858	2.24281	7.38807
H	-3.71268	1.47488	8.94453
H	-4.51014	0.93382	7.45242
C	0.75565	-0.73768	7.66808
C	-2.88643	-1.05865	8.26399
H	-3.77840	-1.44171	7.74254
H	-3.14111	-0.94588	9.32954
H	-2.09773	-1.82133	8.18089
H	1.43256	1.87796	8.26821
H	1.81765	1.56261	6.55162
H	0.48097	2.66271	6.96999
H	1.26107	-0.61524	8.64087
H	0.06857	-1.59224	7.75049
H	1.51941	-0.99595	6.91760
H	3.57603	1.21631	-6.07480
H	4.87977	2.39695	-6.38384
H	4.16205	-1.31577	-5.86140
C	7.89221	0.55981	-3.02222
H	8.97347	0.38936	-3.10521
H	7.71283	1.52873	-2.52264
H	7.45171	-0.22917	-2.39235
C	-2.99533	-0.18148	5.13114
H	-4.02061	-0.22503	5.52132
H	-2.94341	0.59301	4.34869
H	-2.73771	-1.14830	4.66481
H	2.61344	0.43253	4.05919

Truncated 3^{monomer}

N	2.15147	-0.08821	-8.19430
C	0.82877	0.13545	3.02453
H	1.67096	0.22377	3.71480
C	-0.48449	0.08317	3.55119
N	-2.13237	0.57591	8.11841
C	0.00086	0.00006	0.72066
C	-1.29809	-0.08411	1.27342
H	-2.15989	-0.17828	0.61253
C	0.96414	-0.09938	-6.11515
C	1.06167	0.08986	1.66342
H	2.09567	0.14296	1.32227
C	-0.95217	0.58860	7.45638
C	-1.53740	-0.05053	2.62169
H	-2.56419	-0.11291	2.99065
C	0.21088	0.83215	8.39502
C	1.93511	-0.11029	-9.68020
C	0.98486	-0.10823	-7.47898
C	-0.70337	0.20413	4.91618
C	-0.83629	-1.65897	-8.24517
H	-1.65145	-1.79638	-8.97350
H	-0.07662	-2.43560	-8.42377
C	-0.84081	0.36398	6.10754
C	1.54010	0.00824	-1.25264
H	2.40654	0.05261	-0.59264
C	0.88080	-0.08627	-4.88829
C	1.76619	-0.03500	-2.60579
H	2.78924	-0.01231	-2.98859
C	0.23678	-0.02052	-0.70159
C	0.42097	-0.11870	-9.79128
H	0.08050	-0.92920	-10.45081
H	0.08445	0.81508	-10.26771
C	2.54595	1.11675	-10.33285
H	2.13794	2.03915	-9.88809
H	2.31980	1.13091	-11.41109
H	3.64148	1.14043	-10.21923
C	-0.21764	-0.23925	-8.38814
C	0.69342	-0.08742	-3.51811
C	-0.61644	-0.11875	-2.97864
H	-1.46363	-0.16061	-3.66725
C	-1.99063	0.84752	9.59673
C	-1.35182	0.81715	-8.19021
H	-2.14919	0.64194	-8.92862
C	-0.83051	-0.07897	-1.63693
H	-1.86151	-0.09531	-1.28290
C	2.54852	-1.34955	-10.32625
H	3.64388	-1.37451	-10.21053
H	2.32602	-1.36058	-11.40466
H	2.13741	-2.26979	-9.88158
C	-0.52280	1.27754	9.68554
H	-0.03957	0.86981	10.58590
H	-0.47596	2.37386	9.76930
C	1.21663	1.91481	7.94501
C	-2.89811	1.98492	10.03023
H	-2.74635	2.87269	9.39737
H	-2.67825	2.26360	11.07312
H	-3.96310	1.70480	9.98133
C	0.93547	-0.48667	8.65078
C	-2.31705	-0.38381	10.42093
H	-3.35751	-0.70765	10.25712
H	-2.19530	-0.17036	11.49451
H	-1.66010	-1.22843	10.16465
H	1.96767	2.07998	8.73324
H	1.73777	1.60613	7.02531
H	0.70181	2.86618	7.74017
H	1.74218	-0.34933	9.39046
H	0.25220	-1.25709	9.03647
H	1.38592	-0.87529	7.72379
H	-1.78268	0.74044	-7.18025
H	-0.95791	1.83695	-8.32331
H	-1.24317	-1.81034	-7.23324
C	3.47391	-0.03866	-7.55430
H	4.24760	-0.27025	-8.29783
H	3.67426	0.95585	-7.11685
H	3.52462	-0.77953	-6.74078
C	-3.39081	0.27710	7.41988
H	-4.22926	0.36816	8.12277
H	-3.54280	0.98493	6.58884
H	-3.37565	-0.74172	6.99521

2^{dimer}, singlet

N	2.87446	-6.44698	-14.54940

N	-5.13654	-1.21585	-4.15691
C	-1.32991	-5.75872	-9.98974
C	-0.44917	-5.70476	-11.02526
C	0.10203	-4.43873	-11.48246
C	1.02369	-4.45690	-12.49677
C	1.89668	-4.62394	-13.38204
C	6.88168	-3.72096	-12.84273
C	4.72059	-3.66741	-11.55882
C	5.38002	-4.02853	-12.89637
C	2.06567	-1.62583	-17.20724
C	4.73539	-3.36863	-14.15896
C	3.96687	-2.07634	-13.84401
C	3.45302	-1.38289	-15.10236
C	2.55330	-2.30582	-15.92761
C	3.31037	-3.59284	-16.25151
C	4.73422	-8.02778	-15.09623
C	3.31513	-7.12415	-16.92091
C	4.38453	-8.88937	-11.63290
C	3.16176	-6.88782	-10.68340
C	3.52961	-7.65775	-11.96353
C	-0.27108	-7.58739	-16.92966
C	-0.94538	-5.77686	-15.30152
C	0.18278	-6.72480	-15.74365
C	2.27597	-8.00692	-12.75274
C	1.36511	-8.92118	-12.20771
C	0.14145	-9.17009	-12.82313
C	-0.20707	-8.47736	-13.97954
C	0.67111	-7.55532	-14.56474
C	1.94361	-7.36729	-13.97172
C	3.89821	-4.35389	-15.03338
C	4.77711	-5.52449	-15.55289
C	3.91826	-6.80906	-15.54074
C	2.81651	-5.09429	-14.25725
C	-0.34992	-3.25064	-10.82587
C	-1.26276	-3.29218	-9.78922
C	-3.91586	-3.37454	-6.48220
C	-4.55004	-3.13941	-5.42470
C	-2.92534	-4.38715	-0.80072
C	-2.31309	-4.58209	-3.23032
C	-3.33199	-4.01537	-2.23210
C	-8.72157	-5.49210	-4.25890
C	-4.81996	-4.41701	-2.49450
C	-4.96800	-5.72696	-3.28206
C	-6.42686	-6.15599	-3.40597
C	-7.25353	-5.08596	-4.12097
C	-7.13405	-3.76970	-3.35395
C	-5.69219	-3.26200	-3.07980
C	-4.98354	0.34022	-2.19802
C	-7.20474	-0.23423	-3.13834
C	-1.62444	0.78901	-3.37542
C	-1.31508	-1.27735	-4.80729
C	-2.33358	-0.34989	-4.12180
C	-8.01764	0.36292	-6.61664
C	-6.86438	-1.61165	-7.69017
C	-6.88868	-0.66840	-6.47578
C	-3.35033	0.15149	-5.13707
C	-2.92808	1.04405	-6.13087
C	-3.77310	1.40639	-7.17588
C	-5.05047	0.85902	-7.25718
C	-5.52609	-0.02025	-6.27615
C	-4.67973	-0.33400	-5.18531
C	-5.76590	-2.07574	-2.07996
C	-5.78076	-0.76700	-2.89656
C	-5.09055	-2.59320	-4.30950
H	-1.88245	-4.09840	-0.59033
H	-6.64289	-2.15522	-1.42249
H	-1.73315	-6.72174	-9.66564
H	-0.13337	-6.61662	-11.53369
H	7.05617	-2.63259	-12.78173
H	7.35644	-4.18280	-11.96158
H	7.40357	-4.08959	-13.74133
H	3.64283	-3.87270	-11.56064
H	5.17278	-4.26236	-10.74937
H	4.86751	-2.60585	-11.30447
H	5.27565	-5.12087	-13.00025
H	2.91961	-1.36152	-17.85464
H	1.39909	-2.28433	-17.78767
H	1.51251	-0.70098	-16.98460
H	5.56801	-3.07184	-14.82398
H	4.62456	-1.39299	-13.28248
H	3.11477	-2.30216	-13.18542
H	4.30635	-1.06395	-15.73064
H	2.90813	-0.46294	-14.83389
H	1.67934	-2.57632	-15.30842
H	2.66204	-4.27062	-16.82798
H	4.15543	-3.33351	-16.91604
H	4.09070	-8.91202	-14.97305
H	5.49034	-8.26225	-15.86130
H	5.25765	-7.84230	-14.15002
H	2.73961	-6.28135	-17.32425
H	4.12085	-7.35794	-17.63414
H	2.65186	-7.99878	-16.86178
H	3.85924	-9.56927	-10.94297
H	4.64163	-9.46428	-12.53506
H	5.32277	-8.58510	-11.14102
H	2.57453	-7.51620	-9.99468
H	4.07359	-6.56690	-10.15451
H	2.56500	-5.99516	-10.91217
H	4.13033	-6.97987	-12.58304
H	-1.15005	-8.19792	-16.66723
H	-0.55743	-6.95029	-17.78235
H	0.52053	-8.27333	-17.26646
H	-0.63180	-5.16010	-14.44881
H	-1.23319	-5.10431	-16.12660
H	-1.84497	-6.33988	-15.00401
H	1.01747	-6.09662	-16.07585
H	1.60454	-9.42437	-11.26833
H	-0.56097	-9.87524	-12.38139
H	-1.19225	-8.63991	-14.42284
H	5.64040	-5.67015	-14.89038
H	5.17382	-5.31394	-16.55592
H	0.00812	-2.28215	-11.17484
H	-3.57186	-3.89526	-0.05523
H	-3.00509	-5.47661	-0.64148
H	-1.31739	-4.15631	-3.02711
H	-2.22375	-5.67656	-3.14413
H	-2.57072	-4.34189	-4.26912
H	-3.26345	-2.91826	-2.31620
H	-8.82511	-6.46391	-4.76459
H	-9.19402	-5.58430	-3.26584
H	-9.29564	-4.74568	-4.83231
H	-5.27676	-4.61085	-1.50593
H	-4.53975	-5.61078	-4.29002
H	-4.38332	-6.51668	-2.78252
H	-6.85547	-6.32715	-2.40021
H	-6.50375	-7.11785	-3.93715
H	-6.82656	-4.94004	-5.13059
H	-7.70551	-2.98511	-3.87261
H	-7.62365	-3.91067	-2.37276
H	-4.89462	1.23030	-2.83885
H	-5.50579	0.63694	-1.27536
H	-3.97536	0.00696	-1.92266
H	-7.85396	-0.98096	-3.61166
H	-7.66235	0.05478	-2.17949
H	-7.17788	0.65644	-3.78216
H	-2.34025	1.47554	-2.89833

H	-0.96501	0.38168	-2.59191
H	-0.99329	1.38528	-4.05407
H	-0.73106	-0.73518	-5.56848
H	-0.61000	-1.68816	-4.06623
H	-1.81529	-2.11717	-5.30730
H	-2.87350	-0.95848	-3.38531
H	-7.89201	0.97242	-7.52623
H	-8.99370	-0.14356	-6.69341
H	-8.05599	1.05040	-5.75837
H	-6.04825	-2.34115	-7.60657
H	-7.81662	-2.16149	-7.77146
H	-6.72480	-1.04947	-8.62801
H	-7.09775	-1.28553	-5.59409
H	-1.90788	1.43295	-6.10581
H	-3.42437	2.08150	-7.95636
H	-5.68219	1.10063	-8.11247
H	-4.87705	-2.07582	-1.43500
C	-1.75949	-2.09616	-9.15543
C	-1.77696	-4.56782	-9.30701
H	-1.41817	-1.12844	-9.53080
C	-2.64553	-2.14816	-8.12635
C	-2.66056	-4.61043	-8.24365
H	-3.01276	-1.23060	-7.66869
H	-3.03495	-5.57414	-7.89979
C	-3.11260	-3.41754	-7.59352
N	-2.89393	2.20988	-14.08701
N	-6.30875	-10.65493	-9.08303
C	-3.13001	-3.89007	-12.79069
C	-3.05744	-2.53369	-12.80752
C	-3.84822	-1.72841	-11.89615
C	-3.80141	-0.36769	-12.03413
C	-3.77259	0.84115	-12.36066
C	-2.96297	5.48123	-9.80897
C	-1.98898	3.22644	-10.36094
C	-2.90918	4.35294	-10.84818
C	-8.16078	1.95010	-12.23231
C	-4.33846	3.90498	-11.25029
C	-5.00151	2.99847	-10.20264
C	-6.47924	2.74606	-10.50359
C	-6.68267	2.15092	-11.89984
C	-5.99450	3.03658	-12.94196
C	-1.47301	4.24423	-14.36693
C	-3.13549	3.64983	-16.11384
C	1.14551	1.91483	-13.60486
C	-0.05535	0.44614	-11.93689
C	-0.21824	1.37636	-13.14988
C	-4.70647	0.67491	-17.43277
C	-5.37775	-0.53242	-15.30850
C	-4.46528	0.54438	-15.92189
C	-0.97330	0.66954	-14.26392
C	-0.41301	-0.48734	-14.82185
C	-1.10823	-1.24634	-15.75887
C	-2.39653	-0.87076	-16.13078
C	-3.00519	0.26940	-15.58991
C	-2.26346	1.06915	-14.68464
C	-4.48427	3.31483	-12.68975
C	-3.96857	4.29159	-13.78786
C	-2.84635	3.59739	-14.60777
C	-3.69104	2.04667	-12.96988
C	-4.67649	-2.41157	-10.95082
C	-4.75536	-3.79052	-10.92285
C	-4.93723	-8.01916	-10.76828
C	-5.09487	-9.24271	-10.55297
C	-1.46878	-12.72538	-9.40880
C	-1.99078	-10.26599	-9.46612
C	-2.58144	-11.68025	-9.56196
C	-5.96826	-11.64699	-14.45497
C	-3.42367	-11.97320	-10.84369
C	-2.91717	-11.20751	-12.07446
C	-3.68078	-11.54359	-13.35411
C	-5.18507	-11.31159	-13.18607
C	-5.68276	-12.13074	-11.99359
C	-4.96320	-11.83262	-10.64948
C	-6.31258	-12.29464	-7.20631
C	-7.97378	-12.52504	-9.04407
C	-5.05499	-9.50738	-5.39403
C	-4.04571	-8.02283	-7.17234
C	-5.03180	-9.16992	-6.89177
C	-10.14582	-10.04919	-10.38986
C	-8.47914	-8.59060	-11.61482
C	-8.67918	-9.60260	-10.47255
C	-6.40982	-8.82804	-7.44114
C	-7.08372	-7.71107	-6.92839
C	-8.27935	-7.26902	-7.48973
C	-8.80048	-7.91870	-8.60605
C	-8.16000	-9.03521	-9.15883
C	-6.98919	-9.52447	-8.52807
C	-5.48680	-12.80944	-9.56136
C	-6.54560	-12.06975	-8.70540
C	-5.40949	-10.48284	-10.11802
H	-0.86481	-12.54001	-8.50555
H	-5.91162	-13.71484	-10.01725
H	-2.52405	-4.47615	-13.48191
H	-2.40547	-2.02117	-13.51378
H	-3.44886	5.14826	-8.87682
H	-1.94868	5.82196	-9.54454
H	-3.52452	6.35184	-10.18568
H	-2.02019	2.34815	-11.01599
H	-0.94653	3.58009	-10.30708
H	-2.26526	2.89086	-9.35003
H	-2.44407	4.78364	-11.74839
H	-8.70256	2.91204	-12.23702
H	-8.28798	1.48767	-13.22485
H	-8.64854	1.29460	-11.49245
H	-4.94198	4.83182	-11.29126
H	-4.90214	3.45807	-9.20654
H	-4.46959	2.03564	-10.16163
H	-7.03730	3.69926	-10.43285
H	-6.91586	2.07621	-9.74228
H	-6.19239	1.16471	-11.91941
H	-6.10887	2.59325	-13.94529
H	-6.51066	4.01505	-12.96970
H	-0.69184	3.72059	-14.93566
H	-1.49027	5.29301	-14.70282
H	-1.19423	4.22839	-13.30526
H	-4.14819	3.29668	-16.34596
H	-3.04921	4.68840	-16.46878
H	-2.41538	3.03701	-16.67666
H	1.82857	1.09542	-13.87900
H	1.05617	2.57610	-14.47969
H	1.62561	2.48507	-12.79285
H	0.61737	-0.39469	-12.17148
H	0.38034	0.99243	-11.08476
H	-1.02378	0.03031	-11.62428
H	-0.82861	2.22608	-12.82739
H	-4.52015	-0.27804	-17.95352
H	-5.75307	0.95755	-17.63221
H	-4.05305	1.43517	-17.88689
H	-5.22502	-0.61363	-14.22408
H	-6.43691	-0.28803	-15.49394
H	-5.17943	-1.52306	-15.74862
H	-4.73745	1.49805	-15.45326
H	0.57016	-0.81844	-14.48621
H	-0.65960	-2.15364	-16.16833
H	-2.95808	-1.49790	-16.82664
H	-3.60772	5.23344	-13.35380

H	-4.79784	4.55788	-14.45819
H	-5.25338	-1.82596	-10.23370
H	-1.87945	-13.74616	-9.33938
H	-0.78411	-12.70097	-10.27421
H	-1.53657	-10.11850	-8.47291
H	-1.19549	-10.10345	-10.21004
H	-2.75021	-9.48672	-9.60606
H	-3.25089	-11.79266	-8.69296
H	-5.61618	-11.04841	-15.31073
H	-5.85273	-12.71199	-14.72208
H	-7.04521	-11.44951	-14.32628
H	-3.28766	-13.04734	-11.07135
H	-2.99214	-10.12536	-11.89325
H	-1.84828	-11.43578	-12.21196
H	-3.51163	-12.60306	-13.62658
H	-3.29254	-10.93950	-14.19217
H	-5.34170	-10.24385	-12.95445
H	-6.76726	-11.98592	-11.86169
H	-5.53733	-13.20359	-12.22097
H	-7.00948	-11.69207	-6.60457
H	-6.47821	-13.35483	-6.95881
H	-5.28643	-12.03306	-6.91559
H	-8.17848	-12.46705	-10.12098
H	-8.11291	-13.57084	-8.72888
H	-8.71538	-11.90958	-8.51479
H	-5.77686	-10.30388	-5.16139
H	-4.05847	-9.83837	-5.05896
H	-5.32470	-8.62545	-4.79290
H	-4.32705	-7.11050	-6.62186
H	-3.02709	-8.30071	-6.85731
H	-4.01960	-7.77589	-8.24231
H	-4.66805	-10.04955	-7.43822
H	-10.81756	-9.19250	-10.21855
H	-10.45687	-10.52555	-11.33410
H	-10.31084	-10.76950	-9.57477
H	-7.43049	-8.26994	-11.68228
H	-8.76754	-9.04117	-12.57915
H	-9.09938	-7.69187	-11.46523
H	-8.06995	-10.47961	-10.71672
H	-6.64304	-7.15588	-6.10029
H	-8.78277	-6.38989	-7.08257
H	-9.70511	-7.53086	-9.07985
H	-4.66271	-13.13822	-8.91512
C	-5.59415	-4.49530	-9.98430
C	-3.97674	-4.59072	-11.85934
H	-6.18268	-3.90919	-9.27591
C	-5.65282	-5.85276	-9.95788
C	-4.04161	-5.96991	-11.82316
H	-6.28732	-6.36879	-9.23697
H	-3.42971	-6.55316	-12.51478
C	-4.86264	-6.65731	-10.87540

2^{dimer}, triplet

N	2.81583	-6.41336	-14.67543
N	-5.22505	-1.19910	-4.18874
C	-1.31017	-5.74573	-9.97802
C	-0.41777	-5.68354	-11.01852
C	0.11631	-4.42197	-11.46216
C	1.05128	-4.43290	-12.52049
C	1.91297	-4.61307	-13.39286
C	6.93178	-3.94030	-12.86888
C	4.79070	-3.79888	-11.55826
C	5.41379	-4.15340	-12.91512
C	2.16191	-1.46794	-17.10671
C	4.79549	-3.41548	-14.14790
C	4.06178	-2.11723	-13.77444
C	3.55722	-1.35372	-14.99574
C	2.64306	-2.21954	-15.86566
C	3.37647	-3.50269	-16.25064
C	4.60684	-8.04128	-15.31173
C	3.22946	-6.97837	-17.08018
C	4.25664	-9.04732	-11.89236
C	3.14020	-7.03168	-10.84366
C	3.45328	-7.76659	-12.15844
C	-0.38303	-7.34784	-17.07122
C	-0.96748	-5.56205	-15.37968
C	0.11388	-6.54417	-15.86126
C	2.17418	-8.02632	-12.94153
C	1.22897	-8.91970	-12.42051
C	-0.01329	-9.08737	-13.02706
C	-0.34148	-8.33674	-14.15305
C	0.57214	-7.43436	-14.71449
C	1.85655	-7.32163	-14.12853
C	3.92672	-4.33607	-15.06213
C	4.75978	-5.51967	-15.62716
C	3.84448	-6.76518	-15.68529
C	2.82106	-5.06831	-14.31918
C	-0.31457	-3.25797	-10.82140
C	-1.24519	-3.30416	-9.75699
C	-3.96647	-3.39857	-6.47574
C	-4.57222	-3.13758	-5.42511
C	-2.96154	-4.29305	-0.75836
C	-2.34215	-4.50205	-3.18538
C	-3.37178	-3.94259	-2.19401
C	-8.73111	-5.54781	-4.20077
C	-4.85166	-4.37123	-2.45560
C	-4.97374	-5.69668	-3.22275
C	-6.42346	-6.15625	-3.34433
C	-7.27126	-5.11082	-4.07084
C	-7.17434	-3.78284	-3.32080
C	-5.74012	-3.24146	-3.06736
C	-5.11265	0.39843	-2.26068
C	-7.31790	-0.25035	-3.18797
C	-1.73593	0.84019	-3.39284
C	-1.39002	-1.25357	-4.77354
C	-2.42632	-0.31827	-4.12693
C	-8.08143	0.36794	-6.68281
C	-6.93441	-1.63071	-7.71923
C	-6.95995	-0.66825	-6.52053
C	-3.42962	0.15789	-5.16759
C	-2.99453	1.03020	-6.17387
C	-3.82806	1.37785	-7.23335
C	-5.10784	0.83639	-7.31503
C	-5.59474	-0.02528	-6.32355
C	-4.75872	-0.32921	-5.22210
C	-5.83376	-2.03408	-2.09363
C	-5.88053	-0.74245	-2.93771
C	-5.15144	-2.58561	-4.30664
H	-1.92414	-3.98441	-0.54869
H	-6.70484	-2.11659	-1.42855
H	-1.71111	-6.71211	-9.66198
H	-0.09962	-6.59277	-11.53008
H	7.17299	-2.86808	-12.76413
H	7.38737	-4.46799	-12.01487
H	7.42054	-4.30036	-13.78934
H	3.70232	-3.94001	-11.55399
H	5.21433	-4.44652	-10.77419
H	5.00253	-2.75699	-11.27043
H	5.23949	-5.23291	-13.05282
H	3.01730	-1.18613	-17.74479
H	1.48070	-2.08480	-17.71516
H	1.62487	-0.54663	-16.83453
H	5.63791	-3.11256	-14.79751
H	4.73843	-1.47563	-13.18696
H	3.20746	-2.34961	-13.12149

H	4.41367	-1.01343	-15.60823
H	3.02088	-0.44362	-14.67977
H	1.76628	-2.50630	-15.25835
H	2.71862	-4.13495	-16.86626
H	4.23437	-3.22978	-16.89336
H	3.92487	-8.90157	-15.23232
H	5.34852	-8.26749	-16.09323
H	5.14263	-7.93119	-14.36075
H	2.68563	-6.09362	-17.43428
H	4.02519	-7.20158	-17.80788
H	2.53368	-7.82958	-17.06891
H	3.71202	-9.73226	-11.22261
H	4.47450	-9.59422	-12.82196
H	5.21478	-8.80534	-11.40432
H	2.53293	-7.65792	-10.17046
H	4.07301	-6.77403	-10.31664
H	2.58508	-6.10333	-11.03072
H	4.07466	-7.09137	-12.75959
H	-1.28779	-7.92633	-16.82413
H	-0.64244	-6.67262	-17.90291
H	0.37623	-8.05801	-17.43179
H	-0.61257	-4.97727	-14.52038
H	-1.24356	-4.86118	-16.18502
H	-1.88295	-6.09623	-15.07750
H	0.97418	-5.94367	-16.17846
H	1.45705	-9.47134	-11.50562
H	-0.74152	-9.77652	-12.60178
H	-1.33671	-8.43921	-14.59196
H	5.60597	-5.73526	-14.96149
H	5.17949	-5.28131	-16.61466
H	0.04860	-2.28539	-11.15175
H	-3.61804	-3.80460	-0.01941
H	-3.02315	-5.38206	-0.58805
H	-1.35436	-4.05570	-2.98787
H	-2.23267	-5.59364	-3.08662
H	-2.60464	-4.27928	-4.22703
H	-3.32193	-2.84553	-2.28953
H	-8.81668	-6.52716	-4.69537
H	-9.19900	-5.63783	-3.20537
H	-9.32138	-4.81934	-4.78088
H	-5.30963	-4.55666	-1.46594
H	-4.54497	-5.58870	-4.23154
H	-4.37585	-6.46778	-2.70981
H	-6.84995	-6.32555	-2.33741
H	-6.48009	-7.12491	-3.86595
H	-6.84927	-4.96964	-5.08341
H	-7.76526	-3.01741	-3.84596
H	-7.65490	-3.92199	-2.33458
H	-5.04245	1.27653	-2.92022
H	-5.64516	0.70266	-1.34638
H	-4.09803	0.09545	-1.97449
H	-7.94569	-1.01835	-3.65602
H	-7.78564	0.03446	-2.23274
H	-7.31378	0.63526	-3.83931
H	-2.46365	1.53431	-2.94597
H	-1.09058	0.45350	-2.58742
H	-1.09395	1.42355	-4.07254
H	-0.79682	-0.72514	-5.53735
H	-0.69447	-1.64138	-4.01150
H	-1.87630	-2.10928	-5.25966
H	-2.97520	-0.91481	-3.38738
H	-7.94861	0.96144	-7.60199
H	-9.06085	-0.13282	-6.75401
H	-8.11728	1.07010	-5.83631
H	-6.12414	-2.36423	-7.61453
H	-7.88987	-2.17506	-7.80006
H	-6.78271	-1.08481	-8.66464
H	-7.17580	-1.27049	-5.63037

H	-1.97355	1.41699	-6.14497
H	-3.46920	2.03860	-8.02159
H	-5.73394	1.07099	-8.17673
H	-4.94105	-2.00131	-1.45474
C	-1.71820	-2.12096	-9.11895
C	-1.75269	-4.56750	-9.30483
H	-1.35979	-1.15206	-9.47371
C	-2.61683	-2.17947	-8.08583
C	-2.67067	-4.61146	-8.22774
H	-2.97252	-1.26389	-7.61590
H	-3.05286	-5.57689	-7.89936
C	-3.10132	-3.44156	-7.59472
N	-2.82858	2.18704	-14.05332
N	-6.25654	-10.67686	-9.00937
C	-3.13061	-3.89105	-12.73932
C	-3.06103	-2.52802	-12.74681
C	-3.85265	-1.73716	-11.83715
C	-3.79516	-0.35589	-11.97143
C	-3.76703	0.83437	-12.33685
C	-3.04727	5.53184	-9.82718
C	-2.03289	3.28810	-10.34999
C	-2.95640	4.40084	-10.86079
C	-8.15784	1.95141	-12.33990
C	-4.36909	3.93130	-11.29341
C	-5.05051	3.03419	-10.24885
C	-6.51953	2.77002	-10.58081
C	-6.68815	2.15613	-11.97380
C	-5.97374	3.02573	-13.01225
C	-1.41069	4.22816	-14.30497
C	-2.99559	3.59507	-16.10937
C	1.20223	1.91173	-13.44856
C	-0.06452	0.49691	-11.78107
C	-0.17843	1.38941	-13.02695
C	-4.50672	0.59323	-17.45030
C	-5.26476	-0.56854	-15.33000
C	-4.32674	0.49448	-15.92848
C	-0.89700	0.65343	-14.14612
C	-0.31583	-0.51211	-14.66293
C	-0.97794	-1.29056	-15.60800
C	-2.25252	-0.92361	-16.03178
C	-2.88130	0.22535	-15.53360
C	-2.17602	1.03954	-14.61222
C	-4.47104	3.31132	-12.72477
C	-3.92792	4.26605	-13.82961
C	-2.76845	3.56687	-14.59221
C	-3.66274	2.04379	-12.95593
C	-4.67867	-2.41299	-10.90927
C	-4.75602	-3.80848	-10.88755
C	-4.91954	-8.04837	-10.74322
C	-5.07261	-9.26195	-10.51112
C	-1.39042	-12.71167	-9.44540
C	-1.92708	-10.25594	-9.52937
C	-2.51287	-11.67476	-9.58156
C	-6.05804	-11.72741	-14.36138
C	-3.39417	-11.99422	-10.83026
C	-2.93448	-11.24066	-12.08702
C	-3.73568	-11.59777	-13.33768
C	-5.23524	-11.37493	-13.12252
C	-5.68599	-12.18589	-11.90608
C	-4.92806	-11.86199	-10.58907
C	-6.22750	-12.29852	-7.11565
C	-7.89949	-12.56654	-8.93667
C	-4.98003	-9.48135	-5.33682
C	-3.98765	-8.02724	-7.14954
C	-4.97079	-9.16928	-6.84011
C	-10.11702	-10.12610	-10.25825
C	-8.49098	-8.66439	-11.53323
C	-8.65783	-9.66284	-10.37400

C	-6.35437	-8.83759	-7.38148
C	-7.02901	-7.71949	-6.87221
C	-8.23253	-7.28899	-7.42582
C	-8.76255	-7.95375	-8.52911
C	-8.12228	-9.07291	-9.07694
C	-6.93931	-9.54676	-8.45721
C	-5.41150	-12.82585	-9.47124
C	-6.47380	-12.09071	-8.61514
C	-5.36938	-10.51124	-10.05970
H	-0.75781	-12.50632	-8.56638
H	-5.82054	-13.75245	-9.89827
H	-2.52486	-4.46587	-13.43969
H	-2.40930	-2.01172	-13.44980
H	-3.54411	5.19514	-8.90224
H	-2.04324	5.88954	-9.54642
H	-3.61546	6.39195	-10.21776
H	-2.03708	2.40911	-11.00464
H	-0.99650	3.65494	-10.27205
H	-2.32861	2.94927	-9.34577
H	-2.47775	4.83553	-11.75192
H	-8.69751	2.91392	-12.37388
H	-8.26092	1.47332	-13.32783
H	-8.66556	1.30892	-11.60202
H	-4.98013	4.85133	-11.36565
H	-4.97614	3.50424	-9.25540
H	-4.51481	2.07482	-10.18516
H	-7.08345	3.72124	-10.53491
H	-6.97048	2.10722	-9.82153
H	-6.19857	1.16940	-11.96677
H	-6.06010	2.56377	-14.01004
H	-6.49201	4.00176	-13.07163
H	-0.60243	3.70398	-14.83414
H	-1.42267	5.27218	-14.65557
H	-1.17557	4.22963	-13.23276
H	-3.99503	3.23010	-16.37785
H	-2.90287	4.62904	-16.47592
H	-2.24810	2.98035	-16.63313
H	1.89943	1.08515	-13.65928
H	1.14645	2.53514	-14.35371
H	1.64713	2.51865	-12.64309
H	0.60050	-0.36096	-11.97045
H	0.35607	1.06367	-10.93466
H	-1.04890	0.10764	-11.48400
H	-0.79623	2.24993	-12.75184
H	-4.30232	-0.37096	-17.94292
H	-5.54356	0.87375	-17.69781
H	-3.83318	1.34223	-17.89345
H	-5.16147	-0.62012	-14.23800
H	-6.31477	-0.33206	-15.56974
H	-5.04514	-1.56980	-15.73443
H	-4.61572	1.45800	-15.49086
H	0.65817	-0.83304	-14.29191
H	-0.51453	-2.20487	-15.98367
H	-2.78767	-1.56329	-16.73702
H	-3.59529	5.22437	-13.40903
H	-4.73631	4.50251	-14.53577
H	-5.26634	-1.83449	-10.19563
H	-1.79361	-13.73261	-9.34270
H	-0.73492	-12.70126	-10.33342
H	-1.43762	-10.09110	-8.55573
H	-1.16089	-10.09894	-10.30475
H	-2.69611	-9.48307	-9.65259
H	-3.15368	-11.77421	-8.68967
H	-5.73838	-11.13547	-15.23433
H	-5.94527	-12.79437	-14.62167
H	-7.13127	-11.53469	-14.19869
H	-3.25539	-13.07007	-11.04763
H	-3.01538	-10.15720	-11.91677
H	-1.86788	-11.45922	-12.25587
H	-3.56723	-12.65893	-13.60359
H	-3.37950	-11.00011	-14.19435
H	-5.39187	-10.30593	-12.89617
H	-6.76702	-12.05004	-11.74215
H	-5.53676	-13.25973	-12.12653
H	-6.92584	-11.69704	-6.51440
H	-6.38140	-13.35778	-6.85699
H	-5.20185	-12.02513	-6.83439
H	-8.11612	-12.52127	-10.01170
H	-8.02283	-13.61089	-8.61045
H	-8.64335	-11.95526	-8.40560
H	-5.69838	-10.27539	-5.08532
H	-3.98012	-9.80500	-5.00481
H	-5.24661	-8.59021	-4.74791
H	-4.26608	-7.10504	-6.61429
H	-2.96645	-8.29855	-6.83669
H	-3.96980	-7.80219	-8.22454
H	-4.61165	-10.05752	-7.37536
H	-10.79621	-9.27554	-10.08571
H	-10.43941	-10.61928	-11.18997
H	-10.25752	-10.83722	-9.43055
H	-7.44679	-8.33563	-11.62594
H	-8.79413	-9.13056	-12.48556
H	-9.11677	-7.76957	-11.38317
H	-8.04236	-10.53549	-10.61753
H	-6.58398	-7.15588	-6.05208
H	-8.73684	-6.40906	-7.02164
H	-9.67537	-7.57682	-8.99600
H	-4.56945	-13.11736	-8.83010
C	-5.59296	-4.51349	-9.95972
C	-3.97545	-4.59117	-11.81983
H	-6.18457	-3.93587	-9.24712
C	-5.64621	-5.87711	-9.93959
C	-4.03881	-5.98711	-11.78973
H	-6.27938	-6.39784	-9.22107
H	-3.42012	-6.56279	-12.48159
C	-4.85252	-6.66706	-10.85498

2^{dimer}, quintet

N	2.78816	-6.47691	-14.63224
N	-5.28046	-1.22229	-4.18085
C	-1.29129	-5.76294	-9.93033
C	-0.41395	-5.70349	-10.98343
C	0.09213	-4.44093	-11.45462
C	1.01462	-4.45690	-12.52392
C	1.87060	-4.65001	-13.39915
C	6.89763	-3.86972	-12.93114
C	4.75696	-3.77105	-11.61598
C	5.38738	-4.13452	-12.96688
C	2.07238	-1.59902	-17.19984
C	4.74230	-3.44324	-14.21199
C	4.00270	-2.14073	-13.86690
C	3.48324	-1.41545	-15.10493
C	2.56390	-2.31063	-15.93918
C	3.29889	-3.60267	-16.29290
C	4.59639	-8.09881	-15.23044
C	3.19220	-7.11188	-17.02223
C	4.29587	-8.98273	-11.76854
C	3.15442	-6.95525	-10.77427
C	3.46854	-7.72473	-12.06882
C	-0.40995	-7.56607	-16.96611
C	-1.01281	-5.72519	-15.34222
C	0.08180	-6.70706	-15.79280
C	2.18801	-8.03336	-12.83171
C	1.26222	-8.92151	-12.26853

C	0.01920	-9.13570	-12.85830
C	-0.32929	-8.43796	-14.01183
C	0.56418	-7.54236	-14.61520
C	1.85013	-7.38213	-14.04356
C	3.87099	-4.39804	-15.08837
C	4.71183	-5.58721	-15.63016
C	3.81357	-6.84632	-15.63937
C	2.78058	-5.12186	-14.31523
C	-0.34922	-3.27285	-10.82801
C	-1.26236	-3.31618	-9.74834
C	-3.94224	-3.41239	-6.43142
C	-4.56091	-3.15540	-5.38762
C	-2.95266	-4.17695	-0.68320
C	-2.28989	-4.41479	-3.09633
C	-3.35296	-3.87113	-2.13176
C	-8.63739	-5.69619	-4.14813
C	-4.81317	-4.35671	-2.40521
C	-4.87765	-5.69071	-3.16507
C	-6.30668	-6.20925	-3.29554
C	-7.19558	-5.20064	-4.02553
C	-7.14961	-3.86401	-3.28548
C	-5.73755	-3.26624	-3.03490
C	-5.23625	0.40358	-2.27435
C	-7.41213	-0.33063	-3.20862
C	-1.86348	0.92821	-3.38411
C	-1.43114	-1.17178	-4.73053
C	-2.50588	-0.26322	-4.10911
C	-8.15130	0.24527	-6.72604
C	-6.95950	-1.76169	-7.69787
C	-7.01245	-0.76545	-6.52824
C	-3.51180	0.16529	-5.16760
C	-3.08902	1.02814	-6.18718
C	-3.92049	1.33485	-7.26076
C	-5.18724	0.76301	-7.34125
C	-5.66188	-0.09088	-6.33689
C	-4.82847	-0.35436	-5.22294
C	-5.87980	-2.04912	-2.07892
C	-5.96106	-0.77077	-2.94120
C	-5.16419	-2.60728	-4.27987
H	-1.93015	-3.82739	-0.46476
H	-6.75429	-2.15103	-1.42106
H	-1.67135	-6.73044	-9.59294
H	-0.08655	-6.61561	-11.48322
H	7.10346	-2.78762	-12.85703
H	7.37228	-4.35777	-12.06408
H	7.39586	-4.23916	-13.84272
H	3.67352	-3.94496	-11.60570
H	5.20220	-4.38847	-10.81964
H	4.93748	-2.71729	-11.35103
H	5.24969	-5.22199	-13.08141
H	2.92297	-1.33690	-17.85254
H	1.38813	-2.23565	-17.78415
H	1.53706	-0.66962	-16.95346
H	5.57255	-3.15233	-14.88248
H	4.68037	-1.47878	-13.30349
H	3.15571	-2.36077	-13.19998
H	4.33285	-1.09220	-15.73590
H	2.94859	-0.49666	-14.81168
H	1.69258	-2.58024	-15.31566
H	2.63652	-4.25682	-16.88041
H	4.14650	-3.34433	-16.95500
H	3.92734	-8.96559	-15.11855
H	5.33653	-8.33976	-16.00896
H	5.13605	-7.95005	-14.28698
H	2.63477	-6.24546	-17.39999
H	3.98657	-7.34700	-17.74767
H	2.50824	-7.97155	-16.98015
H	3.76831	-9.65361	-11.07149
H	4.51444	-9.55673	-12.68134
H	5.25415	-8.70910	-11.29775
H	2.56234	-7.57000	-10.07740
H	4.08743	-6.66842	-10.26312
H	2.58376	-6.04135	-10.98369
H	4.07286	-7.05851	-12.69703
H	-1.30159	-8.15103	-16.68844
H	-0.68901	-6.92861	-17.82094
H	0.35922	-8.27587	-17.30585
H	-0.66341	-5.10819	-14.50356
H	-1.29874	-5.05435	-16.16938
H	-1.92106	-6.26037	-15.02054
H	0.92884	-6.10412	-16.13946
H	1.50614	-9.43148	-11.33381
H	-0.69470	-9.81754	-12.39871
H	-1.32533	-8.57590	-14.43909
H	5.57024	-5.77149	-14.97082
H	5.11465	-5.37247	-16.63001
H	-0.00297	-2.30359	-11.18545
H	-3.63725	-3.69683	0.03541
H	-2.97900	-5.26367	-0.49063
H	-1.32176	-3.92876	-2.89563
H	-2.14273	-5.49928	-2.97171
H	-2.54514	-4.22460	-4.14624
H	-3.33934	-2.77531	-2.24981
H	-8.68675	-6.67584	-4.64707
H	-9.09427	-5.80801	-3.14982
H	-9.26069	-4.98992	-4.72120
H	-5.27414	-4.55238	-1.41893
H	-4.44481	-5.57141	-4.17074
H	-4.25428	-6.43459	-2.64247
H	-6.73099	-6.39705	-2.29094
H	-6.32014	-7.17917	-3.81852
H	-6.78197	-5.04869	-5.03985
H	-7.76877	-3.12575	-3.81712
H	-7.62494	-4.01287	-2.29815
H	-5.18877	1.27411	-2.94589
H	-5.78588	0.70358	-1.36881
H	-4.21493	0.13728	-1.97586
H	-8.01028	-1.12674	-3.66868
H	-7.89589	-0.04786	-2.26079
H	-7.43374	0.54482	-3.87327
H	-2.61946	1.60431	-2.95711
H	-1.21601	0.57537	-2.56493
H	-1.23279	1.52239	-4.06499
H	-0.84424	-0.63291	-5.49195
H	-0.73430	-1.52868	-3.95476
H	-1.88316	-2.04791	-5.21339
H	-3.04354	-0.86721	-3.36737
H	-8.02619	0.81278	-7.66260
H	-9.12176	-0.27426	-6.78407
H	-8.20149	0.97250	-5.90149
H	-6.14714	-2.48674	-7.55540
H	-7.91012	-2.31435	-7.77962
H	-6.79338	-1.24346	-8.65626
H	-7.22453	-1.34705	-5.62385
H	-2.07797	1.44014	-6.15682
H	-3.57021	1.98795	-8.05901
H	-5.81331	0.96844	-8.21090
H	-4.99531	-1.97835	-1.43177
C	-1.74360	-2.13199	-9.11757
C	-1.74294	-4.58103	-9.27006
H	-1.41281	-1.16077	-9.49281
C	-2.62264	-2.19059	-8.06747
C	-2.64592	-4.62415	-8.18066
H	-2.98426	-1.27429	-7.60344
H	-3.01583	-5.59028	-7.83999
C	-3.08470	-3.45302	-7.55584

N	-2.82012	2.25126	-14.05052
N	-6.23085	-10.63229	-9.06832
C	-3.09341	-3.84132	-12.77885
C	-3.05842	-2.47131	-12.78901
C	-3.84167	-1.70663	-11.86083
C	-3.78993	-0.30129	-11.97687
C	-3.77235	0.87869	-12.35047
C	-3.04457	5.54847	-9.79019
C	-2.04975	3.29872	-10.32424
C	-2.95795	4.42561	-10.83303
C	-8.16146	2.01752	-12.37293
C	-4.37257	3.97509	-11.27981
C	-5.06501	3.06868	-10.25034
C	-6.53268	2.81244	-10.59378
C	-6.69387	2.21660	-11.99561
C	-5.97190	3.09907	-13.01808
C	-1.39010	4.28674	-14.26582
C	-2.96408	3.68598	-16.09080
C	1.20910	1.89883	-13.48604
C	-0.06299	0.47516	-11.83007
C	-0.17568	1.39205	-13.05857
C	-4.57276	0.73158	-17.43989
C	-5.31323	-0.44821	-15.32278
C	-4.36895	0.60836	-15.92299
C	-0.91548	0.68711	-14.18432
C	-0.35729	-0.47759	-14.72875
C	-1.04215	-1.22929	-15.67950
C	-2.31671	-0.83727	-16.08072
C	-2.92205	0.31157	-15.55478
C	-2.19217	1.10213	-14.63179
C	-4.46900	3.37116	-12.71873
C	-3.91175	4.33377	-13.81043
C	-2.74930	3.63679	-14.57232
C	-3.66125	2.10710	-12.95436
C	-4.63398	-2.38582	-10.93180
C	-4.68439	-3.79818	-10.91123
C	-4.78317	-8.05191	-10.77590
C	-4.97261	-9.25446	-10.55199
C	-1.47603	-12.93179	-9.46370
C	-1.92604	-10.45671	-9.46364
C	-2.55821	-11.85087	-9.58278
C	-5.96231	-11.62845	-14.45757
C	-3.42210	-12.08917	-10.86000
C	-2.88873	-11.33176	-12.08485
C	-3.66893	-11.62535	-13.36485
C	-5.16194	-11.33240	-13.18965
C	-5.68890	-12.13575	-11.99870
C	-4.95261	-11.87059	-10.65643
C	-6.29680	-12.28372	-7.20274
C	-7.98376	-12.42031	-9.02649
C	-4.87907	-9.58141	-5.38181
C	-3.81111	-8.14461	-7.16316
C	-4.85529	-9.23792	-6.87823
C	-10.06109	-9.82776	-10.29593
C	-8.34940	-8.44534	-11.54607
C	-8.57585	-9.45411	-10.40616
C	-6.21967	-8.81949	-7.40791
C	-6.82949	-7.67692	-6.87249
C	-8.01074	-7.17092	-7.41006
C	-8.58023	-7.78307	-8.52394
C	-8.00371	-8.92347	-9.09884
C	-6.84680	-9.47518	-8.49453
C	-5.51956	-12.82395	-9.56810
C	-6.53232	-12.03668	-8.69795
C	-5.32605	-10.50631	-10.11372
H	-0.85685	-12.78333	-8.56385
H	-5.99486	-13.70341	-10.02497
H	-2.48341	-4.40312	-13.48589
H	-2.43241	-1.94055	-13.50424
H	-3.55435	5.20947	-8.87310
H	-2.03911	5.89181	-9.49671
H	-3.59890	6.41871	-10.17823
H	-2.05540	2.42562	-10.98734
H	-1.01075	3.65535	-10.23363
H	-2.35796	2.95566	-9.32484
H	-2.46802	4.86258	-11.71676
H	-8.70007	2.98089	-12.39806
H	-8.25891	1.55222	-13.36755
H	-8.67439	1.36619	-11.64639
H	-4.97625	4.90072	-11.34258
H	-4.99469	3.52601	-9.25061
H	-4.53381	2.10633	-10.19577
H	-7.09524	3.76400	-10.53937
H	-6.98843	2.14107	-9.84494
H	-6.20506	1.22927	-11.99824
H	-6.05556	2.65177	-14.02276
H	-6.48705	4.07759	-13.06523
H	-0.58158	3.76899	-14.80091
H	-1.39521	5.33756	-14.59594
H	-1.16098	4.26563	-13.19256
H	-3.96361	3.33135	-16.37227
H	-2.86199	4.72410	-16.44288
H	-2.21649	3.07346	-16.61708
H	1.88593	1.06304	-13.72517
H	1.15331	2.54376	-14.37600
H	1.67655	2.47781	-12.67284
H	0.59065	-0.38652	-12.04027
H	0.37014	1.02115	-10.97634
H	-1.04932	0.09143	-11.53266
H	-0.77825	2.25647	-12.76192
H	-4.39463	-0.22884	-17.94973
H	-5.60822	1.03476	-17.66558
H	-3.89306	1.47466	-17.88374
H	-5.19017	-0.51699	-14.23366
H	-6.36363	-0.19118	-15.53858
H	-5.11750	-1.44647	-15.74658
H	-4.63552	1.56968	-15.46717
H	0.61601	-0.82058	-14.37572
H	-0.59605	-2.14210	-16.07895
H	-2.86984	-1.45667	-16.79015
H	-3.57765	5.28639	-13.37798
H	-4.71354	4.58110	-14.52047
H	-5.22254	-1.82221	-10.20698
H	-1.91730	-13.94062	-9.40939
H	-0.80030	-12.91134	-10.33617
H	-1.45905	-10.34223	-8.47195
H	-1.13289	-10.30169	-10.21182
H	-2.66351	-9.65284	-9.58146
H	-3.22196	-11.96198	-8.70941
H	-5.58767	-11.04222	-15.31240
H	-5.89315	-12.69639	-14.72884
H	-7.02948	-11.38593	-14.32472
H	-3.33860	-13.16595	-11.10029
H	-2.92779	-10.24962	-11.89203
H	-1.82796	-11.59295	-12.22925
H	-3.54443	-12.68866	-13.64601
H	-3.25829	-11.03111	-14.19938
H	-5.27264	-10.25983	-12.95326
H	-6.76574	-11.94494	-11.86112
H	-5.59026	-13.21265	-12.23314
H	-6.95806	-11.65305	-6.58957
H	-6.51077	-13.33654	-6.96091
H	-5.25641	-12.07416	-6.92083
H	-8.19548	-12.34234	-10.10085
H	-8.16929	-13.46145	-8.71977
H	-8.69060	-11.77610	-8.48409

H	-5.63385	-10.34586	-5.14636
H	-3.89560	-9.95936	-5.05790
H	-5.10333	-8.69119	-4.77343
H	-4.02515	-7.22997	-6.58679
H	-2.80337	-8.48804	-6.87937
H	-3.79690	-7.88021	-8.22951
H	-4.54590	-10.13196	-7.43430
H	-10.68597	-8.93964	-10.10827
H	-10.41404	-10.28382	-11.23538
H	-10.24549	-10.54271	-9.48026
H	-7.28516	-8.19168	-11.64387
H	-8.69491	-8.86746	-12.50439
H	-8.90687	-7.51095	-11.37031
H	-8.01515	-10.35797	-10.66829
H	-6.34959	-7.15465	-6.04444
H	-8.46446	-6.27327	-6.98583
H	-9.47289	-7.34741	-8.97847
H	-4.70908	-13.19988	-8.93046
C	-5.49149	-4.51306	-9.97704
C	-3.90228	-4.55277	-11.84827
H	-6.08586	-3.94730	-9.25780
C	-5.51576	-5.88205	-9.95840
C	-3.93596	-5.96676	-11.81552
H	-6.13011	-6.41462	-9.23363
H	-3.31366	-6.52708	-12.51608
C	-4.72150	-6.64827	-10.88342

3^{dimer}, singlet

N	1.80547	-6.18334	-16.26764
N	-7.71073	-2.47743	-4.16712
C	-0.98524	-5.40983	-10.66091
C	-0.27417	-5.39663	-11.83013
C	-0.02196	-4.16736	-12.54132
C	0.59595	-4.20871	-13.76532
C	1.13784	-4.37965	-14.88018
C	5.76069	-2.82353	-16.60941
C	4.38591	-3.18970	-14.53618
C	4.46620	-3.42733	-16.05006
C	-0.79757	-1.67442	-18.19885
C	3.23565	-2.92390	-16.86748
C	2.57194	-1.68416	-16.25167
C	1.44074	-1.13659	-17.11931
C	0.36568	-2.20191	-17.35774
C	1.01490	-3.42516	-18.01121
C	3.63347	-7.46934	-17.37005
C	1.44365	-7.21146	-18.52292
C	4.56416	-8.13808	-13.98467
C	3.48078	-6.26116	-12.68279
C	3.48034	-7.05060	-14.00442
C	-1.82335	-7.87807	-17.25353
C	-2.04424	-5.94178	-15.64245
C	-1.06233	-6.85740	-16.39461
C	2.09112	-7.60798	-14.28326
C	1.55424	-8.54818	-13.39353
C	0.23705	-8.98225	-13.51113
C	-0.57957	-8.45417	-14.50837
C	-0.08975	-7.51276	-15.42366
C	1.27389	-7.13563	-15.33819
C	2.20599	-4.04139	-17.22572
C	2.87705	-5.14442	-18.09300
C	2.43537	-6.53197	-17.56599
C	1.69173	-4.82859	-16.03202
C	-0.48797	-2.95946	-11.91976
C	-1.20725	-2.99437	-10.75514
C	-1.53843	-4.21816	-10.08158
C	-5.46177	-4.33530	-5.97181
C	-6.37025	-4.19197	-5.12298
C	-5.93748	-5.33206	-0.16692
C	-4.61098	-5.07331	-2.28636
C	-5.98837	-4.89030	-1.63478
C	-9.92751	-7.34997	-5.35705
C	-7.17800	-5.59013	-2.36847
C	-6.74614	-6.82763	-3.17186
C	-7.92388	-7.56467	-3.80665
C	-8.74355	-6.63460	-4.70580
C	-9.20739	-5.42912	-3.89051
C	-8.07138	-4.62220	-3.20479
C	-8.38029	-0.99568	-2.26738
C	-10.12354	-1.94511	-3.75811
C	-5.01959	0.20030	-2.64115
C	-3.89570	-1.70081	-3.87821
C	-5.23921	-1.04843	-3.50701
C	-10.16767	-1.58763	-7.32922
C	-8.34866	-3.20557	-8.00081
C	-8.89830	-2.31824	-6.86895
C	-6.05539	-0.76114	-4.75993
C	-5.59590	0.20347	-5.66797
C	-6.23682	0.40821	-6.88796
C	-7.32622	-0.38692	-7.23623
C	-7.81751	-1.37033	-6.36727
C	-7.21147	-1.50710	-5.09448
C	-8.70500	-3.51994	-2.31453
C	-8.74300	-2.20757	-3.13290
C	-7.30576	-3.79647	-4.22752
H	-5.10693	-4.84664	0.37120
H	-9.70869	-3.80944	-1.97291
H	-1.17729	-6.37528	-10.19316
H	0.08407	-6.33119	-12.26455
H	5.73900	-1.72183	-16.54845
H	6.64150	-3.16854	-16.04349
H	5.90936	-3.09395	-17.66789
H	3.45969	-3.58590	-14.10265
H	5.23164	-3.68722	-14.03480
H	4.44603	-2.11774	-14.28992
H	4.53347	-4.51921	-16.18897
H	-0.45322	-1.36458	-19.20076
H	-1.57733	-2.44166	-18.33482
H	-1.26625	-0.79604	-17.72585
H	3.62108	-2.60424	-17.85399
H	3.33812	-0.90701	-16.09350
H	2.16902	-1.93297	-15.25834
H	1.84065	-0.80673	-18.09719
H	1.00032	-0.24305	-16.64734
H	-0.02949	-2.51142	-16.37352
H	0.25896	-4.20807	-18.19000
H	1.39223	-3.12323	-19.00606
H	3.31960	-8.41895	-16.91148
H	4.08997	-7.69708	-18.34584
H	4.40180	-7.01243	-16.73219
H	0.60193	-6.55297	-18.77512
H	1.95884	-7.47529	-19.45950
H	1.04480	-8.13675	-18.08301
H	4.40527	-8.84699	-13.15596
H	4.58247	-8.71730	-14.91968
H	5.55930	-7.68572	-13.84272
H	3.27844	-6.92242	-11.82472
H	4.46172	-5.78692	-12.51869
H	2.71463	-5.47391	-12.68729
H	3.72124	-6.34035	-14.80566
H	-2.48431	-8.50801	-16.63655
H	-2.45996	-7.36124	-17.98981
H	-1.14210	-8.54690	-17.80097
H	-1.50872	-5.22456	-15.00482
H	-2.66423	-5.37589	-16.35726
H	-2.72549	-6.52467	-15.00445

H	-0.47970	-6.21494	-17.06407
H	2.17066	-8.92345	-12.57354
H	-0.16618	-9.70506	-12.80341
H	-1.62603	-8.76281	-14.56145
H	3.97009	-5.06264	-18.03688
H	2.60787	-5.02564	-19.15173
H	-0.30440	-2.00477	-12.41553
H	-1.56530	-2.04903	-10.35435
H	-6.87350	-5.08816	0.36207
H	-5.78918	-6.42332	-0.09140
H	-3.86697	-4.45385	-1.75993
H	-4.26598	-6.11773	-2.22621
H	-4.60810	-4.77260	-3.34128
H	-6.18499	-3.80523	-1.64057
H	-9.59539	-8.21298	-5.95583
H	-10.62710	-7.72674	-4.59094
H	-10.49122	-6.67318	-6.02027
H	-7.86165	-5.96047	-1.58169
H	-6.04679	-6.52634	-3.96584
H	-6.19065	-7.51054	-2.50807
H	-8.58605	-7.96784	-3.01665
H	-7.56111	-8.43185	-4.38286
H	-8.07948	-6.25573	-5.50329
H	-9.80095	-4.75444	-4.52642
H	-9.88866	-5.78719	-3.09625
H	-8.29827	-0.08387	-2.87809
H	-9.16841	-0.83132	-1.51638
H	-7.43224	-1.14651	-1.73544
H	-10.47516	-2.79251	-4.36000
H	-10.86260	-1.76339	-2.96238
H	-10.09239	-1.05370	-4.40079
H	-5.96961	0.68740	-2.37514
H	-4.49776	-0.06626	-1.70761
H	-4.39718	0.94563	-3.16209
H	-3.25810	-1.00473	-4.44684
H	-3.34776	-1.99501	-2.96828
H	-4.04490	-2.59796	-4.49398
H	-5.79670	-1.78391	-2.91276
H	-9.96669	-0.94880	-8.20418
H	-10.94271	-2.31329	-7.62492
H	-10.58408	-0.94716	-6.53767
H	-7.42288	-3.71307	-7.69569
H	-9.08793	-3.97403	-8.28081
H	-8.13321	-2.60860	-8.90120
H	-9.16808	-2.98376	-6.04051
H	-4.70590	0.78849	-5.42492
H	-5.86641	1.16195	-7.58476
H	-7.79162	-0.25815	-8.21582
H	-8.09367	-3.36241	-1.41626
C	-2.43447	-4.24864	-8.96930
C	-3.11562	-3.07014	-8.51236
C	-2.78690	-5.46639	-8.29497
H	-2.90523	-2.10936	-8.97965
H	-2.30861	-6.39950	-8.58921
C	-4.08244	-3.10560	-7.54555
C	-3.74174	-5.51875	-7.31449
H	-4.60715	-2.19144	-7.26134
H	-4.00039	-6.47504	-6.85900
C	-4.45791	-4.34195	-6.90644
N	-2.68168	3.62456	-12.36529
N	-4.99257	-11.75105	-9.28173
C	-3.92862	-2.54565	-12.43468
C	-3.87858	-1.18047	-12.37475
C	-4.64377	-0.44629	-11.39802
C	-4.47568	0.91008	-11.30629
C	-4.19381	2.12842	-11.30982
C	-2.73243	5.01264	-7.29498
C	-3.03023	2.63615	-8.05977
C	-3.01709	4.10687	-8.49998
C	-7.97285	4.84602	-11.71067
C	-4.29788	4.59136	-9.25174
C	-5.56040	3.82287	-8.83035
C	-6.83225	4.34926	-9.49252
C	-6.71112	4.33016	-11.01896
C	-5.48137	5.14016	-11.43387
C	-0.74442	5.19902	-12.29302
C	-2.69075	5.70897	-13.75252
C	1.03693	2.22487	-11.38187
C	-0.80759	0.75038	-10.47658
C	-0.47710	1.97317	-11.35073
C	-4.08360	3.50287	-16.21167
C	-5.32546	1.88369	-14.71708
C	-4.15892	2.88332	-14.80870
C	-1.07471	1.80122	-12.73972
C	-0.59936	0.76594	-13.55696
C	-1.22138	0.45699	-14.76416
C	-2.34524	1.17555	-15.16543
C	-2.85054	2.22775	-14.38950
C	-2.17393	2.57038	-13.19271
C	-4.14082	4.67032	-10.80216
C	-3.00817	5.64950	-11.21278
C	-2.26407	5.04804	-12.43172
C	-3.69439	3.37639	-11.45874
C	-5.50968	-1.21207	-10.54578
C	-5.53489	-2.57918	-10.61244
C	-4.72134	-3.32697	-11.52867
C	-4.12568	-8.95352	-11.06170
C	-4.09362	-10.18061	-10.82046
C	-0.10899	-13.37971	-10.01970
C	-0.73452	-10.94553	-10.06286
C	-1.27025	-12.38256	-10.11450
C	-5.01882	-12.53563	-14.73228
C	-2.17519	-12.71533	-11.34348
C	-1.85120	-11.85682	-12.57463
C	-2.67258	-12.23359	-13.80560
C	-4.17426	-12.15907	-13.51511
C	-4.49820	-13.05367	-12.31606
C	-3.70031	-12.73589	-11.02098
C	-4.75809	-13.49320	-7.51016
C	-6.49732	-13.75465	-9.26805
C	-3.37134	-10.75150	-5.67398
C	-2.61316	-9.18008	-7.50547
C	-3.53213	-10.36228	-7.15060
C	-9.00600	-11.31345	-9.98176
C	-7.64862	-9.69208	-11.36855
C	-7.59910	-10.76716	-10.26888
C	-4.97413	-10.04263	-7.51650
C	-5.61653	-8.98486	-6.85951
C	-6.87909	-8.55242	-7.25573
C	-7.51698	-9.16914	-8.32882
C	-6.91800	-10.23399	-9.01584
C	-5.65613	-10.69863	-8.57061
C	-4.03148	-13.79660	-9.93588
C	-5.09274	-13.20084	-8.97774
C	-4.22660	-11.45753	-10.39252
H	0.54146	-13.15955	-9.15730
H	-4.38932	-14.73045	-10.39166
H	-3.30244	-3.04556	-13.17221
H	-3.22948	-0.62403	-13.05232
H	-3.57352	4.99113	-6.58050
H	-1.82947	4.68804	-6.75232
H	-2.58610	6.06133	-7.60192
H	-3.26034	1.95541	-8.88904
H	-2.04097	2.35739	-7.66263
H	-3.76291	2.45764	-7.25673
H	-2.16467	4.21059	-9.19177

H	-8.17293	5.89740	-11.43970
H	-7.87751	4.79631	-12.80790
H	-8.85604	4.25307	-11.42261
H	-4.46385	5.64330	-8.95229
H	-5.66002	3.86815	-7.73336
H	-5.44420	2.76135	-9.09229
H	-7.02943	5.38674	-9.16066
H	-7.69946	3.74727	-9.17160
H	-6.54950	3.28414	-11.33207
H	-5.38397	5.13666	-12.53111
H	-5.64016	6.19451	-11.13922
H	-0.21926	4.67025	-13.10269
H	-0.47266	6.26447	-12.35031
H	-0.38479	4.80856	-11.33219
H	-3.77940	5.68832	-13.89059
H	-2.36760	6.76149	-13.76121
H	-2.22179	5.20362	-14.60898
H	1.57911	1.35242	-11.78081
H	1.29598	3.09273	-12.00663
H	1.41772	2.40785	-10.36391
H	-0.27605	-0.14530	-10.83234
H	-0.49990	0.92626	-9.43331
H	-1.88523	0.53395	-10.48643
H	-0.96268	2.84131	-10.88745
H	-3.94545	2.72945	-16.98452
H	-5.01781	4.03892	-16.44628
H	-3.25001	4.21526	-16.30163
H	-5.39364	1.44105	-13.71405
H	-6.27947	2.39043	-14.93855
H	-5.20650	1.06237	-15.44229
H	-4.37013	3.68637	-14.09346
H	0.25353	0.16872	-13.22726
H	-0.85543	-0.37267	-15.37088
H	-2.85986	0.89521	-16.08741
H	-2.29771	5.77599	-10.38534
H	-3.40868	6.64649	-11.44436
H	-6.11555	-0.69216	-9.80259
H	-6.17295	-3.11047	-9.91018
H	-0.47229	-14.41537	-9.91568
H	0.51860	-13.33792	-10.92676
H	-0.23442	-10.77019	-9.09725
H	0.01406	-10.75964	-10.84896
H	-1.53288	-10.19994	-10.16649
H	-1.88238	-12.51134	-9.20668
H	-4.79451	-11.88085	-15.58991
H	-4.82130	-13.57567	-15.04562
H	-6.09644	-12.44981	-14.51636
H	-1.95380	-13.76080	-11.62908
H	-2.04067	-10.79906	-12.34123
H	-0.77627	-11.94546	-12.80026
H	-2.41962	-13.26161	-14.12874
H	-2.41270	-11.56814	-14.64673
H	-4.41456	-11.11832	-13.23727
H	-5.57692	-13.00872	-12.09512
H	-4.27920	-14.10189	-12.59357
H	-5.45118	-12.96815	-6.83551
H	-4.84897	-14.57349	-7.31796
H	-3.73317	-13.19003	-7.25990
H	-6.78062	-13.62542	-10.32074
H	-6.52353	-14.83149	-9.03983
H	-7.25068	-13.25694	-8.64126
H	-4.01896	-11.59770	-5.40047
H	-2.32756	-11.03443	-5.46067
H	-3.62541	-9.90994	-5.00958
H	-2.80345	-8.31505	-6.85207
H	-1.55598	-9.46161	-7.37918
H	-2.76403	-8.85965	-8.54595
H	-3.21481	-11.21216	-7.76768
H	-9.68607	-10.51080	-9.65371
H	-9.43961	-11.76053	-10.89130
H	-8.99695	-12.08218	-9.19450
H	-6.64573	-9.30721	-11.59636
H	-8.07668	-10.11380	-12.29307
H	-8.27700	-8.83817	-11.06808
H	-6.98598	-11.58889	-10.65594
H	-5.10797	-8.46773	-6.04284
H	-7.34445	-7.70497	-6.75379
H	-8.48539	-8.79192	-8.66475
H	-3.12957	-14.04856	-9.36301
C	-4.63827	-4.75021	-11.47998
C	-5.32362	-5.52091	-10.48145
C	-3.80013	-5.49643	-12.37472
H	-5.96831	-5.01982	-9.76024
H	-3.24633	-4.97436	-13.15275
C	-5.15347	-6.87096	-10.35278
C	-3.62564	-6.84937	-12.26753
H	-5.66340	-7.41471	-9.55715
H	-2.94760	-7.36710	-12.94770
C	-4.28226	-7.60358	-11.23704

3^{dimer}, triplet

N	1.57600	-5.95767	-16.44239
N	-7.81478	-2.49783	-4.25459
C	-1.41757	-5.30052	-10.92275
C	-0.67481	-5.26351	-12.09201
C	-0.06606	-4.05477	-12.52836
C	0.64568	-4.06649	-13.74045
C	1.22383	-4.24488	-14.82107
C	6.25671	-3.57127	-15.87461
C	4.57885	-3.95086	-14.03899
C	4.80840	-3.96438	-15.55627
C	0.35666	-0.78174	-17.88599
C	3.81662	-3.09341	-16.38783
C	3.35594	-1.83451	-15.63623
C	2.47441	-0.92340	-16.48868
C	1.25103	-1.67361	-17.02466
C	1.71777	-2.90552	-17.80260
C	3.10232	-7.53425	-17.62988
C	1.22766	-6.46345	-18.86785
C	3.37879	-8.98568	-14.36588
C	2.71484	-7.06795	-12.85453
C	2.65276	-7.63544	-14.28364
C	-2.20859	-6.28989	-18.00049
C	-2.15007	-4.73898	-16.00667
C	-1.33668	-5.71626	-16.87441
C	1.20883	-7.71319	-14.75903
C	0.33140	-8.59969	-14.11833
C	-1.02789	-8.61530	-14.42112
C	-1.53743	-7.70488	-15.34467
C	-0.70242	-6.79374	-16.00521
C	0.69121	-6.84759	-15.75265
C	2.61966	-3.88502	-17.00190
C	3.11727	-5.00803	-17.95328
C	2.24339	-6.26765	-17.73178
C	1.79371	-4.66265	-15.99521
C	-0.22982	-2.90064	-11.72208
C	-0.98682	-2.95455	-10.56033
C	-1.60775	-4.14663	-10.13537
C	-5.41658	-4.32765	-5.87040
C	-6.30730	-4.17672	-5.02302
C	-5.82295	-4.81594	0.06263
C	-4.54909	-4.51325	-2.08233
C	-5.93541	-4.46337	-1.42585
C	-9.50457	-7.64522	-4.97261
C	-7.03380	-5.33960	-2.10777
C	-6.45889	-6.59771	-2.77955

C	-7.53544	-7.50869	-3.36799
C	-8.42409	-6.75334	-4.36093
C	-9.03003	-5.53593	-3.66403
C	-7.99182	-4.55260	-3.05616
C	-8.60867	-0.88870	-2.51603
C	-10.26288	-2.11698	-3.90400
C	-5.40520	0.60125	-3.09769
C	-4.09471	-1.31285	-4.11047
C	-5.49744	-0.76023	-3.80111
C	-10.33589	-2.25112	-7.47757
C	-8.37937	-3.77517	-7.95283
C	-9.00361	-2.79558	-6.94212
C	-6.33714	-0.71334	-5.07021
C	-5.97179	0.17218	-6.09372
C	-6.61712	0.15408	-7.32808
C	-7.61552	-0.78793	-7.56921
C	-8.01580	-1.69462	-6.57821
C	-7.40996	-1.60786	-5.29982
C	-8.74629	-3.42337	-2.30303
C	-8.87220	-2.20662	-3.25379
C	-7.28744	-3.77707	-4.15307
H	-5.05238	-4.20696	0.56293
H	-9.73311	-3.76366	-1.95867
H	-1.91668	-6.23009	-10.64573
H	-0.57707	-6.15848	-12.70848
H	6.43992	-2.51076	-15.62986
H	6.97327	-4.17249	-15.29150
H	6.48772	-3.70979	-16.94373
H	3.54401	-4.19939	-13.77275
H	5.23738	-4.69060	-13.55560
H	4.81538	-2.96834	-13.60045
H	4.68110	-5.01161	-15.87720
H	0.88944	-0.44920	-18.79365
H	-0.55217	-1.31601	-18.20845
H	0.04282	0.12082	-17.33804
H	4.37619	-2.73689	-17.27344
H	4.24232	-1.27996	-15.28622
H	2.79451	-2.12707	-14.73648
H	3.05403	-0.53053	-17.34587
H	2.15564	-0.04637	-15.89996
H	0.66249	-2.02655	-16.15906
H	0.84641	-3.45517	-18.19413
H	2.29110	-2.56191	-18.68407
H	2.48607	-8.41063	-17.37911
H	3.59215	-7.72855	-18.59673
H	3.88515	-7.42912	-16.86691
H	0.63023	-5.55914	-19.04263
H	1.75803	-6.70509	-19.80194
H	0.54468	-7.29472	-18.64124
H	2.93555	-9.72310	-13.67711
H	3.33923	-9.41112	-15.37969
H	4.43820	-8.87027	-14.08450
H	2.21986	-7.74088	-12.13565
H	3.76275	-6.94445	-12.53617
H	2.22000	-6.08906	-12.79416
H	3.17592	-6.92236	-14.93358
H	-3.08485	-6.82302	-17.59800
H	-2.58729	-5.47875	-18.64355
H	-1.65166	-6.99524	-18.63454
H	-1.54652	-4.34363	-15.17835
H	-2.50444	-3.88947	-16.61309
H	-3.03631	-5.23489	-15.57954
H	-0.52499	-5.13814	-17.33060
H	0.71662	-9.27260	-13.34912
H	-1.69344	-9.31014	-13.90521
H	-2.61181	-7.68316	-15.54054
H	4.16546	-5.25283	-17.73901
H	3.07705	-4.68157	-19.00187
H	0.22490	-1.95957	-12.03442
H	-1.11222	-2.04763	-9.96775
H	-6.77807	-4.65543	0.58911
H	-5.54467	-5.87580	0.19573
H	-3.88549	-3.77421	-1.60481
H	-4.07580	-5.50069	-1.96144
H	-4.58779	-4.28429	-3.15442
H	-6.26391	-3.41243	-1.48524
H	-9.06224	-8.51298	-5.48727
H	-10.18457	-8.03131	-4.19354
H	-10.11575	-7.09119	-5.70441
H	-7.69889	-5.69766	-1.29954
H	-5.76975	-6.30177	-3.58424
H	-5.85726	-7.15617	-2.04330
H	-8.17261	-7.91261	-2.55825
H	-7.06640	-8.37782	-3.85912
H	-7.78231	-6.37801	-5.17748
H	-9.67915	-4.98855	-4.36531
H	-9.68402	-5.88976	-2.84504
H	-8.59327	-0.04034	-3.21706
H	-9.41033	-0.70932	-1.78288
H	-7.65383	-0.91155	-1.97496
H	-10.54455	-3.05038	-4.40773
H	-11.02034	-1.90465	-3.13362
H	-10.29273	-1.30244	-4.64204
H	-6.39974	1.01889	-2.88089
H	-4.85822	0.50595	-2.14554
H	-4.86231	1.33606	-3.71404
H	-3.53359	-0.62967	-4.76859
H	-3.51668	-1.43793	-3.18036
H	-4.15429	-2.28862	-4.61147
H	-5.97597	-1.47060	-3.11485
H	-10.19920	-1.73422	-8.44115
H	-11.04735	-3.07581	-7.64693
H	-10.79998	-1.53774	-6.78052
H	-7.41639	-4.16173	-7.59219
H	-9.05372	-4.63014	-8.12487
H	-8.21097	-3.28582	-8.92564
H	-9.21057	-3.36675	-6.02972
H	-5.14747	0.86945	-5.92906
H	-6.31765	0.85216	-8.11226
H	-8.08195	-0.83548	-8.55582
H	-8.18338	-3.12238	-1.40981
C	-2.50408	-4.17757	-8.96464
C	-3.30472	-3.06330	-8.64064
C	-2.66178	-5.34440	-8.18945
H	-3.23254	-2.15452	-9.23931
H	-2.04520	-6.21722	-8.40817
C	-4.24664	-3.12037	-7.62539
C	-3.59131	-5.41661	-7.16249
H	-4.88518	-2.25955	-7.42131
H	-3.70049	-6.33747	-6.58912
C	-4.42398	-4.30903	-6.86515
N	-2.62237	3.57764	-12.31375
N	-4.72772	-11.81515	-9.06901
C	-3.78180	-2.73377	-12.38154
C	-3.69601	-1.35568	-12.33455
C	-4.60014	-0.59570	-11.53435
C	-4.44234	0.78756	-11.45643
C	-4.18662	2.00188	-11.44721
C	-3.30850	4.99819	-7.33678
C	-3.38996	2.60683	-8.10383
C	-3.41720	4.07309	-8.55548
C	-8.05928	4.50842	-12.25178
C	-4.64239	4.47532	-9.43665
C	-5.89089	3.62963	-9.13985
C	-7.11899	4.07628	-9.93054
C	-6.84412	4.06492	-11.43716

C	-5.62700	4.94475	-11.73124
C	-0.81900	5.28461	-12.05658
C	-2.60959	5.62890	-13.74834
C	0.99360	2.47961	-10.77608
C	-0.83234	0.86252	-10.10901
C	-0.48407	2.10228	-10.95223
C	-3.44588	3.28517	-16.31402
C	-4.78229	1.60096	-14.98434
C	-3.68764	2.68065	-14.92271
C	-0.86522	1.86333	-12.40602
C	-0.20919	0.84855	-13.11589
C	-0.63003	0.46939	-14.38787
C	-1.73382	1.09538	-14.96318
C	-2.41400	2.12624	-14.29928
C	-1.93889	2.54286	-13.03105
C	-4.33297	4.55473	-10.96507
C	-3.22204	5.59853	-11.26585
C	-2.29718	5.02519	-12.36922
C	-3.73710	3.28626	-11.54538
C	-5.61066	-1.30163	-10.82421
C	-5.67120	-2.68130	-10.87157
C	-4.75180	-3.44416	-11.63277
C	-4.28058	-9.10294	-11.13278
C	-4.20460	-10.30572	-10.83671
C	0.01908	-13.27161	-10.75104
C	-0.78231	-10.89033	-10.73382
C	-1.20747	-12.36057	-10.61555
C	-5.80009	-12.94821	-14.35158
C	-2.32907	-12.80861	-11.60568
C	-2.30298	-12.01286	-12.92022
C	-3.33407	-12.49479	-13.93804
C	-4.74865	-12.46709	-13.35198
C	-4.77647	-13.30292	-12.07068
C	-3.75914	-12.86633	-10.98054
C	-4.14581	-13.47119	-7.29213
C	-6.14589	-13.84606	-8.71958
C	-2.45924	-10.65589	-5.88643
C	-2.15127	-9.10942	-7.86306
C	-2.92882	-10.31658	-7.30804
C	-8.81084	-11.45532	-9.03233
C	-7.78265	-9.84322	-10.68393
C	-7.49648	-10.88987	-9.59233
C	-4.42472	-10.05029	-7.39001
C	-4.96108	-8.99515	-6.64038
C	-6.28297	-8.59190	-6.80636
C	-7.08866	-9.23966	-7.73975
C	-6.60369	-10.31113	-8.50291
C	-5.27231	-10.74573	-8.28664
C	-3.82135	-13.86227	-9.78985
C	-4.72828	-13.25134	-8.69226
C	-4.20590	-11.57168	-10.32842
H	0.82469	-12.96531	-10.06389
H	-4.19312	-14.84541	-10.11100
H	-3.03273	-3.28490	-12.95147
H	-2.90800	-0.83404	-12.88080
H	-4.21547	4.93169	-6.71117
H	-2.44893	4.72734	-6.70188
H	-3.19011	6.05221	-7.63739
H	-3.48545	1.90900	-8.94504
H	-2.43732	2.39049	-7.59447
H	-4.19731	2.38911	-7.38756
H	-2.50754	4.22443	-9.15983
H	-8.34684	5.54475	-12.00229
H	-7.85321	4.47010	-13.33411
H	-8.93024	3.86334	-12.05203
H	-4.90237	5.51583	-9.16551
H	-6.10431	3.66570	-8.05886
H	-5.68231	2.57779	-9.38346
H	-7.41183	5.09971	-9.62690
H	-7.97613	3.42266	-9.69479
H	-6.59377	3.02981	-11.72765
H	-5.41837	4.94602	-12.81326
H	-5.87728	5.98786	-11.46038
H	-0.16650	4.77726	-12.78326
H	-0.61335	6.36492	-12.11096
H	-0.55209	4.93958	-11.04921
H	-3.66895	5.51916	-14.01438
H	-2.37005	6.70359	-13.74538
H	-2.00368	5.14867	-14.52986
H	1.65657	1.65274	-11.07755
H	1.26682	3.35986	-11.37652
H	1.21053	2.70399	-9.71896
H	-0.18665	0.01230	-10.37883
H	-0.68413	1.06687	-9.03674
H	-1.87753	0.55791	-10.26148
H	-1.09975	2.93089	-10.57977
H	-3.13893	2.51245	-17.03720
H	-4.36938	3.74765	-16.69936
H	-2.66009	4.05506	-16.29754
H	-4.97408	1.17130	-13.99194
H	-5.72343	2.03355	-15.36241
H	-4.49608	0.77930	-15.66080
H	-4.05992	3.47485	-14.26542
H	0.62886	0.32383	-12.65221
H	-0.12084	-0.34027	-14.91268
H	-2.09186	0.75941	-15.93891
H	-2.62787	5.79098	-10.36289
H	-3.65424	6.56123	-11.57315
H	-6.32338	-0.74176	-10.21757
H	-6.44268	-3.19411	-10.29650
H	-0.23099	-14.32321	-10.53442
H	0.42630	-13.23149	-11.77618
H	-0.07006	-10.64110	-9.93143
H	-0.27607	-10.69004	-11.69111
H	-1.63325	-10.20294	-10.65273
H	-1.59534	-12.48764	-9.59120
H	-5.79129	-12.33300	-15.26595
H	-5.61369	-13.99442	-14.65072
H	-6.81430	-12.89866	-13.92206
H	-2.11601	-13.86061	-11.87345
H	-2.49628	-10.95266	-12.70296
H	-1.29121	-12.06362	-13.35524
H	-3.09706	-13.52835	-14.25552
H	-3.28501	-11.86972	-14.84619
H	-4.98344	-11.42306	-13.08109
H	-5.79113	-13.29447	-11.64185
H	-4.55734	-14.35485	-12.33421
H	-4.72942	-12.92955	-6.53234
H	-4.17643	-14.54331	-7.04376
H	-3.10149	-13.13886	-7.23212
H	-6.60849	-13.76055	-9.71135
H	-6.10652	-14.91381	-8.45346
H	-6.79340	-13.33875	-7.99043
H	-2.99997	-11.51850	-5.47007
H	-1.38240	-10.89118	-5.88250
H	-2.61163	-9.80598	-5.20182
H	-2.25277	-8.23842	-7.19701
H	-1.07801	-9.34254	-7.94612
H	-2.52020	-8.82201	-8.85800
H	-2.70821	-11.17186	-7.95916
H	-9.43372	-10.65768	-8.59598
H	-9.39870	-11.92957	-9.83527
H	-8.63576	-12.20637	-8.24745
H	-6.85156	-9.44455	-11.10782
H	-8.36953	-10.29537	-11.50043
H	-8.36368	-8.99613	-10.28490

H	-6.94758	-11.70922	-10.07105
H	-4.32341	-8.45579	-5.93699
H	-6.66999	-7.74585	-6.23824
H	-8.11023	-8.88828	-7.89947
H	-2.81665	-14.02166	-9.37640
C	-4.73140	-4.90335	-11.57100
C	-5.17452	-5.58845	-10.41331
C	-4.18182	-5.68410	-12.61695
H	-5.56848	-5.02101	-9.56947
H	-3.84235	-5.19186	-13.52844
C	-5.03230	-6.95564	-10.28156
C	-4.04754	-7.05523	-12.51059
H	-5.33441	-7.45342	-9.35894
H	-3.60805	-7.62710	-13.32786
C	-4.44691	-7.73212	-11.32525

3^{dimer}, quintet

N	1.56899	-5.94484	-16.44565
N	-7.82628	-2.46880	-4.27065
C	-1.44345	-5.28579	-10.93607
C	-0.70285	-5.24826	-12.10706
C	-0.05713	-4.05046	-12.52058
C	0.65550	-4.06176	-13.73199
C	1.23431	-4.24117	-14.81195
C	6.27657	-3.59811	-15.85152
C	4.59293	-3.98671	-14.02335
C	4.82557	-3.98693	-15.54023
C	0.40177	-0.75306	-17.85314
C	3.84018	-3.10308	-16.36569
C	3.38714	-1.84744	-15.60395
C	2.51304	-0.92230	-16.44904
C	1.28631	-1.65885	-16.99625
C	1.74658	-2.88754	-17.78314
C	3.08301	-7.52993	-17.63734
C	1.22414	-6.43207	-18.87567
C	3.33960	-8.99303	-14.36997
C	2.69009	-7.07243	-12.85607
C	2.62610	-7.63612	-14.28659
C	-2.21834	-6.24092	-18.01077
C	-2.13922	-4.69229	-16.01522
C	-1.33915	-5.67865	-16.88480
C	1.18281	-7.70013	-14.76583
C	0.29594	-8.57898	-14.12765
C	-1.06244	-8.58394	-14.43471
C	-1.56155	-7.66918	-15.35984
C	-0.71693	-6.76405	-16.01668
C	0.67550	-6.83007	-15.76076
C	2.63808	-3.88063	-16.98729
C	3.12606	-5.00222	-17.94513
C	2.23768	-6.25392	-17.73473
C	1.80187	-4.65656	-15.98793
C	-0.18260	-2.90737	-11.69174
C	-0.93956	-2.96058	-10.52997
C	-1.59540	-4.14115	-10.12757
C	-5.40439	-4.28883	-5.86500
C	-6.29779	-4.13595	-5.02117
C	-5.82613	-4.72646	0.07663
C	-4.54525	-4.41761	-2.06331
C	-5.93577	-4.38519	-1.41466
C	-9.43638	-7.64024	-4.96347
C	-7.01688	-5.28257	-2.09688
C	-6.41925	-6.53610	-2.75758
C	-7.47802	-7.46693	-3.34781
C	-8.37262	-6.73023	-4.34933
C	-9.00132	-5.51854	-3.66206
C	-7.98115	-4.51590	-3.05551
C	-8.63830	-0.85627	-2.54384
C	-10.27872	-2.10786	-3.92750
C	-5.45170	0.67684	-3.16983
C	-4.11593	-1.24037	-4.14278
C	-5.52589	-0.69921	-3.84669
C	-10.35418	-2.29046	-7.48848
C	-8.39330	-3.81374	-7.94674
C	-9.01697	-2.81969	-6.94978
C	-6.36396	-0.68570	-5.11765
C	-5.99980	0.17806	-6.15989
C	-6.63978	0.12826	-7.39600
C	-7.63501	-0.82182	-7.61782
C	-8.03411	-1.70754	-6.60770
C	-7.43158	-1.59053	-5.33000
C	-8.75521	-3.39118	-2.31529
C	-8.88875	-2.18101	-3.27409
C	-7.28316	-3.73992	-4.15578
H	-5.06782	-4.10260	0.57729
H	-9.74072	-3.74163	-1.97744
H	-1.96829	-6.20538	-10.67276
H	-0.63358	-6.13352	-12.74115
H	6.46462	-2.54090	-15.59640
H	6.98865	-4.20835	-15.27233
H	6.50939	-3.72790	-16.92133
H	3.55633	-4.23225	-13.76141
H	5.24669	-4.73437	-13.54572
H	4.83373	-3.00966	-13.57501
H	4.69394	-5.03037	-15.87160
H	0.94147	-0.41404	-18.75427
H	-0.50816	-1.27896	-18.18612
H	0.08937	0.14515	-17.29735
H	4.40312	-2.74333	-17.24787
H	4.27682	-1.30215	-15.24784
H	2.82250	-2.14405	-14.70758
H	3.09719	-0.52344	-17.30035
H	2.19777	-0.05021	-15.85114
H	0.69220	-2.01504	-16.13600
H	0.87239	-3.42720	-18.18211
H	2.32571	-2.54127	-18.65977
H	2.45659	-8.40158	-17.39549
H	3.57551	-7.72267	-18.60315
H	3.86309	-7.43790	-16.86990
H	0.63793	-5.51997	-19.04804
H	1.75532	-6.67486	-19.80900
H	0.53070	-7.25657	-18.65613
H	2.88723	-9.72759	-13.68411
H	3.29873	-9.41591	-15.38476
H	4.39930	-8.88831	-14.08547
H	2.18993	-7.74379	-12.13935
H	3.73839	-6.95659	-12.53617
H	2.20148	-6.09053	-12.79398
H	3.15749	-6.92659	-14.93384
H	-3.10105	-6.76370	-17.60875
H	-2.58724	-5.42496	-18.65339
H	-1.66981	-6.95259	-18.64522
H	-1.52799	-4.30027	-15.19099
H	-2.49001	-3.84128	-16.62164
H	-3.02656	-5.17995	-15.58116
H	-0.52111	-5.10996	-17.34130
H	0.67291	-9.25418	-13.35651
H	-1.73439	-9.27377	-13.91977
H	-2.63493	-7.63797	-15.56004
H	4.17089	-5.26012	-17.72987
H	3.09227	-4.66792	-18.99147
H	0.30421	-1.97556	-11.98383
H	-1.03738	-2.06301	-9.91835
H	-6.78658	-4.57728	0.59670
H	-5.53241	-5.78106	0.21812
H	-3.89600	-3.66502	-1.58720
H	-4.05719	-5.39663	-1.93322

H	-4.58171	-4.19642	-3.13712
H	-6.28010	-3.33988	-1.48257
H	-8.97880	-8.50022	-5.47765
H	-10.11206	-8.03737	-4.18620
H	-10.05463	-7.09602	-5.69680
H	-7.68108	-5.64555	-1.29012
H	-5.72921	-6.23474	-3.55935
H	-5.81420	-7.08101	-2.01402
H	-8.11309	-7.87765	-2.53983
H	-6.99252	-8.33038	-3.83316
H	-7.73037	-6.34980	-5.16290
H	-9.65552	-4.98519	-4.36955
H	-9.65375	-5.87785	-2.84420
H	-8.62804	-0.01247	-3.25044
H	-9.44342	-0.67866	-1.81404
H	-7.68474	-0.86757	-2.00015
H	-10.55246	-3.04819	-4.42273
H	-11.03891	-1.89429	-3.16020
H	-10.31370	-1.30040	-4.67306
H	-6.45177	1.08516	-2.96094
H	-4.90349	0.60772	-2.21610
H	-4.91897	1.40665	-3.80085
H	-3.56058	-0.56187	-4.81055
H	-3.53936	-1.34286	-3.20900
H	-4.16276	-2.22512	-4.62735
H	-5.99712	-1.40208	-3.14790
H	-10.22324	-1.78728	-8.46010
H	-11.06198	-3.12125	-7.64290
H	-10.81954	-1.56896	-6.80075
H	-7.42030	-4.18023	-7.59169
H	-9.05914	-4.68074	-8.08935
H	-8.24618	-3.34553	-8.93355
H	-9.21729	-3.37635	-6.02713
H	-5.17860	0.88194	-6.00887
H	-6.33526	0.80546	-8.19640
H	-8.09852	-0.89450	-8.60421
H	-8.20394	-3.07859	-1.41885
C	-2.49233	-4.16399	-8.95735
C	-3.29960	-3.04956	-8.65157
C	-2.64458	-5.32217	-8.16868
H	-3.23066	-2.14954	-9.26427
H	-2.02465	-6.19455	-8.37833
C	-4.24234	-3.09803	-7.63659
C	-3.57370	-5.38468	-7.14060
H	-4.88703	-2.23895	-7.44530
H	-3.67994	-6.29540	-6.55006
C	-4.41230	-4.27700	-6.86006
N	-2.63031	3.56633	-12.28293
N	-4.71169	-11.82382	-9.07937
C	-3.74266	-2.75721	-12.34078
C	-3.66090	-1.37428	-12.28578
C	-4.59806	-0.62386	-11.52479
C	-4.44732	0.76934	-11.43916
C	-4.19327	1.98067	-11.42509
C	-3.35845	4.97279	-7.29012
C	-3.38227	2.59537	-8.10276
C	-3.43605	4.06879	-8.52690
C	-8.07949	4.47175	-12.22404
C	-4.66111	4.46109	-9.41160
C	-5.90347	3.60701	-9.11361
C	-7.13541	4.04338	-9.90397
C	-6.86096	4.03484	-11.41088
C	-5.64901	4.92206	-11.70498
C	-0.83550	5.27917	-12.01104
C	-2.61234	5.61699	-13.71847
C	0.98710	2.48645	-10.74587
C	-0.82758	0.85707	-10.07991
C	-0.48790	2.09900	-10.92284

C	-3.44148	3.28141	-16.28980
C	-4.77473	1.58908	-14.96839
C	-3.68484	2.67320	-14.90032
C	-0.86640	1.85957	-12.37692
C	-0.20418	0.84963	-13.08794
C	-0.61952	0.47365	-14.36274
C	-1.72426	1.09690	-14.93911
C	-2.41087	2.12256	-14.27385
C	-1.94266	2.53554	-13.00159
C	-4.35212	4.53776	-10.94052
C	-3.24663	5.58745	-11.24249
C	-2.31009	5.01476	-12.33651
C	-3.74883	3.27227	-11.51779
C	-5.63598	-1.32755	-10.86359
C	-5.69778	-2.71146	-10.92411
C	-4.74645	-3.46219	-11.64512
C	-4.27910	-9.13780	-11.17660
C	-4.20603	-10.33557	-10.87363
C	-0.00413	-13.33174	-10.80262
C	-0.78000	-10.94260	-10.82788
C	-1.21986	-12.40532	-10.67428
C	-5.87235	-13.02403	-14.33002
C	-2.35691	-12.86232	-11.64261
C	-2.34818	-12.08436	-12.96847
C	-3.39843	-12.57505	-13.96246
C	-4.80312	-12.53480	-13.35349
C	-4.81243	-13.35509	-12.06187
C	-3.77740	-12.90552	-10.99399
C	-4.11328	-13.46141	-7.29063
C	-6.13368	-13.84441	-8.68602
C	-2.40000	-10.63947	-5.93537
C	-2.10356	-9.12791	-7.94101
C	-2.88429	-10.31833	-7.35625
C	-8.79196	-11.44520	-8.99014
C	-7.78192	-9.85136	-10.67044
C	-7.48386	-10.88978	-9.57415
C	-4.37865	-10.04264	-7.42311
C	-4.89899	-8.97684	-6.67709
C	-6.22145	-8.56887	-6.82901
C	-7.04232	-9.22315	-7.74461
C	-6.57307	-10.30467	-8.50333
C	-5.24039	-10.74276	-8.30260
C	-3.82397	-13.88380	-9.78771
C	-4.71338	-13.25514	-8.68545
C	-4.20532	-11.60098	-10.35167
H	0.81171	-13.01962	-10.13027
H	-4.20170	-14.87123	-10.08824
H	-2.97111	-3.31008	-12.87879
H	-2.85252	-0.84985	-12.79842
H	-4.27010	4.87700	-6.67524
H	-2.50044	4.70637	-6.65128
H	-3.25719	6.03431	-7.56986
H	-3.45420	1.91205	-8.95786
H	-2.43149	2.38914	-7.58587
H	-4.19362	2.34669	-7.40168
H	-2.52428	4.25122	-9.11944
H	-8.37261	5.50627	-11.97352
H	-7.87437	4.43536	-13.30662
H	-8.94665	3.82170	-12.02372
H	-4.92751	5.50071	-9.14300
H	-6.11698	3.64085	-8.03250
H	-5.68673	2.55683	-9.35667
H	-7.43678	5.06420	-9.59998
H	-7.98692	3.38237	-9.66808
H	-6.60489	3.00138	-11.70236
H	-5.44075	4.92561	-12.78717
H	-5.90548	5.96345	-11.43292
H	-0.17519	4.77482	-12.73277

H	-0.63289	6.36021	-12.06240
H	-0.57599	4.93371	-11.00179
H	-3.66906	5.50374	-13.99341
H	-2.37654	6.69251	-13.71345
H	-1.99799	5.13886	-14.49461
H	1.65590	1.66467	-11.04841
H	1.25418	3.36947	-11.34495
H	1.20232	2.71093	-9.68841
H	-0.17782	0.00995	-10.34972
H	-0.67893	1.06264	-9.00797
H	-1.87128	0.54641	-10.23038
H	-1.10977	2.92291	-10.55005
H	-3.13116	2.51091	-17.01392
H	-4.36532	3.74236	-16.67615
H	-2.65771	4.05314	-16.27022
H	-4.96859	1.15735	-13.97739
H	-5.71612	2.01844	-15.34951
H	-4.48235	0.76982	-15.64512
H	-4.06291	3.46415	-14.24242
H	0.63529	0.32599	-12.62516
H	-0.10395	-0.33039	-14.88993
H	-2.07800	0.76254	-15.91698
H	-2.65995	5.79178	-10.33729
H	-3.68441	6.54446	-11.55954
H	-6.37470	-0.77011	-10.28680
H	-6.49222	-3.23120	-10.38645
H	-0.26258	-14.37592	-10.56138
H	0.39271	-13.31750	-11.83256
H	-0.06266	-10.68262	-10.03345
H	-0.27351	-10.77134	-11.79084
H	-1.62300	-10.24386	-10.76016
H	-1.59849	-12.50597	-9.64366
H	-5.87647	-12.42017	-15.25198
H	-5.69434	-14.07451	-14.61914
H	-6.87924	-12.96573	-13.88462
H	-2.15136	-13.91833	-11.90026
H	-2.53176	-11.01961	-12.76481
H	-1.34402	-12.14760	-13.41937
H	-3.17085	-13.61356	-14.27060
H	-3.36237	-11.96182	-14.87929
H	-5.02974	-11.48662	-13.09140
H	-5.82012	-13.33945	-11.61716
H	-4.59966	-14.41066	-12.31634
H	-4.68485	-12.90897	-6.52943
H	-4.14471	-14.53052	-7.02966
H	-3.06701	-13.13251	-7.24791
H	-6.61033	-13.76732	-9.67175
H	-6.09489	-14.90948	-8.40917
H	-6.76844	-13.32674	-7.95295
H	-2.94437	-11.48977	-5.49881
H	-1.32565	-10.88591	-5.94103
H	-2.53484	-9.77729	-5.26248
H	-2.19677	-8.24155	-7.29430
H	-1.03214	-9.36980	-8.02360
H	-2.47521	-8.86083	-8.94056
H	-2.67899	-11.18546	-7.99651
H	-9.40578	-10.64137	-8.55223
H	-9.39297	-11.92467	-9.78014
H	-8.60809	-12.18966	-8.20101
H	-6.85512	-9.45973	-11.11000
H	-8.38137	-10.30821	-11.47512
H	-8.35475	-8.99912	-10.27041
H	-6.94469	-11.71520	-10.05345
H	-4.24848	-8.43369	-5.98805
H	-6.59978	-7.71403	-6.26734
H	-8.06452	-8.86910	-7.89377
H	-2.81355	-14.03841	-9.38651
C	-4.72751	-4.93519	-11.59039
C	-5.10248	-5.61004	-10.41056
C	-4.24336	-5.70935	-12.66440
H	-5.44599	-5.03713	-9.54832
H	-3.95531	-5.21444	-13.59271
C	-4.96439	-6.98325	-10.28877
C	-4.10514	-7.08568	-12.56335
H	-5.22365	-7.48062	-9.35316
H	-3.70953	-7.66037	-13.40073
C	-4.44307	-7.75580	-11.36141

Truncated 2^{dimer}, singlet

N	2.86589	-6.47388	-14.51474
N	-5.17263	-1.18312	-4.17256
C	-1.35755	-5.75888	-9.97480
C	-0.47273	-5.71125	-11.00719
C	0.08382	-4.44849	-11.46695
C	1.01021	-4.47289	-12.47679
C	1.88681	-4.64516	-13.35748
C	4.73248	-3.39890	-14.12502
C	3.31573	-3.62506	-16.22281
C	4.72441	-8.06033	-15.04927
C	3.31478	-7.15902	-16.88239
C	1.92976	-7.39011	-13.93908
C	3.89653	-4.38449	-15.00032
C	4.77472	-5.55854	-15.51320
C	3.91296	-6.84117	-15.50071
C	2.80972	-5.12023	-14.22687
C	-0.36755	-3.25670	-10.81671
C	-1.28428	-3.29184	-9.78327
C	-3.94903	-3.35410	-6.48538
C	-4.58633	-3.11290	-5.43115
C	-4.86986	-4.37928	-2.49822
C	-7.17933	-3.72853	-3.36792
C	-5.73707	-3.22398	-3.09017
C	-5.02073	0.37893	-2.21850
C	-7.24092	-0.19188	-3.16344
H	-4.93041	0.73623	-4.93598
C	-4.71038	-0.30609	-5.20263
C	-5.81081	-2.03417	-2.09460
C	-5.81931	-0.72815	-2.91567
C	-5.12938	-2.56110	-4.31996
H	-6.68163	-2.10523	-1.42665
H	-1.76735	-6.71803	-9.64756
H	-0.15725	-6.62847	-11.50898
H	5.54508	-2.95941	-14.72512
H	2.73825	-4.29333	-16.87641
H	4.13182	-3.17915	-16.81440
H	4.11895	-8.98007	-15.05799
H	5.57301	-8.21802	-15.73315
H	5.12140	-7.91566	-14.03233
H	2.72872	-6.31646	-17.27541
H	4.11559	-7.39004	-17.60240
H	2.64807	-8.03474	-16.82708
H	5.62356	-5.70058	-14.82584
H	5.19415	-5.35762	-16.50954
H	0.00943	-2.29227	-11.16516
H	-5.31895	-4.74297	-1.56024
H	-7.81910	-2.92752	-3.76066
H	-7.63203	-4.11188	-2.43904
H	-5.04975	1.32374	-2.78301
H	-5.44967	0.57517	-1.22348
H	-3.96763	0.08393	-2.08928
H	-7.88214	-0.93896	-3.65017

H	-7.71146	0.10032	-2.21142
H	-7.21320	0.69886	-3.81166
H	-4.91097	-2.04025	-1.45923
C	-1.77992	-2.09201	-9.15589
C	-1.80355	-4.56423	-9.29793
H	-1.43557	-1.12833	-9.54059
C	-2.66963	-2.13760	-8.12969
C	-2.69092	-4.60034	-8.23751
H	-3.03683	-1.21564	-7.67525
H	-3.06701	-5.56327	-7.88619
C	-3.14192	-3.40368	-7.59359
N	-2.88839	2.19633	-14.10700
N	-6.34754	-10.64398	-9.06609
C	-3.14259	-3.89879	-12.78745
C	-3.06683	-2.54266	-12.80948
C	-3.85948	-1.73196	-11.90456
C	-3.80954	-0.37193	-12.04809
C	-3.77737	0.83540	-12.37975
C	-4.34479	3.90406	-11.28378
C	-5.99350	3.03097	-12.98009
C	-1.46473	4.22860	-14.39038
C	-3.12070	3.62723	-16.14111
C	-2.25723	1.05224	-14.69741
C	-4.48418	3.30860	-12.72171
C	-3.96243	4.28035	-13.82140
C	-2.83753	3.58152	-14.63370
C	-3.69106	2.03848	-12.99309
C	-4.69296	-2.40940	-10.95968
C	-4.77493	-3.78804	-10.92626
C	-4.96743	-8.01558	-10.75628
C	-5.12761	-9.23810	-10.53716
C	-3.45946	-11.97130	-10.81650
C	-5.71723	-12.13041	-11.96894
C	-4.99913	-11.82841	-10.62487
C	-6.35957	-12.27761	-7.18382
C	-8.01441	-12.51236	-9.02665
H	-6.32441	-8.80291	-8.04681
C	-7.02943	-9.51101	-8.51810
C	-5.52570	-12.80069	-9.53417
C	-6.58698	-12.05733	-8.68448
C	-5.44505	-10.47636	-10.09899
H	-5.93509	-13.72239	-9.97169
H	-2.52927	-4.49309	-13.46944
H	-2.40294	-2.03557	-13.51241
H	-4.95348	4.81774	-11.19949
H	-6.14747	2.62765	-13.99286
H	-6.57537	3.96217	-12.88136
H	-0.66889	3.67545	-14.91493
H	-1.45736	5.26652	-14.75901
H	-1.22132	4.23865	-13.31619
H	-4.07509	3.13181	-16.37963
H	-3.18036	4.67354	-16.47899
H	-2.32370	3.13776	-16.72196
H	-3.58314	5.21186	-13.37528
H	-4.78368	4.56758	-14.49298
H	-5.27878	-1.81749	-10.25331
H	-3.21367	-12.99424	-11.14300
H	-6.80993	-12.06909	-11.85674
H	-5.45968	-13.14322	-12.32027
H	-7.11127	-11.74725	-6.57795
H	-6.43382	-13.34977	-6.94236
H	-5.36177	-11.92197	-6.88320
H	-8.22416	-12.39786	-10.10081
H	-8.15025	-13.57188	-8.75909
H	-8.76315	-11.92646	-8.46983
H	-4.69175	-13.09953	-8.88041
C	-5.61895	-4.48715	-9.98813
C	-3.99465	-4.59372	-11.85664
H	-6.19997	-3.89423	-9.27666
C	-5.68103	-5.84437	-9.95669
C	-4.06297	-5.97259	-11.81533
H	-6.31608	-6.35558	-9.22999
H	-3.45411	-6.56158	-12.50435
C	-4.88940	-6.65433	-10.86816
H	2.64239	-2.81746	-15.89445
H	4.09569	-2.58485	-13.74560
H	0.88781	-7.08825	-14.14846
H	2.08701	-8.39675	-14.34927
H	-6.38327	2.29432	-12.26056
H	-4.69255	3.17662	-10.53435
H	-3.29657	4.15461	-11.05699
H	-1.60525	0.53521	-13.97165
H	-3.00243	0.31553	-15.04763
H	-1.63965	1.36115	-15.55175
H	-3.62083	-0.40014	-5.36113
H	-5.19816	-0.52669	-6.16919
H	-7.17098	-4.54142	-4.11142
H	-4.80774	-5.21979	-3.20689
H	-3.84731	-4.02915	-2.29223
H	-7.57680	-8.95364	-9.29943
H	-2.92830	-11.76199	-9.87540
H	-3.09832	-11.26311	-11.57819
H	-5.41797	-11.40505	-12.74127
H	5.17028	-3.92140	-13.26117
H	2.03590	-7.44203	-12.84030
H	-7.74899	-9.83921	-7.75497

Truncated 2^{dimer}, triplet

N	2.87589	-6.46206	-14.55781
N	-5.12343	-1.19303	-4.17423
C	-1.32898	-5.75718	-9.99909
C	-0.44857	-5.70731	-11.03512
C	0.10498	-4.44342	-11.49540
C	1.02712	-4.46558	-12.50918
C	1.90014	-4.63583	-13.39380
H	2.63171	-2.81465	-15.93055
C	3.31611	-3.61040	-16.26365
C	4.73347	-8.04615	-15.10246
C	3.31537	-7.14341	-16.92832
H	2.05534	-7.43530	-12.88170
H	0.89994	-7.07714	-14.18217
C	1.94297	-7.37991	-13.97954
C	3.90270	-4.37109	-15.04472
C	4.77972	-5.54365	-15.56298
C	3.91910	-6.82703	-15.54871
C	2.81978	-5.10886	-14.26775
C	-0.34466	-3.25295	-10.84155
C	-1.25699	-3.29036	-9.80431
C	-3.90777	-3.35963	-6.49531
C	-4.54081	-3.12049	-5.43805
C	-7.12458	-3.74129	-3.36481
C	-5.68158	-3.23592	-3.09241
C	-4.96462	0.36635	-2.21858
C	-7.18830	-0.20500	-3.15497
H	-3.57737	-0.40632	-5.36756
H	-5.15748	-0.53434	-6.16990

C	-4.66627	-0.31412	-5.20497
C	-5.75214	-2.04760	-2.09482
C	-5.76520	-0.74041	-2.91397
C	-5.07965	-2.57075	-4.32379
H	-6.61991	-2.12143	-1.42346
H	-1.73694	-6.71710	-9.67181
H	-0.13462	-6.62352	-11.53970
H	2.75001	-4.27459	-16.93213
H	4.13063	-3.14881	-16.84510
H	4.12822	-8.96598	-15.11040
H	5.57941	-8.20230	-15.78997
H	5.13430	-7.90284	-14.08687
H	2.72497	-6.30159	-17.31631
H	4.11330	-7.37077	-17.65267
H	2.65153	-8.02112	-16.87124
H	5.63210	-5.68453	-14.87856
H	5.19515	-5.33993	-16.56025
H	0.02972	-2.28766	-11.19040
H	-7.78192	-2.94204	-3.73299
H	-7.56101	-4.14628	-2.43745
H	-4.99794	1.31168	-2.78183
H	-5.38917	0.56062	-1.22130
H	-3.91058	0.07250	-2.09494
H	-7.83020	-0.95153	-3.64154
H	-7.65573	0.08441	-2.20057
H	-7.16314	0.68738	-3.80093
H	-4.84923	-2.05556	-1.46230
C	-1.75100	-2.09185	-9.17312
C	-1.77313	-4.56389	-9.31862
H	-1.40980	-1.12731	-9.55845
C	-2.63634	-2.13968	-8.14324
C	-2.65600	-4.60228	-8.25452
H	-3.00296	-1.21867	-7.68642
H	-3.03014	-5.56601	-7.90334
C	-3.10529	-3.40693	-7.60699
N	-2.88395	2.20266	-14.11332
N	-6.31096	-10.64782	-9.07640
C	-3.12743	-3.89456	-12.80150
C	-3.05291	-2.53834	-12.82190
C	-3.84243	-1.72962	-11.91248
C	-3.79425	-0.36934	-12.05425
C	-3.76449	0.83849	-12.38430
C	-4.32988	3.90509	-11.28153
C	-5.98499	3.03303	-12.97213
C	-1.46321	4.23654	-14.39976
C	-3.12604	3.63628	-16.14437
H	-1.59730	0.54326	-13.98639
H	-3.00084	0.32240	-15.05458
C	-2.25432	1.05996	-14.70804
C	-4.47483	3.31158	-12.71972
C	-3.95854	4.28535	-13.82020
C	-2.83648	3.58865	-14.63823
C	-3.68178	2.04253	-12.99627
C	-4.67134	-2.40913	-10.96507
C	-4.75201	-3.78789	-10.93329
C	-4.94022	-8.01583	-10.76860
C	-5.09844	-9.23880	-10.55057
H	-2.89425	-11.76219	-9.90168
C	-3.42917	-11.97017	-10.84088
H	-3.07180	-11.26030	-11.60284
H	-5.39672	-11.40251	-12.75646
C	-5.69165	-12.12955	-11.98402
C	-4.96814	-11.82887	-10.64256
C	-6.31368	-12.28418	-7.19646
C	-7.97608	-12.51768	-9.03263
H	-6.28448	-8.80561	-8.05945
H	-7.54729	-8.96135	-9.30077
C	-6.99150	-9.51623	-8.52391
C	-5.48928	-12.80316	-9.55105
C	-6.54761	-12.06192	-8.69582
C	-5.41298	-10.47795	-10.11285
H	-5.89967	-13.72460	-9.98820
H	-2.51700	-4.48736	-13.48738
H	-2.39288	-2.02967	-13.52730
H	-4.93915	4.81803	-11.19353
H	-6.14291	2.63145	-13.98499
H	-6.56731	3.96348	-12.86918
H	-0.66915	3.68509	-14.92877
H	-1.45838	5.27516	-14.76647
H	-1.21517	4.24480	-13.32662
H	-4.08084	3.14010	-16.37962
H	-3.18833	4.68304	-16.48040
H	-2.33091	3.14866	-16.72931
H	-3.57809	5.21648	-13.37426
H	-4.78284	4.57297	-14.48785
H	-5.25483	-1.81875	-10.25549
H	-3.18401	-12.99232	-11.17031
H	-6.78393	-12.06988	-11.86701
H	-5.43422	-13.14142	-12.33814
H	-7.06295	-11.75502	-6.58650
H	-6.38656	-13.35666	-6.95607
H	-5.31476	-11.92849	-6.89960
H	-8.19030	-12.40201	-10.10580
H	-8.11015	-13.57761	-8.76581
H	-8.72310	-11.93300	-8.47223
H	-4.65232	-13.10222	-8.90126
C	-5.59148	-4.48906	-9.99262
C	-3.97497	-4.59156	-11.86812
H	-6.17026	-3.89766	-9.27808
C	-5.65228	-5.84637	-9.96288
C	-4.04195	-5.97055	-11.82851
H	-6.28396	-6.35926	-9.23444
H	-3.43547	-6.55802	-12.52091
C	-4.86381	-6.65435	-10.87885
C	4.63756	-3.32105	-14.19091
H	3.92331	-2.56462	-13.83243
H	5.11381	-3.77844	-13.30916
H	5.41516	-2.81436	-14.78460
H	-7.70336	-9.84590	-7.75432
H	-6.37097	2.29465	-12.25235
H	-4.67366	3.17634	-10.53156
H	-3.28093	4.15648	-11.05913
H	-1.64227	1.37063	-15.56568
H	-7.11875	-4.53957	-4.12339
H	-4.88672	0.72768	-4.93654
C	-4.87370	-4.45758	-2.61652
H	-5.33615	-4.90147	-1.72034
H	-4.84598	-5.21962	-3.40988
H	-3.83393	-4.18518	-2.37519
H	2.09748	-8.38543	-14.39355

Truncated 2^{dimer}, quintet

N	2.77736	-6.49537	-14.64388
N	-5.26216	-1.22226	-4.18020
C	-1.29729	-5.77152	-9.93915

C	-0.41989	-5.71423	-10.99233
C	0.09174	-4.45316	-11.46148
C	1.01375	-4.47140	-12.53113
C	1.86856	-4.66672	-13.40700
C	4.74526	-3.47052	-14.21996
C	3.29955	-3.62081	-16.29988
C	4.57867	-8.12392	-15.24494
C	3.17824	-7.12866	-17.03493
H	1.98339	-7.49144	-12.96513
H	0.79600	-7.05860	-14.21441
C	1.83516	-7.39735	-14.05679
C	3.86911	-4.42043	-15.09694
C	4.70453	-5.61229	-15.64120
C	3.80098	-6.86761	-15.65184
C	2.77612	-5.14091	-14.32425
C	-0.34436	-3.28415	-10.83293
C	-1.25747	-3.32520	-9.75314
C	-3.93634	-3.41494	-6.43531
C	-4.55320	-3.15682	-5.39072
C	-4.80912	-4.36151	-2.41021
C	-7.14385	-3.85660	-3.28817
C	-5.72889	-3.26580	-3.03746
C	-5.21081	0.40080	-2.27122
C	-7.38981	-0.32272	-3.20663
C	-4.80609	-0.35497	-5.22101
C	-5.86489	-2.04948	-2.07951
C	-5.94065	-0.76947	-2.93983
C	-5.15321	-2.60764	-4.28175
H	-6.73059	-2.14380	-1.40775
H	-1.68433	-6.73642	-9.60208
H	-0.09350	-6.62918	-11.49029
H	2.69508	-4.25884	-16.95915
H	4.12059	-3.18603	-16.89342
H	3.94389	-9.02346	-15.27012
H	5.41315	-8.28954	-15.94405
H	4.99331	-8.02025	-14.22997
H	2.61269	-6.25886	-17.39702
H	3.96378	-7.36226	-17.77074
H	2.48669	-7.98595	-16.99578
H	5.55117	-5.79740	-14.96120
H	5.12568	-5.40442	-16.63552
H	0.01370	-2.31497	-11.18643
H	-5.24253	-4.72205	-1.46362
H	-7.82885	-3.10380	-3.69976
H	-7.57069	-4.23786	-2.34603
H	-5.28713	1.32508	-2.86459
H	-5.65886	0.60309	-1.28591
H	-4.14417	0.16908	-2.12470
H	-7.98240	-1.11710	-3.67986
H	-7.88266	-0.03790	-2.26375
H	-7.40978	0.55329	-3.87510
H	-4.96535	-1.98953	-1.44626
C	-1.73339	-2.13991	-9.12040
C	-1.74372	-4.58869	-9.27698
H	-1.39700	-1.17163	-9.50015
C	-2.61235	-2.19635	-8.07013
C	-2.64642	-4.62959	-8.18725
H	-2.96844	-1.27854	-7.59964
H	-3.01042	-5.59737	-7.83779
C	-3.07956	-3.45756	-7.56022
N	-2.79195	2.25642	-14.04592
N	-6.25917	-10.61950	-9.08359
C	-3.09135	-3.83610	-12.78256
C	-3.04978	-2.46626	-12.79020
C	-3.82906	-1.69953	-11.86040
C	-3.77090	-0.29426	-11.97432
C	-3.74833	0.88613	-12.34638
C	-4.33207	3.98336	-11.27005
C	-5.93764	3.11965	-13.00872
C	-1.34910	4.28320	-14.25281
C	-2.92482	3.69904	-16.08170
C	-2.16987	1.10524	-14.62949
C	-4.43299	3.38275	-12.71001
C	-3.87097	4.34458	-13.79999
C	-2.71208	3.64309	-14.56318
C	-3.63223	2.11480	-12.94881
C	-4.62435	-2.37660	-10.93236
C	-4.68152	-3.78875	-10.91434
C	-4.80082	-8.04223	-10.78706
C	-4.99571	-9.24432	-10.56538
C	-3.45818	-12.08544	-10.87981
C	-5.72571	-12.11927	-12.01766
C	-4.98760	-11.86027	-10.67516
C	-6.33344	-12.27530	-7.22208
C	-8.02081	-12.39880	-9.04643
C	-6.86972	-9.46088	-8.50712
C	-5.55856	-12.81334	-9.58870
C	-6.56753	-12.02329	-8.71669
C	-5.35456	-10.49543	-10.12940
H	-6.02383	-13.70553	-10.03152
H	-2.47873	-4.40529	-13.48606
H	-2.41668	-1.93701	-13.50428
H	-4.94040	4.89887	-11.20138
H	-6.06743	2.71636	-14.02502
H	-6.51666	4.05443	-12.92591
H	-0.53251	3.72935	-14.74381
H	-1.32039	5.32363	-14.61336
H	-1.15589	4.28472	-13.16845
H	-3.86715	3.20578	-16.36798
H	-2.96930	4.74779	-16.41429
H	-2.10161	3.21419	-16.62889
H	-3.51400	5.28378	-13.35137
H	-4.66711	4.61896	-14.50649
H	-5.21326	-1.81074	-10.20808
H	-3.26862	-13.11647	-11.21859
H	-6.81378	-12.00765	-11.89816
H	-5.51810	-13.13941	-12.38135
H	-7.04983	-11.71687	-6.59881
H	-6.45839	-13.34558	-6.99365
H	-5.31517	-11.97453	-6.93017
H	-8.23547	-12.26280	-10.11718
H	-8.20996	-13.45225	-8.78727
H	-8.73151	-11.77946	-8.47598
H	-4.73571	-13.16641	-8.94796
C	-5.49185	-4.50148	-9.98130
C	-3.90338	-4.54536	-11.85307
H	-6.07589	-3.93233	-9.25334
C	-5.52269	-5.87037	-9.96518
C	-3.94394	-5.95923	-11.82300
H	-6.13669	-6.40222	-9.23625
H	-3.32936	-6.52286	-12.52732
C	-4.73246	-6.63869	-10.89192
H	-5.39797	-11.40208	-12.78657
H	2.65367	-2.79979	-15.94948
H	5.55701	-3.03730	-14.82583
H	-6.35365	2.38697	-12.29938

H	-7.09611	-4.68995	-4.00759
H	-4.70480	-5.21727	-3.09555
H	-5.07567	0.68426	-4.98887
H	-3.06452	-11.39035	-11.63776
H	-2.91001	-11.91428	-9.94063
H	-7.56613	-9.76169	-7.71180
H	1.94076	-8.39515	-14.50425
H	-1.51646	1.40985	-15.45865
H	-2.91780	0.38971	-15.01777
H	-1.55168	0.56397	-13.89248
H	4.13464	-2.64892	-13.81421
H	5.18636	-4.01881	-13.37379
H	-4.70186	3.25818	-10.52869
H	-3.28951	4.23099	-11.01541
H	-3.80471	-3.95806	-2.21173
H	-5.25694	-0.61584	-6.19498
H	-3.70881	-0.40591	-5.34637
H	-7.43302	-8.87811	-9.25852
H	-6.11716	-8.78387	-8.06519

Truncated 3^{dimer}, singlet

N	1.81581	-6.20869	-16.25556
N	-7.69945	-2.45218	-4.16782
C	-0.97826	-5.41563	-10.65237
C	-0.26658	-5.40713	-11.82123
C	-0.01306	-4.18054	-12.53650
C	0.60579	-4.22634	-13.75982
C	1.14835	-4.40049	-14.87385
H	4.10002	-3.36949	-16.30183
C	3.24925	-2.95145	-16.86103
C	1.03139	-3.45624	-18.00935
C	3.64225	-7.50150	-17.35265
C	1.45517	-7.23974	-18.50980
H	1.78707	-7.09158	-14.34404
H	0.20560	-6.98494	-15.14671
C	1.28189	-7.15788	-15.32431
C	2.22039	-4.06988	-17.21871
C	2.89349	-5.17584	-18.08054
C	2.44701	-6.56131	-17.55224
C	1.70308	-4.85318	-16.02378
C	-0.47849	-2.97004	-11.91952
C	-1.19836	-3.00027	-10.75515
C	-1.53071	-4.22149	-10.07744
C	-5.45531	-4.32003	-5.96828
C	-6.36318	-4.17221	-5.11957
H	-6.37164	-5.00801	-1.84902
C	-7.17311	-5.56150	-2.36110
C	-9.20265	-5.39958	-3.88282
C	-8.06450	-4.59356	-3.19960
C	-8.36270	-0.96412	-2.27096
C	-10.11035	-1.91178	-3.75759
H	-6.10945	-1.33494	-4.97973
H	-7.36856	-1.80496	-6.14134
C	-7.19806	-1.48533	-5.09764
C	-8.69505	-3.48753	-2.31181
C	-8.72994	-2.17696	-3.13323
C	-7.29740	-3.77224	-4.22478
H	-9.69418	-3.76776	-1.94835
H	-1.17072	-6.37817	-10.17893
H	0.09838	-6.34485	-12.24562
H	3.62568	-2.48201	-17.78366
H	0.31748	-4.23668	-18.31336
H	1.39724	-2.94650	-18.91560
H	3.32630	-8.48283	-16.96500
H	4.15289	-7.67306	-18.31332
H	4.36752	-7.06855	-16.64634
H	0.58309	-6.59546	-18.69996
H	1.94381	-7.45399	-19.47288
H	1.08938	-8.19426	-18.09884
H	3.98753	-5.09409	-17.99062
H	2.65457	-5.05963	-19.14712
H	-0.28073	-2.01703	-12.41523
H	-1.55543	-2.05173	-10.35740
H	-7.78626	-6.07964	-1.60685
H	-9.86314	-4.74618	-4.46941
H	-9.81086	-5.91715	-3.12294
H	-8.39753	-0.02739	-2.84900
H	-9.07811	-0.86574	-1.43957
H	-7.35205	-1.07274	-1.84738
H	-10.44543	-2.75514	-4.37773
H	-10.85989	-1.74353	-2.96839
H	-10.08666	-1.01374	-4.39561
H	-8.05935	-3.33044	-1.42620
C	-2.42743	-4.24740	-8.96557
C	-3.10660	-3.06640	-8.51210
C	-2.78263	-5.46274	-8.28828
H	-2.89524	-2.10818	-8.98376
H	-2.30110	-6.39666	-8.57743
C	-4.07371	-3.09740	-7.54545
C	-3.73791	-5.51063	-7.30799
H	-4.59327	-2.17899	-7.26492
H	-3.98813	-6.46290	-6.83484
C	-4.45169	-4.33130	-6.90310
N	-2.65694	3.61968	-12.37986
N	-5.00837	-11.74499	-9.26605
C	-3.91799	-2.54733	-12.43576
C	-3.86502	-1.18212	-12.37888
C	-4.62849	-0.44412	-11.40369
C	-4.45775	0.91214	-11.31518
C	-4.17303	2.12981	-11.32125
H	-3.33192	4.27487	-8.79521
C	-4.27185	4.59728	-9.26864
C	-5.45187	5.14526	-11.45274
C	-0.71356	5.18620	-12.30643
C	-2.65522	5.70253	-13.76974
H	-1.72785	1.74564	-12.59474
H	-2.94821	2.12082	-13.83102
C	-2.15177	2.56255	-13.20511
C	-4.11324	4.67268	-10.81902
C	-2.97711	5.64750	-11.23040
C	-2.23359	5.04141	-12.44741
C	-3.67013	3.37609	-11.47257
C	-5.49603	-1.20614	-10.54973
C	-5.52413	-2.57334	-10.61330
C	-4.71232	-3.32494	-11.52798
C	-4.13146	-8.95207	-11.04859
C	-4.10362	-10.17895	-10.80582
H	-1.60544	-12.50029	-10.42080
C	-2.19300	-12.71994	-11.32526
H	-4.35239	-12.27054	-13.05654
C	-4.51654	-13.05220	-12.29873
C	-3.71831	-12.73562	-11.00358
C	-4.78048	-13.48598	-7.49261
C	-6.51955	-13.74361	-9.25132
H	-4.94350	-9.93764	-8.19983

H	-6.39722	-10.16008	-9.19664
C	-5.66878	-10.68975	-8.55623
C	-4.05338	-13.79418	-9.91759
C	-5.11335	-13.19411	-8.96070
C	-4.24085	-11.45500	-10.37666
H	-4.39923	-14.73983	-10.35886
H	-3.29397	-3.05181	-13.17179
H	-3.21769	-0.62994	-13.06360
H	-4.55026	5.58557	-8.86993
H	-5.36735	5.22872	-12.54584
H	-5.73918	6.13030	-11.04940
H	-0.18165	4.76325	-13.17309
H	-0.44243	6.25156	-12.23944
H	-0.35309	4.67845	-11.39836
H	-3.74347	5.64637	-13.91897
H	-2.35980	6.76334	-13.77716
H	-2.17010	5.21177	-14.62886
H	-2.26611	5.74790	-10.39543
H	-3.35817	6.65485	-11.45130
H	-6.11302	-0.67688	-9.81987
H	-6.17248	-3.10237	-9.91618
H	-1.88190	-13.69961	-11.72135
H	-5.59669	-13.10168	-12.09778
H	-4.19504	-14.01994	-12.71755
H	-5.54036	-13.07006	-6.81258
H	-4.74596	-14.57377	-7.32414
H	-3.80212	-13.06096	-7.21907
H	-6.80599	-13.57718	-10.30038
H	-6.55507	-14.82571	-9.05050
H	-7.27436	-13.25588	-8.61358
H	-3.14336	-14.02217	-9.34108
C	-4.63227	-4.74824	-11.47594
C	-5.32088	-5.51511	-10.47667
C	-3.79406	-5.49838	-12.36740
H	-5.96252	-5.00944	-9.75617
H	-3.23665	-4.97821	-13.14547
C	-5.15435	-6.86529	-10.34484
C	-3.62311	-6.85150	-12.25695
H	-5.66790	-7.40383	-9.54585
H	-2.95411	-7.37577	-12.94276
C	-4.28372	-7.60196	-11.22627
H	-1.36339	2.94694	-13.86652
H	2.77810	-2.17502	-16.23848
H	0.48741	-2.72490	-17.39152
H	-6.25840	4.42855	-11.23381
H	-5.05760	3.87515	-8.99789
H	-8.78646	-6.15384	-4.56900
H	-6.70589	-6.31592	-3.01274
H	-7.69912	-0.52010	-4.94409
H	-6.20283	-11.09739	-7.68707
H	-1.96567	-11.94922	-12.07799
H	1.41228	-8.17756	-15.71110

Truncated 3^{dimer}, triplet

N	1.57734	-5.98312	-16.43391
N	-7.80637	-2.47196	-4.25502
C	-1.41481	-5.30592	-10.91563
C	-0.67181	-5.27353	-12.08489
C	-0.06037	-4.06723	-12.52426
C	0.65136	-4.08354	-13.73631
C	1.22910	-4.26582	-14.81649
H	4.48715	-3.79664	-15.81968
C	3.82457	-3.12392	-16.38558
C	1.72641	-2.93453	-17.80121
C	3.10027	-7.56598	-17.61735
C	1.22837	-6.49380	-18.85827
H	1.02502	-7.04531	-14.70441
H	-0.33658	-6.46404	-15.68814
C	0.69059	-6.86946	-15.74210
C	2.62591	-3.91420	-16.99804
C	3.12113	-5.04058	-17.94668
C	2.24428	-6.29765	-17.72243
C	1.79803	-4.68759	-15.98966
C	-0.22168	-2.91071	-11.72092
C	-0.97894	-2.95999	-10.55913
C	-1.60255	-4.14962	-10.13121
C	-5.41212	-4.31113	-5.86605
C	-6.30252	-4.15608	-5.01907
H	-6.29540	-4.63300	-1.64068
C	-7.03172	-5.30971	-2.10065
C	-9.02804	-5.50611	-3.65677
C	-7.98794	-4.52327	-3.05133
C	-8.59730	-0.85671	-2.52077
C	-10.25375	-2.08510	-3.90598
H	-6.32553	-1.33557	-5.23044
H	-7.56207	-2.03219	-6.30004
C	-7.39948	-1.58557	-5.30247
C	-8.74026	-3.39054	-2.30132
C	-8.86341	-2.17596	-3.25522
C	-7.28182	-3.75208	-4.15019
H	-9.72310	-3.71952	-1.93424
H	-1.91657	-6.23275	-10.63464
H	-0.57225	-6.17317	-12.69469
H	4.40730	-2.64119	-17.18639
H	0.89355	-3.46690	-18.28400
H	2.31384	-2.42550	-18.58325
H	2.48083	-8.46294	-17.45876
H	3.66810	-7.71628	-18.54906
H	3.81505	-7.48817	-16.78347
H	0.60155	-5.59947	-18.99258
H	1.75019	-6.69555	-19.80673
H	0.56182	-7.34705	-18.65425
H	4.16547	-5.28782	-17.70062
H	3.10725	-4.72390	-18.99926
H	0.24741	-1.97525	-12.03271
H	-1.09713	-2.05150	-9.96520
H	-7.61059	-5.80108	-1.30261
H	-9.73673	-4.98254	-4.31412
H	-9.59909	-6.00440	-2.85591
H	-8.69009	0.01018	-3.19389
H	-9.32853	-0.72450	-1.70796
H	-7.58692	-0.84773	-2.08294
H	-10.51286	-3.01267	-4.43649
H	-11.02285	-1.89863	-3.14010
H	-10.29558	-1.25807	-4.63286
H	-8.15050	-3.08841	-1.42138
C	-2.49918	-4.17556	-8.96058
C	-3.29764	-3.05880	-8.63978
C	-2.65934	-5.33993	-8.18219
H	-3.22396	-2.15327	-9.24318
H	-2.04190	-6.21469	-8.39599
C	-4.23974	-3.11114	-7.62444
C	-3.58910	-5.40741	-7.15512
H	-4.87498	-2.24661	-7.42431
H	-3.69853	-6.32332	-6.57042
C	-4.41953	-4.29731	-6.86089

N	-2.60021	3.57153	-12.32910
N	-4.73812	-11.80826	-9.04513
C	-3.77292	-2.73739	-12.38064
C	-3.68422	-1.35937	-12.33713
C	-4.58682	-0.59545	-11.53895
C	-4.42609	0.78766	-11.46451
C	-4.16778	2.00145	-11.45834
H	-3.71437	4.21220	-8.88604
C	-4.61814	4.48090	-9.45408
C	-5.60146	4.94703	-11.74986
C	-0.79241	5.27411	-12.07476
C	-2.58128	5.61981	-13.76802
H	-1.54675	1.75269	-12.35711
H	-2.58292	2.03507	-13.77348
C	-1.91903	2.53338	-13.04372
C	-4.30840	4.55585	-10.98265
C	-3.19494	5.59629	-11.28577
C	-2.27116	5.01809	-12.38752
C	-3.71545	3.28459	-11.55982
C	-5.59894	-1.29743	-10.82718
C	-5.66238	-2.67709	-10.87103
C	-4.74450	-3.44382	-11.63015
C	-4.28537	-9.10232	-11.11587
C	-4.21188	-10.30449	-10.81672
H	-1.59708	-12.51778	-10.82492
C	-2.34183	-12.81333	-11.57959
C	-4.79035	-13.30367	-12.04279
C	-3.77187	-12.86640	-10.95399
C	-4.15900	-13.46054	-7.26394
C	-6.16013	-13.83553	-8.68989
H	-4.52622	-9.94866	-8.09057
H	-6.14004	-10.25626	-8.76880
C	-5.28048	-10.73571	-8.26551
C	-3.83590	-13.85907	-9.76068
C	-4.74138	-13.24343	-8.66452
C	-4.21579	-11.56913	-10.30517
H	-4.19264	-14.85267	-10.06770
H	-3.02387	-3.29272	-12.94661
H	-2.89219	-0.84406	-12.88378
H	-4.98728	5.45457	-9.09454
H	-5.41061	5.04257	-12.82867
H	-5.99125	5.90924	-11.37862
H	-0.13423	4.87618	-12.86306
H	-0.60516	6.35709	-12.00378
H	-0.50658	4.80890	-11.11844
H	-3.63789	5.47798	-14.03895
H	-2.36523	6.69960	-13.77182
H	-1.96547	5.14900	-14.55129
H	-2.59815	5.76311	-10.37541
H	-3.61070	6.57052	-11.58041
H	-6.31675	-0.73067	-10.23023
H	-6.44027	-3.18815	-10.30087
H	-2.06817	-13.80223	-11.98052
H	-5.80854	-13.37073	-11.63354
H	-4.51401	-14.28880	-12.45349
H	-4.80881	-13.03654	-6.48233
H	-4.06039	-14.53856	-7.06196
H	-3.16349	-12.99831	-7.17577
H	-6.62350	-13.72881	-9.68137
H	-6.13285	-14.90645	-8.43433
H	-6.81028	-13.33007	-7.95764
H	-2.82379	-13.99240	-9.34727
C	-4.72719	-4.90289	-11.56467
C	-5.17156	-5.58409	-10.40516
C	-4.17943	-5.68747	-12.60870
H	-5.56197	-5.01225	-9.56253
H	-3.84769	-5.19783	-13.52598
C	-5.03229	-6.95125	-10.26997
C	-4.04812	-7.05862	-12.49890
H	-5.33426	-7.44262	-9.34349
H	-3.61884	-7.63896	-13.31842
C	-4.44882	-7.73164	-11.31181
H	1.29414	-2.16924	-17.13751
H	3.46012	-2.34419	-15.69864
H	0.65309	-7.83884	-16.25754
H	-4.81087	-12.57527	-12.86858
H	-2.30851	-12.08046	-12.40067
H	-5.61801	-11.11230	-7.29026
H	-7.97278	-0.65000	-5.24631
H	-6.48290	-6.08224	-2.66132
H	-8.52593	-6.28010	-4.25868
H	-6.38027	4.18021	-11.61466
H	-5.38794	3.71922	-9.25501
H	-1.06116	2.95228	-13.58731

Truncated 3^{dimer}, quintet

N	1.56152	-5.95451	-16.43260
N	-7.84436	-2.43279	-4.28242
C	-1.44783	-5.27687	-10.91751
C	-0.70756	-5.24513	-12.08895
C	-0.06149	-4.04958	-12.50828
C	0.65054	-4.06641	-13.72001
C	1.22895	-4.24865	-14.79974
C	3.83661	-3.11598	-16.35112
C	1.74552	-2.89952	-17.77308
C	3.07093	-7.54385	-17.62425
C	1.21785	-6.43818	-18.86394
H	0.99990	-7.02500	-14.71145
H	-0.35638	-6.42360	-15.69131
C	0.66681	-6.83834	-15.74753
C	2.63442	-3.89230	-16.97422
C	3.12339	-5.01593	-17.92908
C	2.23043	-6.26464	-17.72135
C	1.79606	-4.66632	-15.97515
C	-0.18654	-2.90257	-11.68466
C	-0.94278	-2.95005	-10.52220
C	-1.59893	-4.12848	-10.11414
C	-5.41135	-4.25916	-5.85349
C	-6.30790	-4.10173	-5.01374
C	-7.03607	-5.22509	-2.08195
C	-9.01135	-5.48224	-3.65569
C	-7.99831	-4.47059	-3.05212
C	-8.66387	-0.80976	-2.56982
C	-10.29845	-2.07213	-3.95116
H	-6.37583	-1.29557	-5.27462
H	-7.60365	-2.02481	-6.33429
C	-7.44688	-1.55921	-5.34437
C	-8.78067	-3.34325	-2.32470
C	-8.91093	-2.13959	-3.29196
C	-7.29755	-3.70111	-4.15519
H	-9.76315	-3.68565	-1.96920
H	-1.97507	-6.19353	-10.64917
H	-0.63641	-6.13621	-12.71505
H	4.42616	-2.63176	-17.14599
H	0.90995	-3.42135	-18.26253
H	2.33965	-2.38946	-18.54935

H	2.44033	-8.43510	-17.47801
H	3.64205	-7.69146	-18.55438
H	3.78211	-7.48263	-16.78592
H	0.60228	-5.53537	-18.99324
H	1.74216	-6.63740	-19.81153
H	0.54032	-7.28534	-18.67093
H	4.16290	-5.27745	-17.67728
H	3.12094	-4.69026	-18.97900
H	0.31066	-1.97668	-11.98105
H	-1.03510	-2.04979	-9.91099
H	-7.61263	-5.71721	-1.28273
H	-9.72510	-4.98053	-4.32476
H	-9.57897	-5.98399	-2.85455
H	-8.76029	0.04863	-3.25329
H	-9.40235	-0.67611	-1.76382
H	-7.65685	-0.78576	-2.12491
H	-10.54264	-3.00842	-4.47366
H	-11.07387	-1.88814	-3.19114
H	-10.34713	-1.25291	-4.68639
H	-8.20987	-3.02305	-1.43876
C	-2.49553	-4.14568	-8.94346
C	-3.30331	-3.03037	-8.64236
C	-2.64659	-5.30014	-8.14928
H	-3.23492	-2.13427	-9.26091
H	-2.02182	-6.17257	-8.35207
C	-4.24673	-3.07548	-7.62766
C	-3.57653	-5.35933	-7.12157
H	-4.89027	-2.21395	-7.44160
H	-3.67952	-6.26671	-6.52257
C	-4.41670	-4.25139	-6.84619
N	-2.63424	3.57254	-12.30513
N	-4.70969	-11.80666	-9.04204
C	-3.74611	-2.75046	-12.34029
C	-3.66613	-1.36710	-12.29291
C	-4.60428	-0.61364	-11.53615
C	-4.45418	0.78002	-11.45637
C	-4.20160	1.99179	-11.44636
C	-4.67609	4.48246	-9.44730
C	-5.65515	4.93236	-11.74698
C	-0.84095	5.28799	-12.03733
C	-2.61236	5.61394	-13.75310
H	-1.58010	1.75475	-12.32877
H	-2.59833	2.04281	-13.75742
C	-1.94453	2.53799	-13.01618
C	-4.36144	4.55165	-10.97545
C	-3.25480	5.59962	-11.27908
C	-2.31422	5.02070	-12.36638
C	-3.75647	3.28290	-11.54377
C	-5.64157	-1.31504	-10.87143
C	-5.70176	-2.69937	-10.92474
C	-4.74944	-3.45288	-11.64139
C	-4.28156	-9.12689	-11.14893
C	-4.20825	-10.32378	-10.84218
C	-2.36404	-12.85513	-11.60919
C	-4.82138	-13.34568	-12.02061
C	-3.78289	-12.89465	-10.95668
C	-4.10979	-13.44155	-7.25124
C	-6.13487	-13.82299	-8.64053
H	-4.47339	-9.93902	-8.10859
H	-6.09949	-10.24479	-8.75985
C	-5.23428	-10.72349	-8.26553
C	-3.82799	-13.86998	-9.74796
C	-4.71324	-13.23705	-8.64485
C	-4.20737	-11.58776	-10.31655
H	-4.19112	-14.86760	-10.03391
H	-2.97297	-3.30646	-12.87286
H	-2.85429	-0.84785	-12.80537
H	-5.05363	5.45506	-9.09348
H	-5.46096	5.02830	-12.82530
H	-6.05438	5.89180	-11.37833
H	-0.17081	4.88898	-12.81501
H	-0.66092	6.37250	-11.97094
H	-0.56418	4.83044	-11.07468
H	-3.66487	5.46262	-14.03508
H	-2.40477	6.69539	-13.75920
H	-1.98414	5.14461	-14.52736
H	-2.66866	5.78198	-10.36481
H	-3.67735	6.56666	-11.58742
H	-6.38438	-0.75170	-10.30271
H	-6.50062	-3.21837	-10.39100
H	-1.60465	-12.55342	-10.87172
H	-2.10076	-13.84931	-12.00418
H	-5.83203	-13.40454	-11.59183
H	-4.55501	-14.33711	-12.42292
H	-4.74498	-13.00547	-6.46423
H	-4.01327	-14.51773	-7.03864
H	-3.11072	-12.98349	-7.18416
H	-6.61419	-13.72428	-9.62520
H	-6.10834	-14.89140	-8.37437
H	-6.77054	-13.30707	-7.90284
H	-2.80899	-13.99846	-9.35014
C	-4.72982	-4.92565	-11.57952
C	-5.10346	-5.59555	-10.39639
C	-4.24688	-5.70431	-12.65068
H	-5.44440	-5.01785	-9.53622
H	-3.96524	-5.21222	-13.58397
C	-4.96510	-6.96830	-10.26909
C	-4.10870	-7.08028	-12.54426
H	-5.22061	-7.45851	-9.32829
H	-3.72463	-7.66280	-13.38426
C	-4.44531	-7.74553	-11.33922
H	3.47531	-2.33864	-15.65986
H	1.31734	-2.13571	-17.10505
H	0.62054	-7.80296	-16.27112
H	-1.07980	2.96000	-13.54648
H	-5.44119	3.71629	-9.24702
H	-6.42856	4.15960	-11.61393
H	-8.03155	-0.62980	-5.30391
H	-8.48737	-6.25160	-4.24470
H	-6.31739	-4.52801	-1.62443
H	-5.55910	-11.08933	-7.28183
H	-2.34332	-12.13174	-12.43914
H	-4.85571	-12.62899	-12.85626
H	-3.77241	4.22290	-8.87485
H	4.49179	-3.79800	-15.78774
H	-6.46714	-5.99416	-2.62708

Section S9. Supplementary References

1 Hansmann, M. M., Melaimi, M., Munz, D. & Bertrand, G. Modular Approach to Kekule Diradicaloids Derived from Cyclic (Alkyl)(amino)carbenes. *J. Am. Chem. Soc*. 140, 2546-2554, (**2018**).

2 Neese, F. Software update: the ORCA program system, version 4.0. *Wiley Interdiscip. Rev. Comput. Mol. Sci*. 8, e1327, (**2018**).

3 Becke, A. D. Density-functional thermochemistry. III. The role of exact exchange. *J. Chem. Phys*. 98, 5648-5652, (**1993**).

4 Lee, C., Yang, W. & Parr, R. G. Development of the Colle-Salvetti correlation-energy formula into a functional of the electron density. *Phys. Rev. B Condens. Matter* 37, 785-789, (**1988**).

5 Stephens, P. J., Devlin, F. J., Chabalowski, C. F. & Frisch, M. J. Ab-Initio Calculation of Vibrational Absorption and Circular-Dichroism Spectra Using Density-Functional Force-Fields. *J. Phys. Chem. A* 98, 11623-11627, (**1994**).

6 Vosko, S. H., Wilk, L. & Nusair, M. Accurate Spin-Dependent Electron Liquid Correlation Energies for Local Spin-Density Calculations - a Critical Analysis. *Can J Phys* 58, 1200-1211, (**1980**).

7 Grimme, S., Antony, J., Ehrlich, S. & Krieg, H. A consistent and accurate ab initio parametrization of density functional dispersion correction (DFT-D) for the 94 elements H-Pu. *J. Chem. Phys*. 132, 154104, (**2010**).

8 Grimme, S., Ehrlich, S. & Goerigk, L. Effect of the damping function in dispersion corrected density functional theory. *J. Comput. Chem*. 32, 1456-1465, (**2011**).

9 Messelberger, J., Grünwald, A., Pinter, P., Hansmann, M. M. & Munz, D. Carbene derived diradicaloids – building blocks for singlet fission? *Chem. Sci.* 9, 6107-6117, (**2018**).

10 Weigend, F. & Ahlrichs, R. Balanced basis sets of split valence, triple zeta valence and quadruple zeta valence quality for H to Rn: Design and assessment of accuracy. *Phys. Chem. Chem. Phys*. 7, 3297-3305, (**2005**).

11 Izsak, R. & Neese, F. An overlap fitted chain of spheres exchange method. *J. Chem. Phys*. 135, 144105, (**2011**).

12 Neese, F., Wennmohs, F., Hansen, A. & Becker, U. Efficient, approximate and parallel Hartree–Fock and hybrid DFT calculations. A 'chain-of-spheres' algorithm for the Hartree–Fock exchange. *Chem. Phys*. 356, 98-109, (**2009**).

13 Weigend, F. Hartree-Fock exchange fitting basis sets for H to Rn. *J. Comput. Chem*. 29, 167-175, (**2008**).

14 Roos, B. O., Taylor, P. R. & Siegbahn, P. E. M. A Complete Active Space Scf Method (Casscf) Using a Density-Matrix Formulated Super-Ci Approach. *Chem. Phys*. 48, 157-173, (**1980**).

15 Lenthe, E. v., Baerends, E. J. & Snijders, J. G. Relativistic regular two-component Hamiltonians. *J. Chem. Phys*. 99, 4597-4610, (**1993**).

16 van Lenthe, E., Baerends, E. J. & Snijders, J. G. Relativistic total energy using regular approximations. *J. Chem. Phys*. 101, 9783-9792, (**1994**).

17 van Lenthe, E., Ehlers, A. & Baerends, E.-J. Geometry optimizations in the zero order regular approximation for relativistic effects. *J. Chem. Phys*. 110, 8943-8953, (**1999**).

18 Pantazis, D. A., Chen, X. Y., Landis, C. R. & Neese, F. All-Electron Scalar Relativistic Basis Sets for Third-Row Transition Metal Atoms. *J. Chem. Theory Comput*. 4, 908-919, (**2008**).

19 Pantazis, D. A. & Neese, F. All-electron scalar relativistic basis sets for the 6p elements. *Theoretica Chimica Acta* 131, 1292, (**2012**).

20 Angeli, C., Cimiraglia, R., Evangelisti, S., Leininger, T. & Malrieu, J. P. Introduction of n-electron valence states for multireference perturbation theory. *J. Chem. Phys*. 114, 10252-10264, (**2001**).

21 Hanwell, M. D. et al. Avogadro: an advanced semantic chemical editor, visualization, and analysis platform. *J. Cheminformatics* 4, 17, (**2012**).
22 Knizia, G. Intrinsic Atomic Orbitals: An Unbiased Bridge between Quantum Theory and Chemical Concepts. *J. Chem. Theory Comput*. 9, 4834-4843, (**2013**).
23 Legault, C. CYLview, 1.0 b. Université de Sherbrooke, (**2009**).
24 D. Casanova, *Chem Rev* (**2018**), *118*, 7164-7207.
25 H. Nakamura, D. G. Truhlar, *J. Chem. Phys.* (**2001**), *115*, 10353-10372.
26 H. Nakamura, D. G. Truhlar, *J. Chem. Phys.* (**2002**), *117*, 5576-5593.
27 Tamura, H., Huix-Rotllant, M., Burghardt, I., Olivier, Y. & Beljonne, D. First-Principles Quantum Dynamics of Singlet Fission: Coherent versus Thermally Activated Mechanisms Governed by Molecular π Stacking. *Phys. Rev. Lett.* 115, 107401, (**2015**).
28 M. E. Sandoval-Salinas, A. Carreras, J. Casado, D. Casanova, *J. Chem. Phys.* (**2019**), *150*, 204306.
29 A. B. Kolomeisky, X. Feng, A. I. Krylov, *J. Chem. Phys.* (**2014**), *118*, 5188-5195.
